# Non-target screening reveals 124 PFAS at an AFFF-impacted field site in Germany specified by novel systematic terminology

**DOI:** 10.1007/s00216-024-05611-3

**Published:** 2024-10-28

**Authors:** Melanie Schüßler, Catharina Capitain, Boris Bugsel, Jonathan Zweigle, Christian Zwiener

**Affiliations:** Environmental Analytical Chemistry, Department of Geosciences, University of Tübingen, Schnarrenbergstraße 94-96, 72076 Tübingen, Germany

**Keywords:** AFFF, PFAS, Terminology, HRMS, Non-target screening, Identification, Soil

## Abstract

The uncontrolled release of aqueous film-forming foam (AFFF) ingredients during a major fire incident in Reilingen, Germany, in 2008 led to significant soil and groundwater contamination. As the identity of fluorochemical surfactants in AFFF are often veiled due to company secrets, it is important to characterize AFFF contaminations and their impact on the environment comprehensively. In this study, we adapted a systematic approach combining a suitable extraction method with liquid chromatography high-resolution quadrupole time-of-flight mass spectrometry (LC-QTOF-MS) for an extensive non-targeted analysis. Our analysis identified 124 per- and polyfluoroalkyl substances (PFAS) from 42 subclasses in the contaminated soil (confidence levels of identification between 1 and 3). Typical for AFFF-impacted field sites, these included anionic, cationic, and zwitterionic substances with perfluoroalkyl chains spanning from 3 to 14 carbon atoms. Furthermore, we identified 1 previously unreported substance, and detected 9 PFAS subclasses for the first time in soil. AFFFs have long been employed to extinguish large hydrocarbon fires, yet their environmental consequences remain a concern. This study sheds light on the complex composition of AFFFs at this particularly contaminated area, emphasizing the necessity for extensive contaminant characterization as sound basis for informed management strategies to mitigate their adverse effects. AFFF PFAS are often named differently in the literature, leading to inconsistency in terminology. To address this issue, we introduced partially new terminology for AFFF-related PFAS to establish consistent terminology, to facilitate communication of identified compounds, and to ensure that the chemical structure can be directly derived from acronyms.

**Graphical Abstract:**

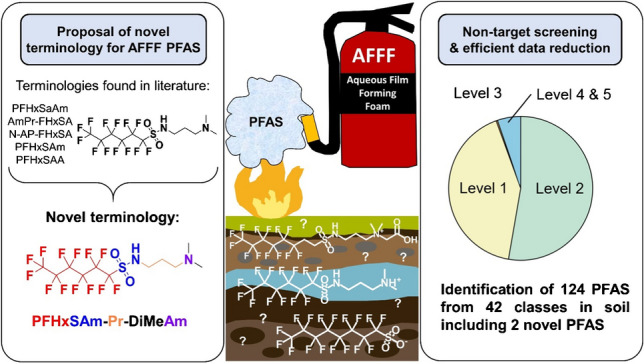

**Supplementary Information:**

The online version contains supplementary material available at 10.1007/s00216-024-05611-3.

## Introduction

In 2021, a new definition for the group of per- and polyfluoroalkyl substances (PFAS) was released by the Organisation for Economic Co-Operation and Development (OECD), which categorizes all substances with a CF_2_ or CF_3_ group (excluded CF_2_H and CF_2_-halogens) as PFAS, resulting in more than 7 million single substances that fall into the PFAS category [1–3]. Another definition of PFAS by Buck et al. [4] from 2011 includes all compounds with perfluoroalkyl moieties (C_n_F_2n+1_), where *n* denotes the number of carbon atoms. This results in more than 4700 substances [4]. Due to an increasing awareness of PFAS even outside the research community and extensive application combined with human and environmental health concerns, the European Union aspires to a ban of the entire PFAS class [5]. Earlier regulations of PFAS on international level comprise the three popular PFAS representatives, perfluorooctanoic acid (PFOA), perfluorooctane sulfonic acid (PFOS), and perfluorohexane sulfonic acid (PFHxS), into the Stockholm Convention of persistent organic pollutants [6].

These regulations were enacted since various PFAS are increasingly associated with adverse human health effects such as thyroid disorder, liver damage, kidney and testicular cancer, and lower birth weight, [7] as well as environmental concerns due to potential bioaccumulation [8–10] and high persistence in the environment [11, 12].

However, due to their unique chemistry, PFAS show attractive properties for industrial applications such as high surface activity, hydro- and oleophobicity, as well as high chemical and thermal stability [13]. Produced since the 1950s, PFAS are now used in nearly every branch of industry and are ingredients in numerous everyday consumer products [14].

A major field of PFAS application is the use in fluorosurfactant-based firefighting foams such as aqueous film-forming foams (AFFFs), which are used for extinguishing class B fires caused by hydrocarbon-based liquids [15]. PFAS ensure stability of the foam and the formation of a water film between the burning material and the foam, preventing reignition [14]. AFFFs are produced by various manufacturers and formulations differ in composition [16, 17]. In the past, perfluorocarboxylic and sulfonic acids (PFCAs and PFSAs) and their derivatives were often employed in AFFF formulations and PFOS was the substantial component in the AFFF brand Lightwater from 3M, a former major AFFF producer, until 3M phased-out PFOS in the early 2000s [15, 18–20]. 3M ceased AFFF production in 2002 but was known to utilize both perfluorinated and polyfluorinated substances generated by electrochemical fluorination (ECF) over the years, while other manufacturers usually employ polyfluorinated fluorotelomer-based PFAS in their formulations [16, 20, 21]. Common, well-known AFFF PFAS include perfluoroalkyl sulfonamides propyl dimethylamines (PFASAm-Pr-DiMeAms, e.g., in 3M AFFF, Angus), fluorotelomer sulfonamide propyl betaines (FTSAm-Pr-Bs, e.g., in National Foam, Fireade, Angus), and n:1:2 fluorotelomer betaines (n:1:2 FTBs, e.g., in Buckeye, Ansulite) (Electronic Supplementary Material, ESM 2). Due to the phase-out from C_8_-based PFAS chemistry, the trend in AFFF formulations shifted towards short-chain PFAS [14]. As AFFF formulations do not have to be disclosed by manufacturers, large fractions of PFAS in AFFFs remain unknown [16, 17]. Since AFFFs are directly discharged into the environment during a fire or training event, sites impacted by AFFFs are often heavily contaminated [18, 22, 23].

Numerous studies showed that various AFFF PFAS can ultimately transform to legacy PFAS such as perfluoroalkyl acids (PFAAs), fluorotelomer sulfonic acids (FTSAs), or fluorotelomer sulfonamides (FTSAms) by photochemical oxidation [24, 25] or biodegradation [25–28]. PFAAs are not further transformed under environmental conditions and can thus accumulate in soil and groundwater over time [29]. While short-chain PFAS are more mobile and can migrate into groundwater, longer chain PFAS are mostly retained in the top layers of soil [29, 30]. This is especially true for zwitterionic and cationic AFFF compounds due to electrostatic interaction with negatively charged soil particles [31, 32]. Thus, for risk assessment and remediation management of AFFF-impacted field sites, a comprehensive characterization of the contaminant pattern is crucial. To accurately characterize the contamination pattern in AFFF-impacted soils, it is essential to select an appropriate extraction method. A simple methanolic extraction, as is often used for soil samples contaminated with anionic PFAS, is insufficient for cationic and zwitterionic AFFF-PFAS [33]. Thus, more exhaustive methods have been developed for soils contaminated with AFFF, employing the combination of acidic and basic extraction solvents [33] or various additives to methanol [34].

Non-target screening (NTS) by high-resolution mass spectrometry (HRMS) is an important tool to identify unknown PFAS in AFFF formulations and impacted field sites, especially since analytical reference standards are often not commercially available which considerably limits target analysis [35]. NTS approaches enabled to identify novel PFAS in AFFF formulations [17, 21, 23, 36, 37] which were then also increasingly detected in groundwater [22, 23, 38], surface water [15], and soils [33, 38, 39] from AFFF-impacted field sites.

One of the challenges in NTS is to achieve appropriate data reduction without losing features of interest. Various strategies for PFAS have been described that utilize the intrinsic compound properties [40]. Since PFAS often occur in homologous series with characteristic repeating units of CF_2_, C_2_F_4_, or CF_2_O moieties, Kendrick-mass defect analysis can be applied [41]. Mass defects of PFAS usually fall into a range from − 0.2 to + 0.1 Da which also offers a filtering possibility. Furthermore, it was shown that the novel MD/C-m/C approach, where for each substance the mass defect per carbon number (MD/C) is plotted against the mass per carbon number (m/C), can reliably separate potential PFAS from many common organic compounds coextracted from the sample matrix [42, 43]. On the MS^2^ level, diagnostic fragments or recurring fragment mass differences can be utilized for identification and structure elucidation [44].

The terminology of PFAS has largely followed the conventions established by Buck et al. [4]. However, typical PFAS in AFFF formulations are anionic, cationic, or zwitterionic and can contain multiple functional groups and novel structures are frequently employed, diverging from those described by Buck et al. [4]. These AFFFs often receive varied, inconsistent acronyms and names in the literature. For instance, the major AFFF component Capstone B/Forafac 1157 (CAS 34455–29-3) is commonly abbreviated as 6:2 FTAB [25], 6:2 FTSaB [22], or CMAmP-6:2 FASA [23]. To address this issue, we propose a consistent and easy identifiable system for acronyms of AFFF PFAS and their transformation products (TPs) based on the terminology of Buck et al. [4].

In Reilingen, southwest Germany, a major fire incident occurred in a mattress warehouse in 2008. Various fire departments from the region were involved in firefighting activities, which led to a massive deployment of different AFFF formulations. The AFFFs drained into an irrigation trench across the street and an adjacent agricultural area. An extensive characterization of AFFF PFAS in this area was still pending. The goals of this study were therefore to (i) identify the prevailing PFAS contamination in soil and groundwater by target and non-target screening and to (ii) introduce consistent and identifiable acronyms for AFFF PFAS. This includes by-products and transformation products of AFFF compounds.

## Materials and methods

### Chemicals and reagents

Methanol (MeOH), ammonium acetate (NH_4_Ac), and water were of optima LC–MS grade and were purchased from Thermo Fisher Scientific. A PFAS standard mixture containing 51 authentic reference standards was used for identification of level 1 substances. Detailed information on the reference standards can be found in the ESM 1.A (Table S1).

### Sampling and processing of soil and water samples

Soil and groundwater samples were taken in May 2023. Soil samples (*S1*) were collected using drill core probing to a depth of 3 m within the *Nachtwaidgraben* field site (for detailed information, see ESM 1.B, Fig. S1). Four drill cores were obtained at location *S1* (*S1-1*, *S1-2*, *S1-3*, *S1-4*) arranged on a 1 × 1 m square. Drill cores were divided vertically into six sections of 0.5 m each and composite samples of the four drill cores were prepared for each depth section. In this study, only the first depth section (*S1 0–0.5 m*) is discussed. Composite samples were dried at 40 °C, ground, and sieved to a remaining fraction of ≤ 1.6 mm. The water content was 12.6 w/w%. A groundwater sample was taken from a monitoring well along the roadside (*GW*, Fig. S1). Prior to analysis, the water sample was centrifuged for 30 min at 20,817 relative centrifugal forces (rcf) and decanted.

To determine the most suitable extraction method for the soil, four different methods were tested: a simple methanolic extraction, two extractions with methanol plus additives (MeOH + 0.1 M NH_4_OH and MeOH + 0.4 M NH_4_Ac [34]), and the extraction method described by Nickerson et al. [33], which combines acidic and basic extraction solutions. Sequential extraction with MeOH + 0.4 M NH_4_Ac was chosen as the final extraction method. Five grams of dried soil was weighed into 40-mL centrifuge tubes and mixed with 5 mL of the extraction agent. The suspensions were vortexed for 1 min, sonicated for 15 min, and placed on an overhead shaker for approximately 24 h. The tubes were centrifuged for 15 min at 7197 rcf, and the supernatant was transferred and centrifuged again for 30 min at 20817 rcf. A second and third extraction was processed likewise, but with 3 mL extraction agent only as the soil was already soaked. Extracts from multiple extractions were combined with proportional shares (0.5, 0.3, and 0.3 mL). An extraction blank consisting of pure extraction solvent was processed the same way as the soil samples to check for background contamination during the extraction process.

### Instrumental analysis

Soil extracts and the groundwater sample were analyzed using high-performance liquid chromatography (1290 HPLC from Agilent Technologies, Waldbronn, Germany) coupled to a quadrupole time-of-flight mass spectrometer with an electrospray ionization source (6550 QTOF, Agilent Technologies, Santa Clara, USA, HPLC-ESI-QTOF-MS). Separation was achieved on an Agilent C_18_ column (Poroshell 120 EC-C_18_, 2.1 mm × 100 mm, particle size 2.7 µm) with a flow rate of 0.3 mL/min and a column temperature of 40 °C. A 23-min gradient elution was employed with eluent A (95/5 H_2_O/MeOH + 2 mM NH_4_Ac) and eluent B (95/5 MeOH/H_2_O + 2 mM NH_4_Ac). The injection volume was 5 µL. The gradient started at 15% B followed by a linear increase to 100% B within 10 min and kept isocratic conditions for 5 min. A post time of 8 min was used to equilibrate the column for initial conditions (15% B). The ionization source was operated separately in negative and positive ionization mode (ESI − /ESI +) due to the presence of anionic, cationic, and zwitterionic substances. For higher MS^2^ coverage, QTOF data acquisition was performed in an iterative data-dependent MS^2^ mode (ddMS^2^), injecting each sample five times with a dynamic exclusion list to prevent MS^2^ triggering of precursors already selected for MS^2^ in previous injections of the sample. The threshold for MS^2^ triggering was 1000 counts and after recording 3 MS^2^ spectra, the respective precursor m/z was excluded for the next 0.5 min. The acquisition rate was 3 spectra/s in both MS^1^ and MS^2^, and a narrow isolation window was chosen (1.3 m/z). A linear m/z-dependent collision energy (CE) was applied for collision-induced dissociation according to the following equation: CE (m/z) = 3 $\frac{m/z}{100}$ + 15 eV. For prioritized substances with unsatisfactory MS^2^ spectra, additional targeted MS^2^ measurements were performed with fixed CE (10, 20, 40 eV) and an acquisition rate of 2 spectra/s. Samples were run together with an extraction blank.

### Data evaluation

#### Identification of target analytes

Target analytes (confidence level 1) were identified by matching the accurate mass, retention time (RT) and MS^2^ spectra with an analytical reference standard. For target analysis, 51 PFAS reference standards including 20 PFAAs and 5 AFFF substances were available (ESM 1.A, Table S1).

#### Feature prioritization

For prioritization of features and identification of potential PFAS, the in-house developed open-source, Python-based software PF∆*Screen* was used [45]. Feature finding was performed with a mass error of 10 ppm and an intensity threshold of 1000 counts for the peak finding algorithm. An isotope model for small molecules was applied for isotope clustering. For alignment of MS^2^ fragments to their respective MS^1^ peaks, a mass tolerance of 5 mDa and a RT tolerance of 12 s (± 6 s) from the MS^1^ peak was used. Features were removed in the blank correction procedure when a feature in the sample aligned with a feature in the extraction blank within a mass tolerance of 2 mDa and a RT tolerance of 6 s. The applied values were chosen according to the peak width of the signals. Furthermore, features were only considered if the peak area was 5 times higher in the sample than in the extraction blank. For prioritization of possible PFAS features, the output feature table of PF∆*Screen* was sliced, keeping only features with an m/C value > 23 and a RT > 4 min. The cut-off of the m/C value was chosen according to the typical m/C value range of PFAS (m/C = 30–60) [43], depending on the degree of fluorination with highly fluorinated PFAS such as PFAAs exhibiting higher m/C values (e.g., 46 for PFOA and 62.5 for PFOS) than less fluorinated PFAS such as 4:4 fluorotelomer betaine (4:4 FTB) with m/C = 31.5. Since PF∆*Screen* calculates the m/C values based on the abundance of the M + 1 isotope, which can lead to errors in cases of peak saturation, peak overlap, or too low abundance of the M + 1 peak, the threshold was set slightly lower to ensure that all relevant PFAS are detected. This conservative value still proved viable as more than 90% of the features were removed from the data with a cut-off at a value of m/C = 23 alone (ESM 1.C, Fig. S2a). With the applied reversed-phase chromatography method, highly polar substances typically elute in the first 4 min of the method, while the PFAS of interest are moderately polar to non-polar substances that elute only after 4 min.

Further data reduction was achieved with the help of the extracted ion chromatogram (EIC) correlator in the “Raw Data Visualization” tool within PF∆*Screen* [45] which correlates EICs at a certain RT-window with an individually adjustable coefficient of determination *R*^2^ (*R*^2^ > 0.97). Several substances in the soil samples formed various in-source fragments and/or adducts which appeared in the output table of PF∆*Screen* as separate compounds but could be assigned to a respective molecular ion ([M-H]^−^, [M-2H]^−^, [M + H]^+^, or [M]^+^) with the help of the EIC correlator.

#### Identification of suspect- and non-target features

After prioritization, a PFAS suspect screening was conducted with PF∆*Screen* [45] for which the suspect list from the National Institute of Standards and Technology (NIST) [46] together with an in-house prepared suspect list was used. Additional to the suspect screening with PF∆*Screen*, the literature was manually searched for m/z values of relevant AFFF-related PFAS. This resulted in the inclusion of some features that were not yet included in the PF∆*Screen* output table. These manually detected features of interest often showed relatively low abundances, which is probably why they were not detected by the feature detection algorithm of PF∆*Screen* as their intensities were below the selected intensity threshold.

Prioritized features (m/C > 23, RT > 4 min) that did not produce a suspect hit in the PF∆*Screen* output table, as well as features that were manually prioritized by literature research but for which no structure was proposed in the literature, were subject to a non-target screening workflow.

The identification process of PFAS started by checking for presence of all possible molecular ions in both ionization modes (ESI − /ESI +) and determining if a suspected PFAS or feature of interest was anionic, cationic, or zwitterionic. Then the presence of further homologues belonging to the feature was checked by searching for repeating CF_2_ units with the help of the “Raw Data Visualization” tool in PF∆S*creen.* Homologues which show mass spacing of ∆m = 49.9968 (CF_2_ repeating unit) indicate an ECF-based structure, while homologues with a mass difference of ∆m = 99.9936 (C_2_F_4_ repeating unit) indicate a fluorotelomer (FT)-derived homologous series [44]. The peak shape of a feature can provide further information about the manufacturing process of the supposed PFAS. A non-baseline separated split peak usually indicates the presence of differently branched and/or linear isomers which suggests an ECF-based structure.

Isotopic patterns were checked against the proposed structure in case of a suspect hit and the structure either was matched or discarded. If no structure was proposed, isotopic patterns and exact mass were used to determine possible molecular formulae with *MSTools* [47] and *ChemCalc* [48]. The isotopic pattern match was calculated according to Eq. 1:

1 $$\text{Isotopic pattern match}=\left({1}-\left|\left({\text{I}}_{\text{M+1}}^{\text{theoretical}}\right)-\left({\text{I}}_{\text{M+1}}^{\text{measured}}\right)\right|\right)\bullet \left({1}-\left|\left({\text{I}}_{\text{M+2}}^{\text{theoretical}}\right)-\left({\text{I}}_{\text{M+2}}^{\text{measured}}\right)\right|\right)$$

where I is the peak area of the theoretical and measured M + 1 and M + 2 peak, normalized to the M peak (similar to Pluskal et al. [49]).

If MS^2^ information was available for suspect hits, MS^2^ spectra were investigated manually for the occurrence of matching fragments to the proposed structure. Features with available MS^2^ information and no suspect hit were investigated for typical PFAS fragments and fragment differences. The software tool *MetFrag* [50] was also utilized to verify potential chemical structures. In negative ionization mode, FT-based PFAS often show losses of HF (∆m = 20.0062). Fragments of the perfluoroalkyl chain (C_n_F_2n+1_)^−^ with fragment mass differences of 49.9968 (CF_2_) indicate a PFAS. Frequently occurring diagnostic fragments for AFFF PFAS in negative mode such as [SO_3_]^−^, [HO_3_S]^−^, [SO_3_F]^−^, [SO_2_F]^−^, [CH_3_O_3_S]^−^, [NO_2_S]^−^, [H_2_NO_2_S]^−^, and [C_5_H_11_N_2_O_2_S]^−^ can further provide information about functional groups and PFAS structures.

In positive ionization mode, fragments of the per- and polyfluoroalkyl chain only and their characteristic mass fragment differences do not occur, but informative positive ionizing fragments of the non-fluorinated molecule part can give valuable information on molecule structure. Frequent diagnostic fragments of positive ionizing AFFF PFAS are for example [C_4_H_11_N]^.+^, [C_5_H_11_N]^.+^, [C_4_H_8_N]^+^, [C_5_H_14_N_2_]^.+^, [C_4_H_10_NO_2_]^+^, [C_4_H_8_NO_2_]^+^, and [C_5_H_12_NO_2_]^+^ and can indicate for example the presence of a betaine or trimethylamine moiety in a molecule [23].

#### Classification into confidence levels

To classify the identified substances into confidence levels, a scheme adapted from Charbonnet et al. [51] is introduced here (ESM 1.D, Table S2). Since PFAS are rare in MS^2^ libraries, the criterion for library MS^2^ matching was discarded. Instead, the m/C value was included as a criterion (m/C > 25) and experimental data from the literature (e.g., provided MS^2^ spectra in peer-reviewed publications) can also be considered in determining the confidence level of a feature similar to the initially proposed confidence level for small molecules by Schymanski et al. [52].

## Results and discussion

### Terminology

Perfluorohexane sulfonamide propyl dimethylamine (PFHxSAm-Pr-DiMeAm), the most predominant PFAS species in AFFF apart from PFHxS and PFOS [53], and its fluorotelomer analogue 6:2 fluorotelomer sulfonamide propyl dimethylamine (6:2 FTSAm-Pr-DiMeAm) have both received multiple names in literature, which are not necessarily consistent or describe the structure unambiguously (PFHxSaAm [22], AmPr-FHxSA [33], N-AP-FHxSA [54], PFHxSAm [16], and PFHxSAA [14] for PFHxSAm-Pr-DiMeAm (Table 3, row 5) and 6:2 FtSaAm [22], N-AP-6:2 FASA [23], 6:2 FTSAPr-DiMeAn [33], 6:2 FTAA [55], and 6:2 FTA [39] for 6:2 FTSAm-Pr-DiMeAm (Table 3, row 7)). In an effort to counteract this development, the new terminology for AFFF PFAS was established and applied to all identified PFAS. The naming approach incorporates the terminology conventions of Buck et al. [4] and aligns with multiple designations found in the literature, ensuring a consistent terminology where the structure can be derived from the acronym. All rules and acronyms for specific moieties are specified in Table 1. In short:

- The acronyms for the different parts of the chemical structure are assembled stepwise, starting from the per-/polyfluoroalkyl moiety on the left and progressing to the right. Each subsequent moiety is added sequentially and separated by hyphens (e.g., 6:2 FTSAm-Pr-B, 6:2 fluorotelomer sulfonamide propyl betaine, Table 1).
- The acronyms for side chains and substitutions are placed directly in front of the respective acronym to which they are attached. The positions of side chains, as well as keto, ether, unsaturated, H-substituted, and OH-substituted moieties, are indicated by numbers in parentheses (e.g., (6)U-(5)E-PFHpS, Table 1).
- If the main chain branches at the sulfonamide nitrogen into chains X and Y, this is denoted as *N*-X-*N*-Y (e.g., PFHxSAm-*N*-Et-*N*-EtA, Table 1).

A table with all acronyms of subclasses/compounds with an example structure, formula, International Chemical Identifier (InChI), and alternative names in literature can be found in ESM 2.

**Table 1:**
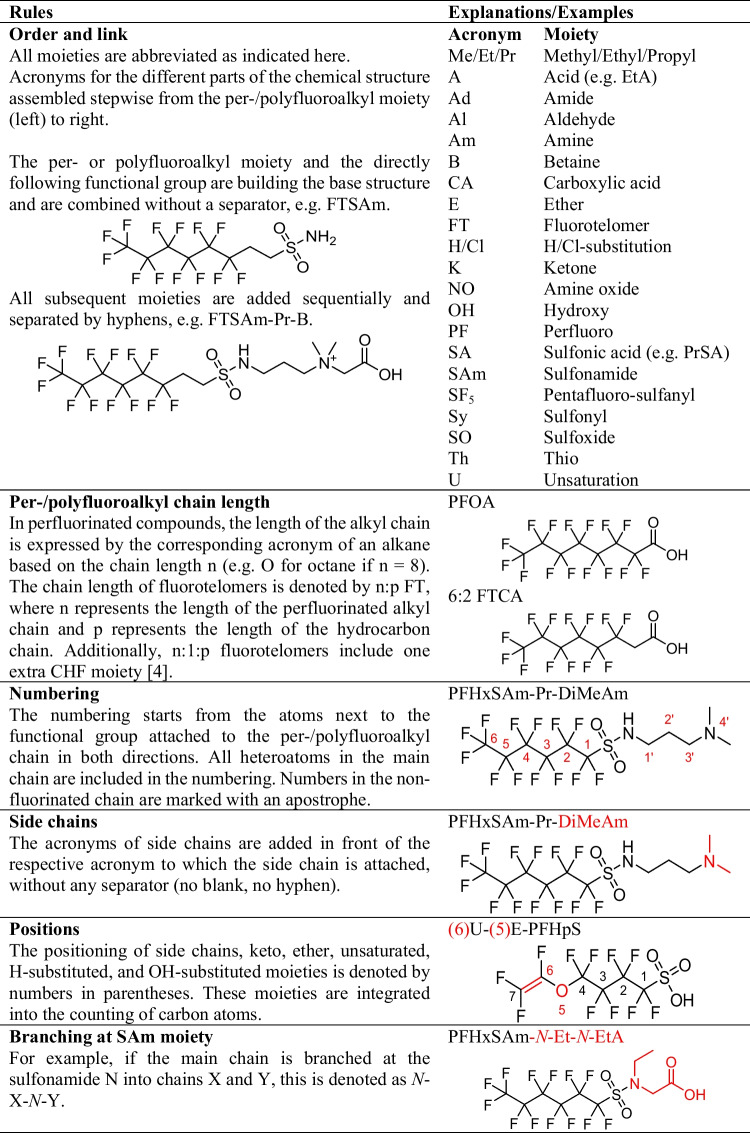
Rules for the new systematic terminology with acronyms of specific moieties and examples

### Choice of extraction method

We investigated the performance of four different extraction methods: extraction employing neutral and basic methanol (MeOH, MeOH + 0.1 M NH_4_OH, MeOH + 0.4 M NH_4_Ac) as extraction solvents, along with the method described by Nickerson et al. [33] which employs the combination of acidic and basic extraction solvents. The extraction efficiencies were compared for 19 anionic, zwitterionic, and cationic PFAS (ESM 1.E, Fig. S3). For anionic PFAS, all of the enhanced extraction methods only showed a small improvement or even a decrease in extraction efficiency. For cationic and zwitterionic PFAS, MeOH + 0.1 M NH_4_OH performed worst for 7 out of 9 zwitterionic and cationic substances and was therefore not used. Therefore, in a next step, we conducted a three-step sequential extraction with MeOH + 0.4 M NH_4_Ac and compared it to the method of Nickerson et al. [33] (ESM 1.E, Fig. S4). Extraction efficiencies were similar for both methods, but the extraction with MeOH + 0.4 M NH_4_Ac showed slightly better recoveries for 7 out of 19 substances. Due to the reduced workload compared to the method from Nickerson et al. [33], the three-step sequential extraction with MeOH + 0.4 M NH_4_Ac as extraction solvent was chosen as the final extraction method. No contamination could be detected in the extraction blank.

### PFAS prioritization

**Fig. 1:**
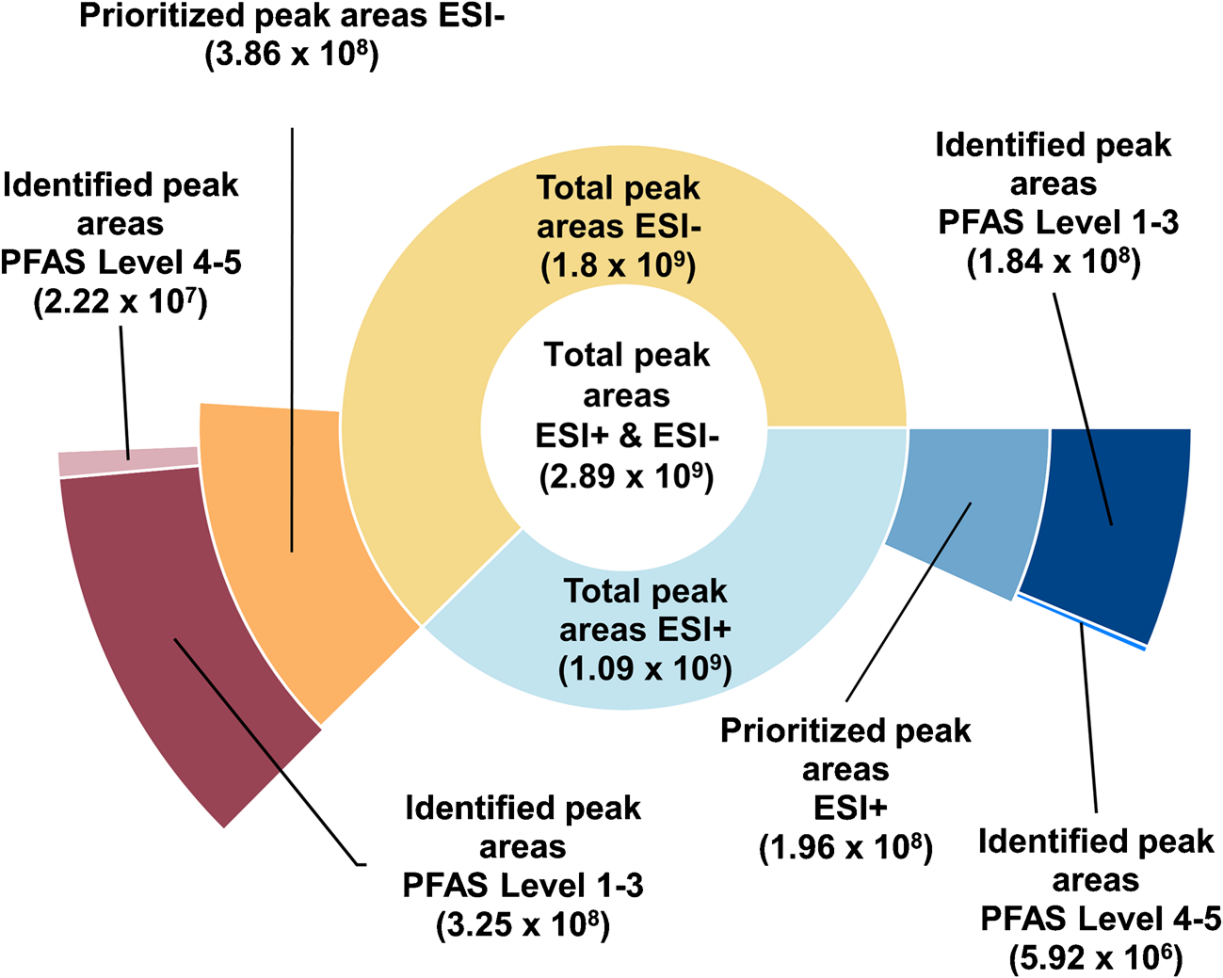
Cumulative peak areas of all features from the PF∆S*creen* output tables (inner sections), peak areas of prioritized features filtered for RT > 4 min and m/C > 23 (middle sections), and peak areas of features identified as PFAS in negative and positive ESI mode (outer sections), respectively. Peak areas of in-source fragments and adducts were first combined with the respective parent ion

The extract of soil sample *S1* in the upper horizon (0–0.5 m) was analyzed by LC-HRMS in ESI + and ESI − and data evaluation was performed with the open-source tool PF∆*Screen* [45]. The output tables of PF∆*Screen* for soil yielded a total of 2262 and 1921 non-target features in ESI − and ESI + , respectively (after componentization of isotope clusters). Prioritization of features was reached by cutting off the output tables of PF∆S*creen* and only keeping features with m/C > 23 and RT > 4 min. The m/C threshold was chosen to separate hydrocarbon-dominated substances in the sample matrix from highly fluorinated PFAS [43] and the RT threshold to separate early eluting polar compounds from the moderately polar to non-polar PFAS. This led to a significant reduction in total peak areas of 79% for ESI − and 82% for ESI + , corresponding to a reduction of total number of features of 95%. Ninety-two and 94% of the respective prioritized peak areas (5% of total features) could be identified as PFAS (levels 1–3, 124 features) in ESI − and ESI + , respectively. An additional 6% and 3% of the prioritized peak areas in ESI − and ESI + , respectively, were classified as potential PFAS (levels 4 and 5, 114 features in total, Fig. 1).

Prioritizing via the m/C and RT dimension led to significant feature reduction (Fig. S2a) and major fractions of the prioritized peak areas could be identified as PFAS. PFAS prioritization via the MD/C-m/C approach was proven to be effective before [43] and the performance of the prioritization workflow applied in this work (RT > 4 min and m/C < 23) was validated by application to a standard mix (ESM 1.C). A histogram of the m/C values of all PFAS of the NIST suspect list [46] (ESM 1.C, Fig. S2b) reveals that only a small fraction (0.9%) of specific PFAS with low fluorine percentage show a m/C below 23, indicating a low probability to exclude PFAS (false negatives) via this prioritization technique. We did not consider PFAAs with chain length shorter than perfluoropentanoic acid (PFPeA) and perfluoropropane sulfonic acid (PFPrS) and similar highly polar PFAS due to RT cut-off at 4 min. Further limitations result from the choice of extraction and ionization methods.

### Identified PFAS in soil and groundwater samples

**Fig. 2:**
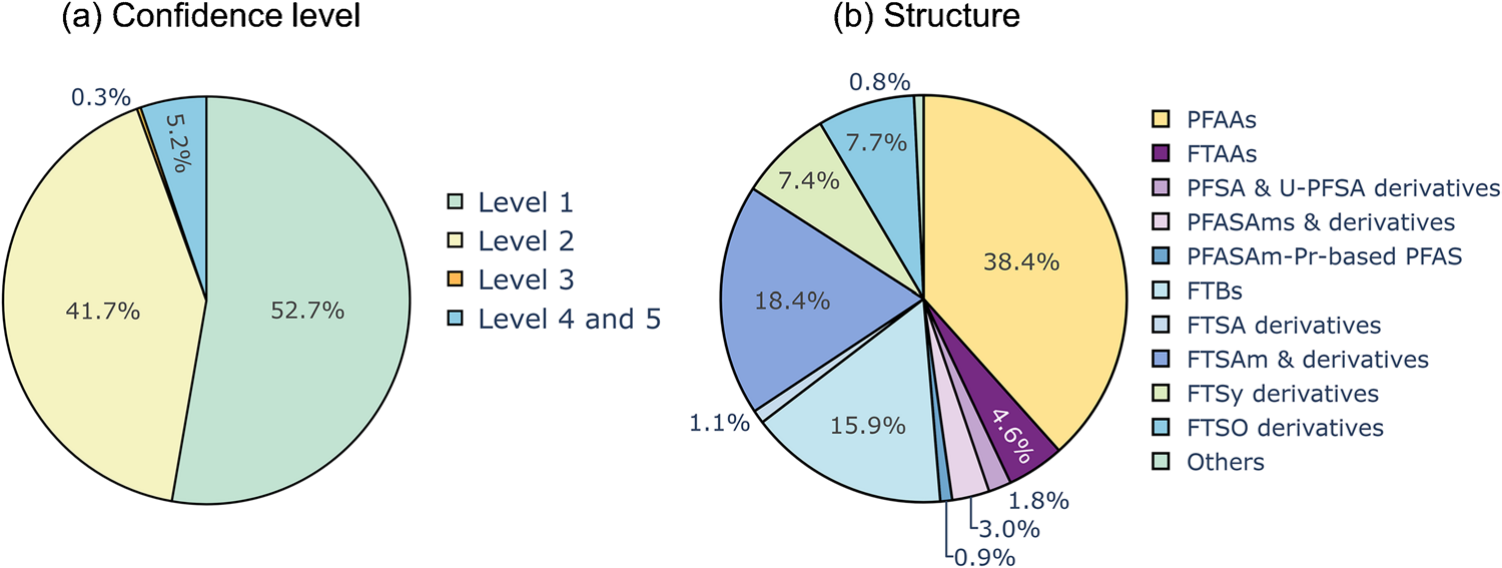
Distribution of peak areas in soil across the identification levels (**a**) and for different PFAS subclasses (**b**; levels 1–3; see also Tables 2 and 3)

Suspect hits and non-target features were identified according to the identification workflow described above. In total, 124 features of 42 subclasses were identified as PFAS in soil (levels 1–3, Tables 2 and 3). Ten of the 42 identified subclasses were identified as level 1, 28 at level 2, and 4 at level 3, corresponding to 52.7%, 41.7%, and 0.3% of the total peak area, respectively (Fig. 2a). Twelve of the 42 identified subclasses in soil were also present in the groundwater sample (25 individual compounds, Tables 2 and 3).

At level 1, substances in soil were confirmed with corresponding reference standards (target compounds). For level 2 substances, a possible structure is proposed that is supported by meaningful MS^2^ fragments. At level 3, one molecular formula is proposed but various chemical structures can be possible due to the lack of meaningful MS^2^ fragments. The poor quality of MS^2^ spectra often results from low peak intensity of the respective substances. All substances identified at levels 1, 2, and 3 are included with (possible) structure, name, and additional information in ESM 2. For level 4 substances, a molecular formula is proposed according to accurate mass and isotopic pattern match and level 5 substances are features of interest that are potentially PFAS due to a suitable m/C value and the occurrence of homologous series. In total, 114 individual compounds were categorized into levels 4 and 5, including 17 homologous series. Level 4 and level 5 substances together contribute to 5.2% to the total peak area of all level 1–5 substances in the soil (for more details on the classification of confidence levels and fragmentation evidence, refer to ESM 1.D and G; for more detailed information on all identified substances, explanations of all acronyms, molecular formula, ionization polarity, alternative names in the literature, additional information from the literature, and criteria used for assigning the single substances to specific confidence levels, refer to the ESM 2). Note: In some cases, corresponding homologues within one subclass were assigned at different confidence levels. A subclass is described in the following section at the level of the homologue with the highest confidence level.

#### Newly identified substances and substances identified for the first time in soil

One PFAS subclass with two homologues (n:2/m:2 FTSAm dimers) was newly identified in the soil samples (red font in Table 2). To the best of our knowledge, 9 further PFAS subclasses were detected in soil for the first time (three fluorotelomer betaines, three fluorotelomer sulfonamides, one fluorotelomer sulfone, and two fluorotelomer sulfonic acids, Table 2). All subclasses discussed in this section were identified at level 2, except for n:1:2 FTB which was identified at level 3.

##### Fluorotelomer sulfonamide dimer (n:2/m:2 FTSAm dimer, Table 2, row 1)

**Fig. 3:**
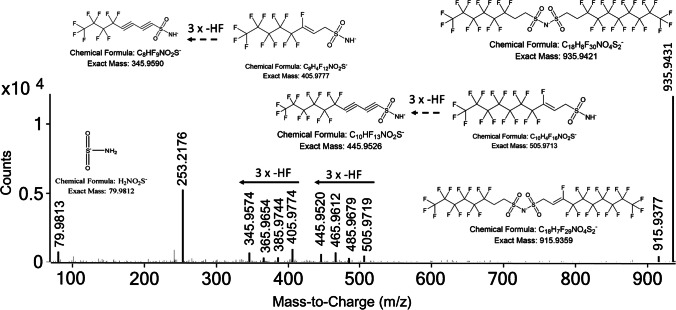
Annotated MS^2^ fragmentation spectrum of 6:2/8:2 FTSAm dimer (m/z = 935.9431, RT = 10.2 min) in ESI − at a collision energy of 43 eV

Two homologues of the newly identified n:2/m:2 fluorotelomer sulfonamide dimer (n:2/m:2 FTSAm dimer) were detected (6:2/6:2 at 9.36 min, exact mass m/z 835.9485 and 6:2/8:2 at 10.38 min, m/z 935.9421) which can be considered as fluorotelomer derivatives of the anion bistriflimide (bis(trifluoromethane)sulfonimide, also known as NTf2). Yukioka et al. [56] and Luo et al. [37] already detected this compound as unknown. Our MS^2^ data strongly support the proposed structures with 6 and 10 meaningful fragments, respectively (level 2, Fig. 3, S5b, and S5c). Both compounds show the diagnostic fragment [SO_2_NH_2_]^−^ (exact mass m/z 79.9812), indicating the presence of a sulfonamide group which is also detected for other sulfonamide-based PFAS like 6:2 FTSAm (Fig. S36b), [M-H-HF]- ions (at exact mass m/z 815.9423 and m/z 915.9539) indicative for fluorotelomer-based PFAS and fragments resulting from a cleavage of one fluorotelomer side chain (m/z 405.9777 and m/z 505.9713) combined with further three consecutive HF losses (exact mass m/z 405.9777 → 385.9714 → 365.9645 → 345.9652, Fig. 3 and S5c). In the case of the asymmetrically substituted 6:2/8:2 FTSAm dimer, both series of HF losses occur from the 6:2 FTSAm fragment (exact mass m/z 405.9777) and the 8:2 FTSAm fragment (exact mass m/z 505.9713). Formation of the dimer from n:2 FTSAm as ionization artifact can be ruled out due to chromatographic separation of n:2/m:2 FTSAm dimers compared to the corresponding fluorotelomer sulfonamides which elute much earlier (RT 7.77 min and 8.88 min, respectively; Fig. S36a and S5a).

##### Fluorotelomer sulfonamide–based PFAS (n:2 FTSAm-based subclasses, Table 2, row 1)

The three FTSAm-based subclasses n:2 fluorotelomer sulfonamide *N*-methyl *N*-propanoic acid (6:2 FTSAm-*N*-Me-*N*-PrA), n:2 fluorotelomer sulfonamide unsaturated propyl dimethylamine (6:2 FTSAm-U-Pr-DiMeAm), and n:2 fluorotelomer sulfonamide propanoic acid (6:2 FTSAm-PrA) were identified for the first time in soil. Various series of HF losses support a fluorotelomer structure for 6:2 FTSAm-*N*-Me-*N*-PrA and diagnostic fragments such as [SO_2_NH_2_]^−^ and [C_4_H_8_NO_4_S]^−^ (exact mass m/z 166.0180) are indicative for the functional groups and the non-fluorinated part of the molecules (Fig. S6c). 14 MS^2^ fragments could be matched to the proposed structures, of which 11 fragments could be matched with those reported by Fang et al. [57]. For 6:2 FTSAm-U-Pr-DiMeAm, three MS^2^ fragments could be matched to the structure including the fragment [C_10_H_9_F_13_NO_2_S]^+^ (due to the loss of the terminal C_3_H_7_N moiety) and fragments of the quaternary ammonium moiety (Fig. S7b), all in accordance with the fragments observed by Fang et al. [57]. For 6:2 FTSAm-PrA (Fig. S8b), several fragments could be assigned to the polyfluorinated carbon moiety ([C_3_F_7_]^−^, [C_4_F_9_]^−^, [C_8_F_9_]^−^) and the functional group ([NSO_2_]^−^, [FSO_2_]^−^, [C_3_H_6_NO_4_S]^−^) and loss of HF. The *n* = 6 homologues of all three subclasses were detected as biotransformation products of 6:2 fluorotelomer sulfonamide propyl dimethylamine oxide (6:2 FTSAm-Pr-DiMeNO) in aerobic sludge by Fang et al. [57]. In addition, 6:2 FTSAm-PrA was also observed as an intermediate in aerobic biodegradation of 6:2 fluorotelomer sulfonamide propyl dimethylamine (6:2 FTSAm-Pr-DiMeAm) in sludge [55].

##### Fluorotelomer betaines (n:2 FTB, n:1:3 FTB, n:4 FTB, Table 2, row 2)

The diagnostic fragments [C_4_H_10_NO_2_]^+^ (exact mass m/z 104.0706) and [C_5_H_12_NO_2_]^+^ (exact mass m/z 118.0831) are indicative for the FTB class (Fig. S9b) and were detected for at least one homologue of the two FTB subclasses (n:2 and n:4 FTB; Fig. S9b,c and S10b). For n:1:3 FTB, only m/z 118.0863 was detected (Fig. S11b). In general, the MS^2^ spectra of FTBs are rather poor in fragments due to the fixed positive charge at the quaternary ammonium moiety. Therefore, correct and accurate mass match and isotope patterns are crucial parameters in this case. All three FTB subclasses were previously identified in Ansulite AFFF in lower abundance compared to n:1:2 and n:3 FTBs [17]. This corresponds very well with the results of the homologue analysis. Whereas less homologues occurred for n:2 FTB (*n* = 6, 8, 10, 12), n:4 FTB (*n* = 6, 8, 10), and n:1:3 FTB (*n* = 6, 8, 10), an extended range was found for n:3 FTB (*n* = 5, 7, 9, 11, 13) and n:1:2 FTB (*n* = 5, 7, 9, 11, 13, 15).

##### Fluorotelomer sulfonic acids (K-n:2 FTSA and U-n:2 FTSA, Table 2, row 3)

Ketone-n:2 fluorotelomer sulfonic acids (K-n:2 FTSAs) and unsaturated-n:2 FTSAs (U-n:2-FTSAs) both show fragments of the unsaturated fluorinated carbon chain as well as the diagnostic fragment [SO_3_H]^−^ (exact mass m/z 80.9652) which is characteristic for the sulfonic acid moiety in the vicinity of a hydrogen atom. For both subclasses, at least three fragments (e.g., [C_2_F_5_]^−^, [C_2_F_7_]^−^, [C_7_F_9_]^−^, [C_7_F_11_]^−^) could be matched with the proposed structures (Fig. S12b, S13b). All MS^2^ fragments of both subclasses were also observed by Fang et al. [57] who detected both, K-6:2 FTSA and U-6:2 FTSA as biotransformation products of 6:2 FTSA in aerobic sludge. U-n:2 FTSAs resulting from in-source fragmentation (loss of water) of K-n:2 FTSAs could be excluded due to chromatographic separation of the two subclasses (e.g., U-6:2 FTSA at 7.03 min; K-6:2-FTSA at 6.92 min, Fig. S12a and S13a).

##### Fluorotelomer sulfones (n:2 FTSy-(2′)OHPr-TriMeAm, Table 2, row 4)

n:2 fluorotelomer sulfone (2′)-propanol trimethylamine (n:2 FTSy-(2′)OHPr-TriMeAm, Fig. S14b,c) was identified at level 2 according to matches of five meaningful MS^2^ fragments for *n* = 6 and *n* = 8 homologues. The fragments m/z 469.0138 and m/z 412.9875 are formed by losses of the terminal moieties C_3_H_9_N and further C_3_H_4_O from the molecular ion [M-H]^+^ ([C_14_H_19_F_13_NO_3_S]^+^). Further MS fragments are ions from the terminal end of the molecule, [C_6_H_16_NO_3_S]^+^ (exact mass m/z 182.0845), [C_3_H_7_O_3_S]^+^ (exact mass m/z 123.0110), and [C_5_H_12_NO]^+^ (exact mass m/z 102.0913). This subclass was already identified in various AFFF formulations [17, 21, 36, 58], but to the best of our knowledge never in soil.

**Table 2:**
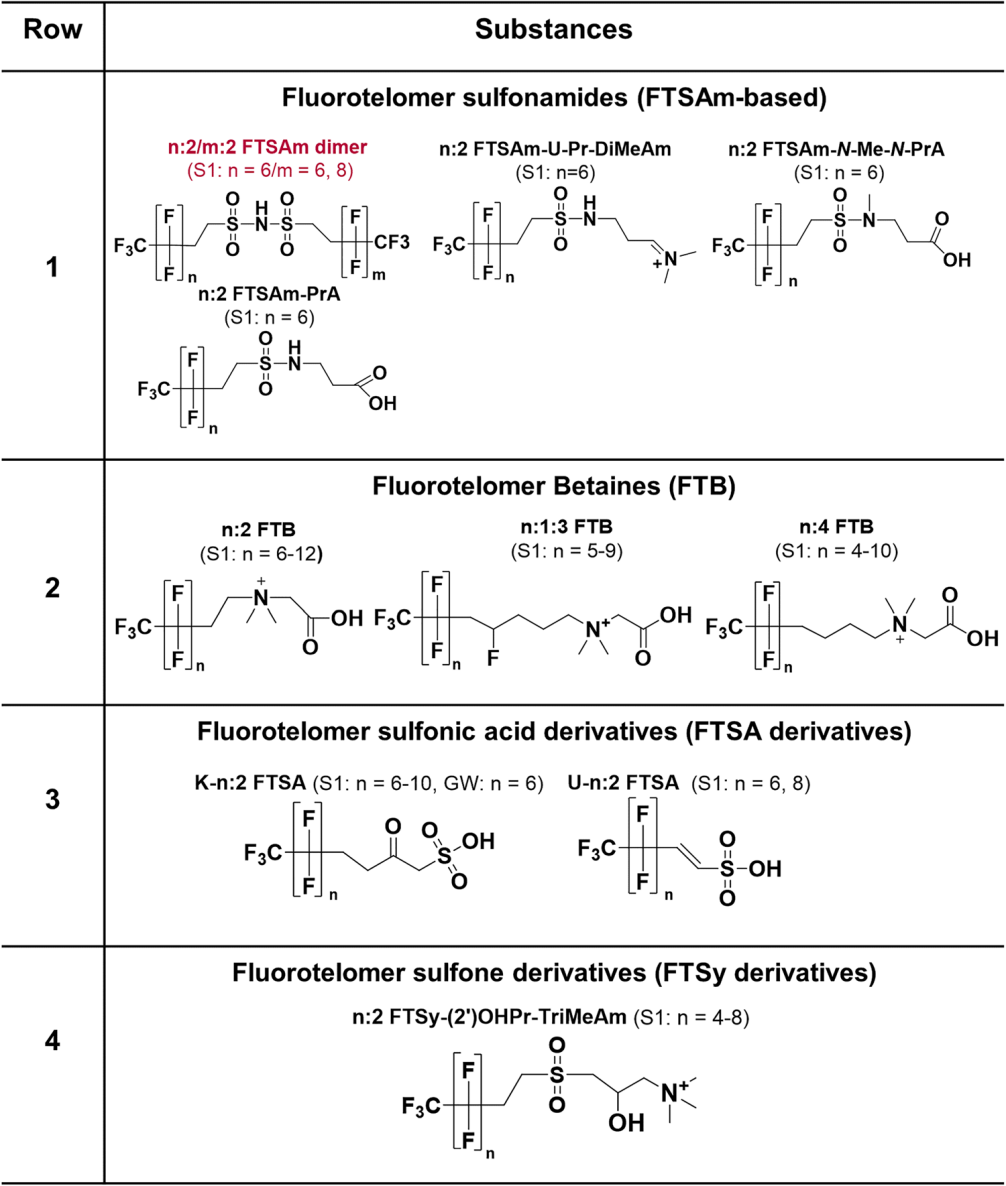
Summary of newly identified PFAS (red font) and newly in soil identified PFAS (black font) at the field site in Reilingen (soil, S1 and groundwater, GW). Identified homologues are denoted in brackets. Note that some homologues might have a lower confidence level (ESM 2)

#### Further PFAS in soil and groundwater

Fragmentation evidence for all identified level 2 and level 3 substances can be obtained from ESM 1.G.

##### PFCA, PFSA, FTCA, and FTSA (Table 3, row 1)

PFAAs such as PFCAs and PFSAs as well as n:3 fluorotelomer carboxylic acids (n:3 FTCAs) and n:2 FTSAs were detected at level 1. The group of PFAAs constitutes the largest portion of the contamination based on peak area, contributing to 38.4% of the total peak area of all level 1–3 substances (Fig. 2b). This is not surprising since both PFCAs and PFSAs were mainly used in various AFFFs from 3 M and Angus [18, 21, 22]. FTCAs and FTSAs comprise only 4.6% of the total peak area of all level 1–3 substances, although n:2 FTSAs are also known to be found in various AFFF formulations [53, 58].

##### PFSA- and U-PFSA-derivatives (Table 3; rows 2, 3, and 10)

PFSA derivatives include Cl-substituted PFSAs** (**Cl-PFSAs), pentafluorosulfanyl PFSAs (SF_5_-PFSAs), H-substituted PFSAs (H-PFSAs), ketone PFSAs (K-PFSAs)/unsaturated ether PFSAs (U-E-PFSAs), and perfluoro sulfinic acids (PFASyAs). Unsaturated PFSA (U-PFSA) derivatives include U-PFSAs and pentafluorosulfanyl unsaturated PFSAs (SF_5_-U-PFSAs). All above listed subclasses were determined at level 2 besides K-PFSAs/U-E-PFSAs which were identified at level 3 due to the lack of meaningful MS^2^ spectra. PFSA and U-PFSA derivatives only represent minor fractions of the total peak area of all identified level 1–3 substances (1.8%, Fig. 2b). All PFSA derivatives but SF_5_-U-PFSAs have been detected in 3 M AFFF [23].

##### PFASAm-based subclasses (Table 3; rows 4, 5, and 10)

Perfluoroalkyl sulfonamide–based PFAS (PFASAms) and perfluoroalkyl sulfonamides propyl dimethylamines (PFASAm-Pr-DiMeAms) were identified at level 1. PFASAm-Pr-DiMeAms is one of the most predominant compounds in AFFF [53]. At level 2, perfluoroalkane sulfonamide ethanoic acids (PFASAm-EtAs), perfluoroalkane sulfonamide propyl sulfonic acids (PFASAm-PrSAs), perfluoroalkane sulfonamide propyl betaines (PFASAm-Pr-Bs), and perfluoroalkane sulfonamide propyl trimethylamines (PFASAm-Pr-TriMeAms) were identified. The PFASAm-based perfluoroalkane sulfonamide methyl (PFASAm-Mes) and perfluoroalkane sulfonamide *N*-propyl sulfonic acid *N*-propyl dimethylamine (PFASAm-*N*-PrSA-*N*-Pr-DiMeAm, Table 3, row 10) were identified at level 3. An isomer of PFASAm-*N*-PrSA-*N*-Pr-DiMeAm was previously detected in AFFF from 3 M (class 2 (*N*-SPAmP-FASAs) according to Barzen-Hanson et al. [23]). In this study, MS^2^ data (m/z 519.9564, [C_9_H_7_F_13_NO_5_S_2_]^−^, Fig. S33c) suggest branching at the first nitrogen moiety, which still needs to be validated by further analyses. PFASAm and PFASAm-Pr-based substances together only contribute to 1.2% of the total peak area (Fig. 2b).

##### FTBs (Table 3, row 6)

FTBs represent a significant portion of the total peak area (15.9%, Fig. 2b) and include five subclasses in total of which three subclasses were identified for the first time in soil (discussion in previous section). The remaining two subclasses were assigned level 1: n:1:2 FTBs and n:3 FTBs. Both have been detected abundantly in various AFFF formulations [16, 17, 21, 22, 36] and therefore contribute to a significant portion to the total FTB peak area (97.7%). In contrast, n:2, n:1:3, and n:4 FTBs contribute only to a minor fraction and have been detected with less frequency in AFFF [17, 23].

##### n:2 FTSAm derivatives (Table 3, row 7)

Identified PFAS, sharing a common n:2 FTSAm-based structure, represent the second largest group of substances based on peak area after PFAAs (18.4%, Fig. 2b) and include eight substance subclasses of which two were identified at level 1: the two, well-known AFFF compounds n:2 fluorotelomer sulfonamide propyl betaines (n:2 FTSAm-Pr-Bs) and n:2 FTSAm-Pr-DiMeNOs. All other FTSAm-derivatives were identified at level 2 of which n:2-FTSAm-*N*-Me-*N*-PrAs n:2 FTSAm-PrAs and n:2 FTSAm-U-Pr-DiMeAms (Table 2, row 1) were detected for the first time in soil in this study, to the best of our knowledge (discussion in previous section). n:2 FTSAm-Pr-MeAms and n:2 FTSAm-Pr-DiMeAms both were detected in AFFF and AFFF-impacted environment before [17, 36, 37] and are possible TPs or synthetic intermediates of n:2 FTSAm-Pr-Bs and n:2 FTSAm-Pr-DiMeNOs [17, 25, 57].

##### n:2 FTSy-based PFAS (Table 3, row 8)

Two further PFAS subclasses sharing a fluorotelomer sulfonyl (FTSy)-based structure were detected and identified at level 2. All FTSy-based PFAS together, including the previously discussed 6:2 FTSy-(2′)OHPr-TriMeAm, which was detected for the first time in soil in this study contribute to 7.4% of the total peak area of all level 1–3 substances (Fig. 2b). 6:2 FTSy-(2′)OHPr-TriMeAm is among the top three most abundant substances in the soil based on peak area and contributes with 90% alone to the fraction of the FTSy-based substances of all identified level 1–3 substances, consequently contributing to 6.9% of the total peak area of all level 1–3 substances.

##### n:2 FTSO-based PFAS (Table 3, row 9)

Two PFAS subclasses sharing a fluorotelomer sulfoxide (FTSO)–based structure were detected at level 2. Though only consisting of two subclasses corresponding to 4 single substances, this group represents 7.7% of the total peak area of all level 1–3 substances. This is largely due to 6:2 fluorotelomer sulfoxide (2′)-propanol trimethylamine (6:2 FTSO-(2′)OHPr-TriMeAm) which contributes 98.6% alone to the peak area of the FTSO-based PFAS. Both subclasses have previously been detected in AFFF-impacted soil [39] and AFFF [21].

##### n:2 FTTh-based PFAS (Table 3, row 10)

n:2 fluorotelomer thio (2′)-propanol trimethylamine (n:2 FTTh-(2′)OHPr-TriMeAms) was identified at level 2 in soil. 6:2 FTTh-(2′)OHPr-TriMeAm contributes to 36.5% of the total peak areas of the “other” category (Fig. 2b) and is a well-known AFFF compound which was detected in various AFFF formulations before [16, 21, 22, 36].

##### n:2 FTSA derivatives (Table 3, row 10)

n:2 FTSA derivatives include hydroxy-n:2 FTSAs (OH-n:2 FTSAs), ketone-n:2 FTSAs (K-n:2 FTSAs), and unsaturated n:2 FTSAs (U-n:2 FTSAs). U-n:2 FTSAs and K-n:2 FTSAs both were detected in soil for the first time in this study (discussion in previous section). n:2 FTSA derivatives only represent a minor fraction of the total peak area of all identified level 1–3 substances (1.1%, Fig. 2b). As was shown that U-n:2 FTSAs and K-n:2 FTSAs can originate from n:2 FTSA biotransformation, OH-n:2 FTSA likely are either TPs or synthetic by-products of fluorotelomer-based AFFF substances such as n:2 FTSA as well [23, 57].

**Table 3:**
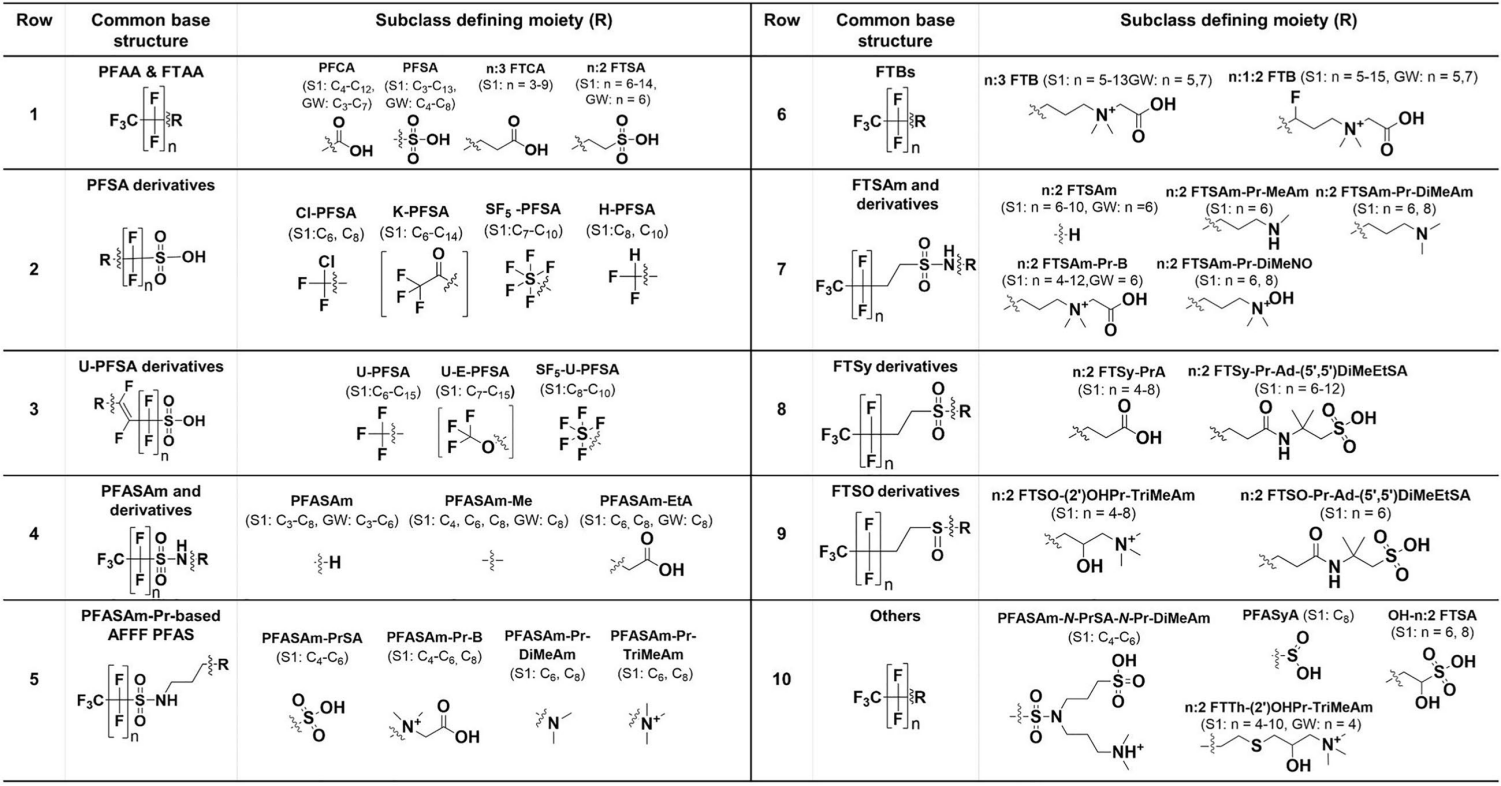
Summary of further identified PFAS at the Reilingen site in soil (S1) and groundwater (GW). The table shows all previously detected subclasses in soil where at least one homologue was assigned level 1–3. Note that for the sake of simplicity, only one positional isomer is depicted even if the correct position is unclear. K-PFSA/U-E-PFSA are in brackets indicating that both structures may be possible

### Characteristics of identified level 1–3 substances

**Fig. 4:**
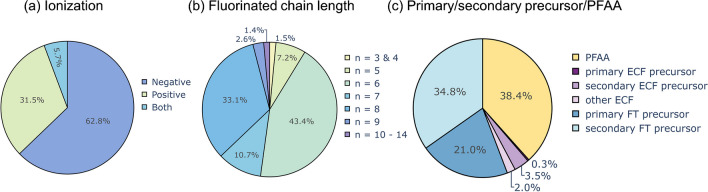
Distribution of peak areas of identified level 1–3 substances in soil characterized by the ionization mode (**a**), the perfluorinated chain length distribution (**b**), and the categories primary or secondary precursors, and PFAAs (**c**)

Negative ionizable PFAS are dominating the contamination pattern, representing 62.8% of the total peak area of all identified level 1–3 PFAS (Fig. 4a). This can be partly attributed to the high proportion of PFAAs present in the contaminated soil (38.4%, Figs. 2b and 4c), especially PFOS and PFHxS, which are the most abundant PFAAs based on peak area in soil. But also 6:2 FTSA and 6:2 FTSAm contribute to large parts of the fraction of negative ionizable compounds. Positive ionizable substances represent the second largest fraction comprising 31.5% of the total peak area. Substances dominating this group of substances are 5:1:2 and 7:1:2 FTB, 6:2 FTSO-(2′)OHPr-TriMeAm, and 6:2 FTSy-(2′)OHPr-TriMeAm. Zwitterionic substances make up 5.7% of the total peak area. The fraction of the zwitterionic substances is clearly dominated by 6:2 FTSAm-Pr-B, solely contributing to 80% of the total peak area of zwitterionic substances.

Considering the perfluorinated chain length distribution of the level 1–3 substances, PFAS with *n* = 6 represent the largest fraction of the total peak area (43.4%), followed by *n* = 8 (33.1%), *n* = 7 (10.7%), and *n* = 5 (7.2%) (Fig. 4b). PFAS with chain lengths of 3 and 4 and more than 10 fluorinated carbon atoms together only contribute to less than 3% of the total peak area. Compounds with shorter perfluorinated chain lengths than 3 (PFPrS) or 4 (PFPeA) have not been considered in this work. The *n* = 8 substances are dominated by PFOS (82.2%), followed by 8:2 FTSA and PFOSAm, all three together contributing 95% to the *n* = 8 fraction. The high abundance of *n* = 5 and *n* = 7 substances can be attributed to the n:1:2 and n:3 FTBs. The *n* = 6 and *n* = 8 fractions also include the highest numbers of single substances compared to all other chain lengths indicating that mainly C_6_ and C_8_ substances dominated the AFFFs applied for the firefighting activities. This is in compliance with most contemporary AFFF formulations, which are mainly based on C_6_-chemistry [53].

### Occurrence of identified PFAS in AFFF and the environment

Six out of the 14 most abundant PFAS in contemporary AFFF formulations [58] could be identified in the contaminated soil. These are all 6:2 FT-based PFAS and include 6:2 FTSA, 6:2 FTTh-Pr-(2′)OHPr-TriMeAm, two FTSO-based AFFF (6:2 FTSO-Pr-Ad-(5′,5′)DiMeEtSA, 6:2 FTSO-(2′)OHPr-TriMeAm), and two FTSAm-based AFFFs (6:2 FTSAm-Pr-DiMeNO, 6:2 FTSAm-Pr-B). The occurrence of FT-based and ECF-based precursors in soil at the Reilingen site clearly indicates that AFFF formulations from various manufacturers were used for firefighting activities. Despite the presence of numerous precursors, PFAAs represent a large part of the contamination (38.4%) and may originate either from the used AFFFs itself or have been formed in situ during transformation of precursors.

A recent review by Yan et al. [53] proposed a novel classification system for AFFF-derived PFAS precursors. In this system, primary precursors are defined as PFAS intended to be present in AFFF formulations as active agents. Secondary precursors encompass both transformation products (excluding PFAAs) and by-products resulting from primary precursors. Identified level 1–3 PFAS were assigned to the respective category (primary precursors/secondary precursor) based on literature review (Fig. 4c). While primary precursors (ECF + FT based) make up only about 21.3% of the total peak area, secondary precursors (ECF + FT) represent 38.3% of the total peak area similar to PFAAs accounting for 38.4% of the peak area. Highly abundant primary precursors identified in soil are for example 5:1:2 FTB, 7:1:2 FTB, 5:3 FTB, 7:3 FTB, 6:2 FTSAm-Pr-B, 6:2 FTTh-(2′)OHPr-TriMeAm, and PFOSAm-Pr-DiMeAm.

These findings are consistent with the review by Yan et al. [53] who determined that secondary precursors are more frequently detected in AFFF-contaminated areas, despite primary precursors being present in larger quantities in the original AFFF formulations [53]. This discrepancy can be explained by in situ biotransformation of primary precursors to secondary precursors [53]. At the field site in Reilingen, a signal of 6:2 fluorotelomer thio propyl amide dimethylethyl sulfonic acid (6:2 FTTh-Pr-Ad-(5′,5′)DiMeEtSA, structure in ESM 2), a widely used AFFF substance and primary precursor [17, 21, 22, 36, 53, 58], was detected but identified only at confidence level 4 due to low intensity and insufficient MS^2^ spectra. 6:2 FTTh-Pr-Ad-(5′,5′)DiMeEtSA is known to be readily biodegraded under environmental conditions and yields two known intermediate thio oxidation products, 6:2 FTSO-Pr-Ad-(5′,5′)DiMeEtSA and 6:2 FTSy-Pr-Ad-(5′,5′)DiMeEtSA [27, 59]. The latter two compounds both were detected in soil at level 2. Additionally, for n:2 FTSy-Pr-Ad-(5′,5′)DiMeEtSA, three more homologues were present in the soil (*n* = 8, 10, 12) while for n:2 FTTh-Pr-Ad-(5′,5′)DiMeEtSA and n:2 FTSO-Pr-Ad-(5′,5′)DiMeEtSA, only the *n* = 6 homologue could be detected. Similarly, n:2 FTTh-(2′)OHPr-TriMeAms also contain an oxidizable thio moiety, and its oxidation products (n:2 FTSO-(2′)OHPr-TriMeAms, n:2 FTSy-(2′)OHPr-TriMeAms) both were also present in soil. Both oxidation products have been detected in AFFF formulations [17, 21, 36]. While 6:2 FTSO-(2′)OHPr-TriMeAm is one of the most abundant PFAS in contemporary AFFF formulations [58], 6:2 FTSy-(2′)OHPr-TriMeAm mostly was detected at low concentrations, likely as impurity [21, 36]. However, in soil, 6:2 FTSy-(2′)OHPr-TriMeAm is one of the most abundant peaks and shows a 24 times higher peak area compared to the precursor compound (6:2 FTTh-(2′)OHPr-TriMeAm). This indicates its potential origin from in situ transformation of the precursor (6:2 FTTh-(2′)OHPr-TriMeAm) rather than from impurities in the AFFF formulation.

## Conclusions

The results of this study indicate that even after more than 15 years, sites contaminated with AFFF substances exhibit a wide variety of PFAS and their TPs and are therefore a long-term source for PFAAs due to biotransformation of precursors. To assess remediation measures and risk management, characterization of the PFAS pattern and its potential TPs has to be considered during planning, realization, and completion. A significant proportion of PFAS can easily be overlooked when applying targeted analytical methods, since analytical standards for AFFF PFAS, especially cationic ones, are lacking. NTS and suitable workflows for efficient data-prioritization are viable tools for the characterization of AFFF-contaminated sites. Data prioritization by the m/C dimension proofed to be rather efficient in this study. Nevertheless, still limited coverage of the chemical space has to be considered for NTS approaches due to a limited analytical window (choice of extraction method, ionization technique, chromatographic method, etc.). The lack of analytical standards also limits quantitative results. Therefore, robust semi-quantification approaches are needed to obtain quantitative information on the contaminant levels.

## Supplementary Information

Below is the link to the electronic supplementary material.

Supplementary file1 (PDF 6.29 MB)

Supplementary file2 (XLSX 526 KB)

## Data Availability

Data will be made available on request.

## References

[1] Organisation for Economic Co-operation and Development (OECD). Reconciling terminology of the universe of per- and polyfluoroalkyl substances: recommendations and practical guidance. Paris: OECD Publishing. 2021. 10.1787/e458e796-en.

[2] Wang Z, Buser AM, Cousins IT, Demattio S, Drost W, Johansson O, Ohno K, Patlewicz G, Richard AM, Walker GW, White GS, Leinala E. A new OECD definition for per- and polyfluoroalkyl substances. Environ Sci Technol. 2021;55(23):15575–8. 10.1021/acs.est.1c06896. 34751569 10.1021/acs.est.1c06896

[3] Schymanski EL, Zhang J, Thiessen PA, Chirsir P, Kondic T, Bolton EE. Per- and polyfluoroalkyl substances (PFAS) in PubChem: 7 million and growing. Environ Sci Technol. 2023;57(44):16918–28. 10.1021/acs.est.3c04855. 37871188 10.1021/acs.est.3c04855 PMC10634333

[4] Buck RC, Franklin J, Berger U, Conder JM, Cousins IT, de Voogt P, Jensen AA, Kannan K, Mabury SA, van Leeuwen SP. Perfluoroalkyl and polyfluoroalkyl substances in the environment: terminology, classification, and origins. Integr Environ Assess Manag. 2011;7(4):513–41. 10.1002/ieam.258. 21793199 10.1002/ieam.258 PMC3214619

[5] European Chemicals Agency (ECHA) (2023) Annex XV restriction report: proposal for a restriction. https://echa.europa.eu/documents/10162/f605d4b5-7c17-7414-8823-b49b9fd43aea. Accessed 2023–12–21

[6] UN environment programm (UNEP) The 16 new POPS - an introduction to the chemicals added to the Stockholm Convention as persistant organic pollutants by the Conference of the Parties. https://www.pops.int/TheConvention/ThePOPs/TheNewPOPs/tabid/2511/Default.aspx. Accessed 2023–12–21.

[7] Fenton SE, Ducatman A, Boobis A, DeWitt JC, Lau C, Ng C, Smith JS, Roberts SM. Per- and polyfluoroalkyl substance toxicity and human health review: current state of knowledge and strategies for informing future research. Environ Toxicol Chem. 2021;40(3):606–30. 10.1002/etc.4890. 33017053 10.1002/etc.4890 PMC7906952

[8] Haukas M, Berger U, Hop H, Gulliksen B, Gabrielsen GW. Bioaccumulation of per- and polyfluorinated alkyl substances (PFAS) in selected species from the Barents Sea food web. Environ Pollut. 2007;148(1):360–71. 10.1016/j.envpol.2006.09.021. 17258363 10.1016/j.envpol.2006.09.021

[9] Dai Z, Xia X, Guo J, Jiang X. Bioaccumulation and uptake routes of perfluoroalkyl acids in Daphnia magna. Chemosphere. 2013;90(5):1589–96. 10.1016/j.chemosphere.2012.08.026. 22967930 10.1016/j.chemosphere.2012.08.026

[10] Sun JM, Kelly BC, Gobas F, Sunderland EM. A food web bioaccumulation model for the accumulation of per- and polyfluoroalkyl substances (PFAS) in fish: how important is renal elimination? Environ Sci Process Impacts. 2022;24(8):1152–64. 10.1039/d2em00047d. 35678632 10.1039/d2em00047d PMC9384792

[11] Brunn H, Arnold G, Körner W, Rippen G, Steinhäuser KG, Valentin I. PFAS: forever chemicals—persistent, bioaccumulative and mobile. Reviewing the status and the need for their phase out and remediation of contaminated sites. Environ Sci Eur. 2023;35(1):20. 10.1186/s12302-023-00721-8.

[12] Cousins IT, DeWitt JC, Gluge J, Goldenman G, Herzke D, Lohmann R, Ng CA, Scheringer M, Wang Z. The high persistence of PFAS is sufficient for their management as a chemical class. Environ Sci Process Impacts. 2020;22(12):2307–12. 10.1039/d0em00355g. 33230514 10.1039/d0em00355g PMC7784706

[13] Kissa E. Fluorinated surfactants and repellents. New York: Marcel Dekker. 2001.

[14] Glüge J, Scheringer M, Cousins IT, Dewitt JC, Goldenman G, Herzke D, Lohmann R, Ng CA, Trier X, Wang Z. An overview of the uses of per- and polyfluoroalkyl substances (PFAS). Environ Sci Process Impacts. 2020;22(12):2345–73. 10.1039/d0em00291g. 33125022 10.1039/d0em00291g PMC7784712

[15] Dauchy X, Boiteux V, Bach C, Rosin C, Munoz JF. Per- and polyfluoroalkyl substances in firefighting foam concentrates and water samples collected near sites impacted by the use of these foams. Chemosphere. 2017;183:53–61. 10.1016/j.chemosphere.2017.05.056. 28531559 10.1016/j.chemosphere.2017.05.056

[16] Houtz EF, Higgins CP, Field JA, Sedlak DL. Persistence of perfluoroalkyl acid precursors in AFFF-impacted groundwater and soil. Environ Sci Technol. 2013;47(15):8187–95. 10.1021/es4018877. 23886337 10.1021/es4018877

[17] Liu M, Glover CM, Munoz G, Duy SV, Sauve S, Liu J. Hunting the missing fluorine in aqueous film-forming foams containing per- and polyfluoroalkyl substances. J Hazard Mater. 2024;464:133006. 10.1016/j.jhazmat.2023.133006. 37988941 10.1016/j.jhazmat.2023.133006

[18] Weiner B, Yeung LW, Marchington EB, D’Agostino LA, Mabury SA. Organic fluorine content in aqueous film forming foams (AFFFs) and biodegradation of the foam component 6: 2 fluorotelomermercaptoalkylamido sulfonate (6: 2 FTSAS). Environ Chem. 2013;10(6):486–93.

[19] Prevedouros K, Cousins IT, Buck RC, Korzeniowski SH. Sources, fate and transport of perfluorocarboxylates. Environ Sci Technol. 2006;40(1):32–44. 10.1021/es0512475. 16433330 10.1021/es0512475

[20] Choi YJ, Helbling DE, Liu J, Olivares CI, Higgins CP. Microbial biotransformation of aqueous film-forming foam derived polyfluoroalkyl substances. Sci Total Environ. 2022;824:153711. 10.1016/j.scitotenv.2022.153711. 35149076 10.1016/j.scitotenv.2022.153711

[21] Place BJ, Field JA. Identification of novel fluorochemicals in aqueous film-forming foams used by the US military. Environ Sci Technol. 2012;46(13):7120–7. 10.1021/es301465n. 22681548 10.1021/es301465n PMC3390017

[22] Backe WJ, Day TC, Field JA. Zwitterionic, cationic, and anionic fluorinated chemicals in aqueous film forming foam formulations and groundwater from U.S. military bases by nonaqueous large-volume injection HPLC-MS/MS. Environ Sci Technol. 2013;47(10):5226–34. 10.1021/es3034999. 23590254 10.1021/es3034999

[23] Barzen-Hanson KA, Roberts SC, Choyke S, Oetjen K, McAlees A, Riddell N, McCrindle R, Ferguson PL, Higgins CP, Field JA. Discovery of 40 classes of per- and polyfluoroalkyl substances in historical aqueous film-forming foams (AFFFs) and AFFF-impacted groundwater. Environ Sci Technol. 2017;51(4):2047–57. 10.1021/acs.est.6b05843. 28098989 10.1021/acs.est.6b05843

[24] Gonda N, Choyke S, Schaefer C, Higgins CP, Voelker B. Hydroxyl radical transformations of perfluoroalkyl acid (PFAA) precursors in aqueous film forming foams (AFFFs). Environ Sci Technol. 2023;57(21):8053–64. 10.1021/acs.est.2c08689. 37200532 10.1021/acs.est.2c08689

[25] Moe MK, Huber S, Svenson J, Hagenaars A, Pabon M, Trumper M, Berger U, Knapen D, Herzke D. The structure of the fire fighting foam surfactant Forafac(R)1157 and its biological and photolytic transformation products. Chemosphere. 2012;89(7):869–75. 10.1016/j.chemosphere.2012.05.012. 22658941 10.1016/j.chemosphere.2012.05.012

[26] Cook EK, Olivares CI, Antell EH, Yi S, Nickerson A, Choi YJ, Higgins CP, Sedlak DL, Alvarez-Cohen L. Biological and chemical transformation of the six-carbon polyfluoroalkyl substance N-dimethyl ammonio propyl perfluorohexane sulfonamide (AmPr-FHxSA). Environ Sci Technol. 2022;56(22):15478–88. 10.1021/acs.est.2c00261. 36257682 10.1021/acs.est.2c00261

[27] Harding-Marjanovic KC, Houtz EF, Yi S, Field JA, Sedlak DL, Alvarez-Cohen L. Aerobic biotransformation of fluorotelomer thioether amido sulfonate (Lodyne) in AFFF-amended microcosms. Environ Sci Technol. 2015;49(13):7666–74. 10.1021/acs.est.5b01219. 26042823 10.1021/acs.est.5b01219

[28] Liu M, Munoz G, Vo Duy S, Sauve S, Liu J. Stability of nitrogen-containing polyfluoroalkyl substances in aerobic soils. Environ Sci Technol. 2021;55(8):4698–708. 10.1021/acs.est.0c05811. 33739092 10.1021/acs.est.0c05811

[29] Röhler K, Susset B, Grathwohl P. Production of perfluoroalkyl acids (PFAAs) from precursors in contaminated agricultural soils: batch and leaching experiments. Sci Total Environ. 2023;902:166555. 10.1016/j.scitotenv.2023.166555. 37633401 10.1016/j.scitotenv.2023.166555

[30] Higgins CP, Luthy RG. Sorption of perfluorinated surfactants on sediments. Environ Sci Technol. 2006;40(23):7521–7256. 10.1021/es061000n. 17180974 10.1021/es061000n

[31] Xiao F, Jin B, Golovko SA, Golovko MY, Xing B. Sorption and desorption mechanisms of cationic and zwitterionic per- and polyfluoroalkyl substances in natural soils: thermodynamics and hysteresis. Environ Sci Technol. 2019;53(20):11818–27. 10.1021/acs.est.9b05379. 31553179 10.1021/acs.est.9b05379

[32] Maizel AC, Shea S, Nickerson A, Schaefer C, Higgins CP. Release of per- and polyfluoroalkyl substances from aqueous film-forming foam impacted soils. Environ Sci Technol. 2021;55(21):14617–27. 10.1021/acs.est.1c02871. 34665614 10.1021/acs.est.1c02871

[33] Nickerson A, Maizel AC, Kulkarni PR, Adamson DT, Kornuc JJ, Higgins CP. Enhanced extraction of AFFF-associated PFASs from source zone soils. Environ Sci Technol. 2020;54(8):4952–62. 10.1021/acs.est.0c00792. 32200626 10.1021/acs.est.0c00792

[34] Munoz G, Ray P, Mejia-Avendano S, Vo Duy S, Tien Do D, Liu J, Sauve S. Optimization of extraction methods for comprehensive profiling of perfluoroalkyl and polyfluoroalkyl substances in firefighting foam impacted soils. Anal Chim Acta. 2018;1034:74–84. 10.1016/j.aca.2018.06.046. 30193642 10.1016/j.aca.2018.06.046

[35] Cao D, Schwichtenberg T, Duan C, Xue L, Muensterman D, Field J. Practical semiquantification strategy for estimating suspect per- and polyfluoroalkyl substance (PFAS) concentrations. J Am Soc Mass Spectrom. 2023. 10.1021/jasms.3c00019. 37018384 10.1021/jasms.3c00019

[36] D’Agostino LA, Mabury SA. Identification of novel fluorinated surfactants in aqueous film forming foams and commercial surfactant concentrates. Environ Sci Technol. 2014;48(1):121–9. 10.1021/es403729e. 24256061 10.1021/es403729e

[37] Luo Y-S, Aly NA, McCord J, Strynar MJ, Chiu WA, Dodds JN, Baker ES, Rusyn I. Rapid characterization of emerging per- and polyfluoroalkyl substances in aqueous film-forming foams using ion mobility spectrometry–mass spectrometry. Environ Sci Technol. 2020;54(23):15024–34. 10.1021/acs.est.0c04798. 33176098 10.1021/acs.est.0c04798 PMC7719402

[38] Liu M, Munoz G, Vo Duy S, Sauve S, Liu J. Per- and polyfluoroalkyl substances in contaminated soil and groundwater at airports: a Canadian case study. Environ Sci Technol. 2022;56(2):885–95. 10.1021/acs.est.1c04798. 34967613 10.1021/acs.est.1c04798

[39] Mejia-Avendaño S, Munoz G, Vo Duy S, Desrosiers M, BenoîTSauvéLiu PSJ. Novel fluoroalkylated surfactants in soils following firefighting foam deployment during the Lac-Mégantic Railway Accident. Environ Sci Technol. 2017;51(15):8313–23. 10.1021/acs.est.7b02028. 28669179 10.1021/acs.est.7b02028

[40] Bugsel B, Zweigle J, Zwiener C. Nontarget screening strategies for PFAS prioritization and identification by high resolution mass spectrometry: A review. Trends Environ Anal Chem. 2023;40. 10.1016/j.teac.2023.e00216.

[41] Bugsel B, Zwiener C. LC-MS screening of poly- and perfluoroalkyl substances in contaminated soil by Kendrick mass analysis. Anal Bioanal Chem. 2020;412(20):4797–805. 10.1007/s00216-019-02358-0. 31919607 10.1007/s00216-019-02358-0 PMC7334281

[42] Kaufmann A, Butcher P, Maden K, Walker S, Widmer M. Simplifying nontargeted analysis of PFAS in complex food matrixes. J AOAC Int. 2022;105(5):1280–7. 10.1093/jaoacint/qsac071. 35689643 10.1093/jaoacint/qsac071

[43] Zweigle J, Bugsel B, Zwiener C. Efficient PFAS prioritization in non-target HRMS data: systematic evaluation of the novel MD/C-m/C approach. Anal Bioanal Chem. 2023;415:1791–801. 10.1007/s00216-023-04601-1. 36826506 10.1007/s00216-023-04601-1 PMC10049945

[44] Zweigle J, Bugsel B, Zwiener C. FindPFΔS: non-target screening for PFAS─comprehensive data mining for MS2 fragment mass differences. Anal Chem. 2022;94(30):10788–96. 10.1021/acs.analchem.2c01521. 35866933 10.1021/acs.analchem.2c01521 PMC9354793

[45] Zweigle J, Bugsel B, Fabregat-Palau J, Zwiener C. PFΔScreen - an open-source tool for automated PFAS feature prioritization in non-target HRMS data. Anal Bioanal Chem. 2024;416(2):349–62. 10.1007/s00216-023-05070-2. 38030884 10.1007/s00216-023-05070-2 PMC10761406

[46] Place B. Suspect list of possible per- and polyfluoroalkyl substances (PFAS). National Inst Stand Technol. 2021. https://data.nist.gov/od/id/mds2-2387. Accessed 2023–12–15.

[47] Menin L, Ortiz D, Gasilova N, Sepulved F, Patiny L MSTools. https://mstools.epfl.ch/monoisotopic/. Accessed 2024–03–11.

[48] Patiny L, Borel A. ChemCalc: a building block for tomorrow’s chemical infrastructure. J Chem Inf Model. 2013;53(5):1223–8. 10.1021/ci300563h. 23480664 10.1021/ci300563h

[49] Pluskal T, Uehara T, Yanagida M. Highly accurate chemical formula prediction tool utilizing high-resolution mass spectra, MS/MS fragmentation, heuristic rules, and isotope pattern matching. Anal Chem. 2012;84(10):4396–403. 10.1021/ac3000418. 22497521 10.1021/ac3000418

[50] Ruttkies C, Schymanski EL, Wolf S, Hollender J, Neumann S. MetFrag relaunched: incorporating strategies beyond in silico fragmentation. J Cheminform. 2016;8(1):3. 10.1186/s13321-016-0115-9. 26834843 10.1186/s13321-016-0115-9 PMC4732001

[51] Charbonnet JA, McDonough CA, Xiao F, Schwichtenberg T, Cao D, Kaserzon S, Thomas KV, Dewapriya P, Place BJ, Schymanski EL, Field JA, Helbling DE, Higgins CP. Communicating confidence of per- and polyfluoroalkyl substance identification via high-resolution mass spectrometry. Environ Sci Technol Lett. 2022;9(6):473–81. 10.1021/acs.estlett.2c00206. 35719859 10.1021/acs.estlett.2c00206 PMC9202347

[52] Schymanski EL, Jeon J, Gulde R, Fenner K, Ruff M, Singer HP, Hollender J. Identifying small molecules via high resolution mass spectrometry: communicating confidence. Environ Sci Technol. 2014;48(4):2097–8. 10.1021/es5002105. 24476540 10.1021/es5002105

[53] Yan PF, Dong S, Pennell KD, Capiro NL. A review of the occurrence and microbial transformation of per- and polyfluoroalkyl substances (PFAS) in aqueous film-forming foam (AFFF)-impacted environments. Sci Total Environ. 2024;927:171883. 10.1016/j.scitotenv.2024.171883. 38531439 10.1016/j.scitotenv.2024.171883

[54] Li Z, Lu Y, Chen T, He A, Huang Y, Li L, Pan W, Li J, Zhu N, Wang Y, Jiang G. Generation mechanism of perfluorohexanesulfonic acid from polyfluoroalkyl sulfonamide derivatives during chloramination in drinking water. Environ Sci Technol. 2023;57(47):18462–72. 10.1021/acs.est.2c07881. 36633968 10.1021/acs.est.2c07881

[55] D’Agostino LA, Mabury SA. Aerobic biodegradation of 2 fluorotelomer sulfonamide-based aqueous film-forming foam components produces perfluoroalkyl carboxylates. Environ Toxicol Chem. 2017;36(8):2012–21. 10.1002/etc.3750. 28145584 10.1002/etc.3750

[56] Yukioka S, Tanaka S, Suzuki Y, Fujii S, Echigo S. A new method to search for per- and polyfluoroalkyl substances (PFASs) by linking fragmentation flags with their molecular ions by drift time using ion mobility spectrometry. Chemosphere. 2020;239:124644. 10.1016/j.chemosphere.2019.124644. 31514004 10.1016/j.chemosphere.2019.124644

[57] Fang B, Zhang Y, Chen H, Qiao B, Yu H, Zhao M, Gao M, Li X, Yao Y, Zhu L, Sun H. Stability and biotransformation of 6:2 fluorotelomer sulfonic acid, sulfonamide amine oxide, and sulfonamide alkylbetaine in aerobic sludge. Environ Sci Technol. 2024;58(5):2446–57. 10.1021/acs.est.3c05506. 38178542 10.1021/acs.est.3c05506

[58] Ruyle BJ, Thackray CP, McCord JP, Strynar MJ, Mauge-Lewis KA, Fenton SE, Sunderland EM. Reconstructing the composition of per- and polyfluoroalkyl substances in contemporary aqueous film-forming foams. Environ Sci Technol Lett. 2021;8(1):59–65. 10.1021/acs.estlett.0c00798. 33628855 10.1021/acs.estlett.0c00798 PMC7898139

[59] Olivares CI, Yi S, Cook EK, Choi YJ, Montagnolli R, Byrne A, Higgins CP, Sedlak DL, Alvarez-Cohen L. Aerobic BTEX biodegradation increases yield of perfluoroalkyl carboxylic acids from biotransformation of a polyfluoroalkyl surfactant, 6:2 FtTAoS. Environ Sci Process Impacts. 2022;24(3):439–46. 10.1039/d1em00494h. 35113105 10.1039/d1em00494h

