# Supplementary material for: Non-target screening reveals 124 PFAS at an AFFF-impacted field site in Germany specified by novel systematic terminology
Source: Anal Bioanal Chem. 2024 Oct 28;417(27):6049–64. doi: 10.1007/s00216-024-05611-3 (PMC12583289; doi:10.1007/s00216-024-05611-3)
Supplement: Supplementary file 1 — Supplementary file1 (PDF 6.29 MB) [file 216_2024_5611_MOESM1_ESM.pdf]

**Analytical and Bioanalytical Chemistry**

**Electronic Supplementary Material 1**

# Non-target Screening reveals 124 PFAS at an AFFF impacted field site in Germany specified by a novel systematic terminology

Melanie Schüßler[1], Catharina Capitain[1], Boris Bugsel[1], Jonathan Zweigle[1], Christian Zwiener[1]

1 Environmental Analytical Chemistry, Department of Geosciences, University of Tübingen, Schnarrenbergstraße 94-96, 72076 Tübingen, Germany

# Table of content


ESM 1.A Chemicals ........ 3

ESM 1.B Sampling site ........ 5

ESM 1.C Feature prioritization ........ 6

ESM 1.D Confidence level ........ 7

ESM 1.E Extraction method ........ 10

ESM 1.G Fragmentation evidence, chromatograms, and $MS^2$ spectra ........ 11

# ESM 1.A Chemicals

**Table S1:** Overview about all PFAS reference standards that were included in the PFAS standard mixture and where they were purchased. PFAS originated either from Wellington Laboratories, Guelph, Ontario, Canada (1), Toronto Research Chemicals, North York, Ontario, Canada (2), Dr. Ehrenstorfer, Augsburg, Bavaria, Germany (3) or were custom-synthesized in-house (4).

| Acronym | Used standard / purchased chemical | Origin |
|---|---|---|
| **PFCAs** | | |
| PFBA | Perfluorobutanoic acid / perfluoro-n-butanoic acid | (1) |
| PFPeA | Perfluoropentanoic acid / perfluoro-n-pentanoic acid | (1) |
| PFHxA | Perfluorohexanoic acid / perfluoro-n-hexanoic acid | (1) |
| PFHpA | Perfluoroheptanoic acid / perfluoro-n-heptanoic acid | (1) |
| PFOA | Perfluorooctanoic acid / perfluoro-n-octanoic acid | (1) |
| PFNA | Perfluorononanic acid / perfluoro-n-nonanic acid | (1) |
| PFDA | Perfluorodecanoic acid / perfluoro-n-decanoic acid | (1) |
| PFUnDA | Perfluoroundecanoic acid / perfluoro-n-undecanoic acid | (1) |
| PFDoDA | Perfluorododecanoic acid / perfluoro-n-dodecanoic acid | (1) |
| PFTriDA | Perfluorotridecanoic acid / perfluoro-n-tridecanoic acid | (1) |
| PFTeDA | Perfluorotetradecanoic acid / perfluoro-n-tetradecanoic acid | (1) |
| PFHxDA | Perfluorohexadecanoic acid / perfluoro-n-hexadecanoic acid | (1) |
| PFODA | Perfluorooctadecanoic acid / perfluoro-n-octadecanoic acid | (1) |
| **PFSAs** | | |
| PFBS | Perfluorobutanesulfonic acid / potassium perfluoro-1-butanesulfonate | (1) |
| PFPeS | Perfluoropentanesulfonic acid / sodium perfluoro-1-pentanesulfonate | (1) |
| PFHxS | Perfluorohexanesulfonic acid / sodium perfluoro-1-hexanesulfonate | (1) |
| PFHpS | Perfluoroheptanesulfonic acid / sodium perfluoro-1-heptanesulfonate | (1) |
| PFOS | Perfluorooctanesulfonic acid / sodium perfluoro-1-octanesulfonate | (1) |
| PFNS | Perfluorononanesulfonic acid / sodium perfluoro-1-nonanesulfonate | (1) |
| PFDS | Perfluorodecanesulfonic acid / sodium perfluoro-1-decanesulfonate | (1) |
| PFDoDS | Perfluorododecanesulfonic acid / sodium perfluoro-1-dodecanesulfonate | (1) |
| **PFPAs** | | |
| PFOPA | Perfluorooctylphosphonic acid | (1) |
| PFDPA | Perfluorodecylphosphonic acid | (1) |
| **PAPs** | | |
| 6:2/6:2 diPAP | 6:2/6:2 phosphoric acid diester / Bis[2-(perfluorohexyl)ethyl] phosphate | (2) |
| 8:2/8:2 diPAP | 6:2/6:2 polyfluoroalkyl phosphoric acid diester / Sodium bis (1H, 1H, 2H, 2H-perfluorodecyl) phosphate | (1) |
| 6:2 PAP | 6:2 polyfluoroalkyl phosphoric ester / Mono[2-(perfluorohexyl)ethyl] phosphate | (2) |

| | | |
|---|---|---|
| 8:2 PAP | 6:2 polyfluoroalkyl phosphoric ester / Sodium 1H, 1H, 2H, 2H-perfluorodecyl phosphate | (1) |
| **PFPiAs** | | |
| C6/C6 PFPiA | C6/C6 Perfluoroalkyl phosphinic acid | (2) |
| **PASF-based PFAS** | | |
| PFHxSAm | Perfluorohexane sulfonamide | (3) |
| PFOSAm | Perfluorooctane sulfonamide | (1) |
| PFOSAm-*N*-Et-*N*-EtA | Perfluorooctane sulfonamide *N*-ethyl *N*-ethanoic acid | (1) |
| SamPAP | Perfluorooctane sulfonamide ethanol-based phosphate diester /Sodium-2-(N-ethylperfluorooctane-1-sulfonamido) ethyl phosphate | (1) |
| diSAmPAP | Perfluorooctane sulfonamide ethanol-based phosphate diester/ Sodium bis[2-(N-ethylperfluorooctane-1-sulfonamido) ethyl] phosphate | (1) |
| **FTCAs** | | |
| 6:2 FTCA | 6:2 fluorotelomer carboxylic acid / 2-Perfluorohexyl ethanoic acid (6:2) | (1) |
| 8:2 FTCA | 8:2 fluorotelomer carboxylic acid / 2-Perfluorooctyl ethanoic acid (6:2) | (1) |
| 5:3 FTCA | 5:3 fluorotelomer carboxylic acid/ 3-Perfluoropentyl propanoic acid | (1) |
| 7:3 FTCA | 7:3 fluorotelomer carboxylic acid/ 3-Perfluoroheptyl propanoic acid | (1) |
| **FTUCAs** | | |
| 6:2 FTUCA | 6:2 fluorotelomer unsaturated carboxylic acid/ 2H-Perfluoro-2-octenoic acid (6:2) | (1) |
| 8:2 FTUCA | 8:2 fluorotelomer unsaturated carboxylic acid/ 2H-Perfluoro-2-decenoic acid (8:2) | (1) |
| **FTSAs** | | |
| 6:2 FTSA | 6:2 fluorotelomer sulfonic acid / Sodium 1H, 1H,2H,2H-perfluorooctanesulfonate | (1) |
| 8:2 FTSA | 8:2 fluorotelomer sulfonic acid / Sodium 1H, 1H,2H,2H-perfluorodecanesulfonate | (1) |
| **FTMAPs** | | |
| 6:2 FTMAP | 6:2 Fluorotelomer mercapto alkyl phosphate | (4) |
| **PFECAs** | | |
| HFPO-Da | 2,3,3,3-Tetrafluoro-2-(1,1,2,2,3,3,3-heptafluoropropoxy) propanoic acid | (1) |
| ADONA | Sodium dodecafluoro-3H-4,8-dioxanonanoate | (1) |
| **PFESAs** | | |
| 9Cl-PF3ONS | Potassium 9-chlorhexadecafluoro-3-oxanonane-1-sulfonate | (1) |
| 11Cl-PF3OUdS | Potassium 11-chloroeicosafluoro-3-oxaundecane-1-sulfonate | (1) |
| **AFFF-substances** | | |
| 6:2 FTSAm-Pr-DiMeNO | 6:2 fluorotelomer sulfonamide propyl methylamineoxide / Capstone product A | (3) |
| 6:2 FTSAm-Pr-B | 6:2 fluorotelomer sulfonamide propyl betaine / Capstone product B | (3) |
| PFHxSAm-Pr-DiMeAm | N-[3(dimethylamino)propyl] perfluoro-1-hexanesulfonamide | (3) |

| | | |
|---|---|---|
| 5:3 FTB | 5:3 Fluorotelomer betaine / 2-[4,4,5,5,6,6,7,7,8,8,8-Undecafluorooctyl) dimethylammonio] acetate | (1) |
| 5:1:2 FTB | 5:1:2 Fluorotelomer etaine / 2-[(3,4,4,5,5,6,6,7,7,8,8,8-Dodecafluorooctyl) dimethylammonio] acetate | (1) |

# ESM 1.B Sampling site

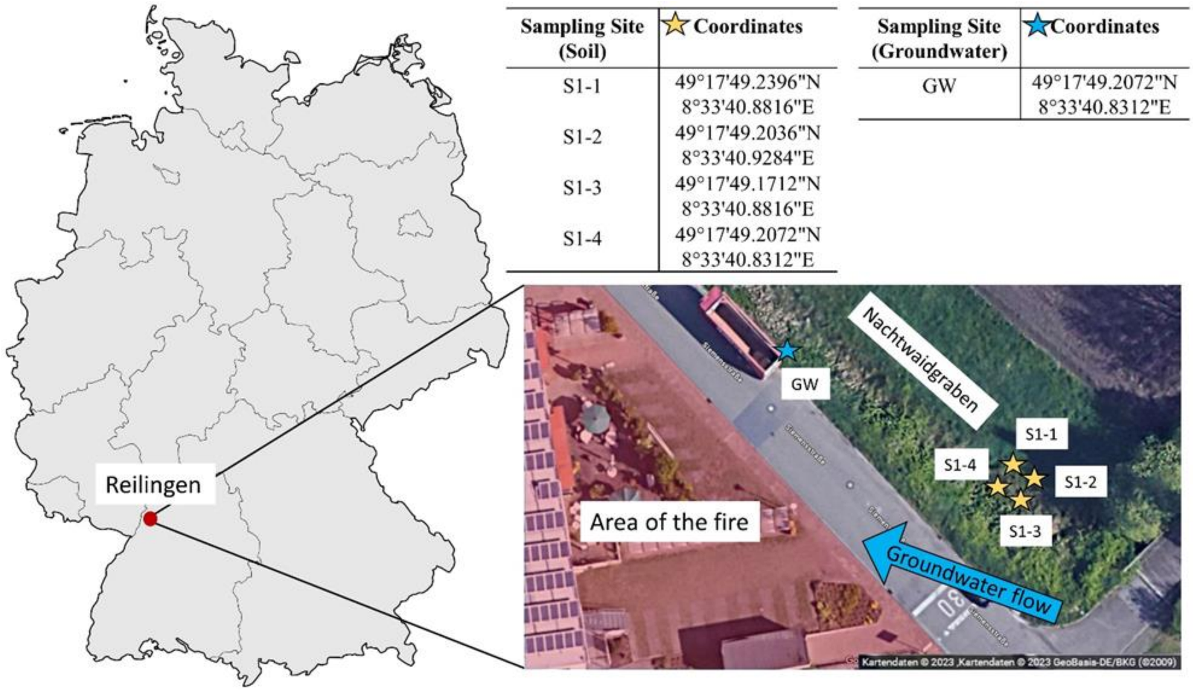


**Fig. S1:** Overview of the sampling site *Nachtwaidgraben* in Reilingen, Baden Württemberg and coordinates of the respective sampling points. Yellow stars represent soil sampling and blue stars represent groundwater sampling. The screenshot of the field site was taken from Google Maps (15.12.2023). Groundwater flow was determined by Arcadis.

# ESM 1.C Feature prioritization

The prioritization workflow (RT > 4 min, m/C > 23) was validated by application to a standard mix sample consisting of 49 negative and 5 positive ionizable PFAS. For negative ionizable PFAS the false positive rate was 8.3% and the false negative rate 0.034% (related to peak areas). For positive ionizable PFAS the false positive rate was 31.8% and the false negative rate 2.4%. The high false positive rate likely results from the low number of available positively ionizing PFAS standards. Manual inspection of the false positively prioritized features revealed that most of them did not show a gaussian peak shape and thus would not have been further considered for identification.

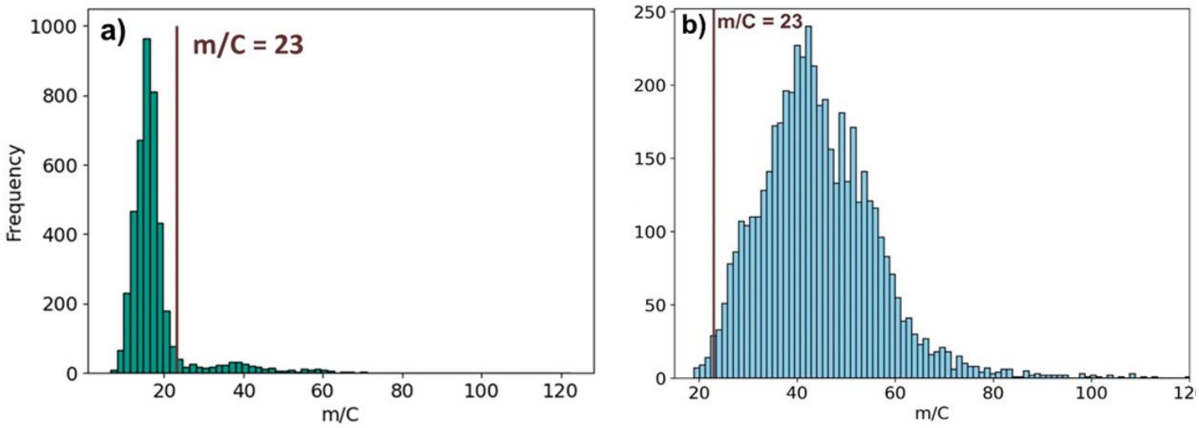


**Fig. S2:** a) Histogram of m/C values of all features of soil *S1* in the output table of PFΔ*Screen* before prioritization. The red line at m/C = 23 represents the cutoff for the prioritization. b) Histogram of m/C values of all PFAS included in the NIST suspect list [1]. The red line indicates an m/C value of 23, revealing that only a small fraction of PFAS from the list would be missed with a cutoff at m/C = 23.

## ESM 1.D Confidence level

The classification into confidence levels considered matching reference standards, accurate mass (< 10 ppm) and isotopic pattern match (Eq. 1, > 95%) for proposed formulas, m/C (> 25), diagnostic fragments, homologous series, and experimental data (see Table S2). The type of $MS^2$ fragment is not specifically named, but diagnostic fragments are expected to provide evidence for the proposed structure. PFAS that are ionizable in both ESI- and ESI+ mode give extra evidence for the proposed structure. Therefore, matching $MS^2$ fragments from positive and negative measurements are summed for the determination of the confidence level. Experimental data from the literature or from own experiments (e.g. the presence of similar structures) may be additionally available to support the proposed structure.

Level 1 compounds are confirmed by analytical reference standards. For all substances classified at level 2, a molecular formula and a specific structure are assigned, supported by accurate mass, isotopic pattern matching, diagnostic fragments (minimum of 3 fragments), and/or homologues (minimum of 3 homologues, with at least one at level 2 or higher). For all substances classified at level 3, a molecular formula and one or more structures are suggested, evidenced by accurate mass, isotopic pattern matching, diagnostic fragments (minimum of 2 fragments), and/or homologues (minimum of 2 homologues, with at least one at level 3 or higher). Level 2 PFAS are more reliable than level 3 PFAS, as more evidence is available. In addition, only one structure is suggested for level 2 PFAS, whereas several structures are possible for level 3. Unlike constitutional isomers, positional isomers only differ by the position of the same moiety or substituent (unsaturated bond, keto-/methyl-/hydroxy-group, $SF_5$-/Cl-/H-substitution). Positional isomers with varying possible structures are therefore grouped together and one exemplary structure is shown (ESM 2). The only uncertainty in the structure is the exact position of the moiety or substituent and can therefore still be assigned a confidence level of 2. The same approach was applied to ECF-based substances where

different variations of a structure are possible due to branching in the perfluoroalkyl chain. Level 4 compounds are assigned a matching molecular formula with accurate mass and isotopic pattern match. Although no structures can be directly inferred from $MS^2$ spectra, structures can still be proposed for some compounds that have already been found in the literature and thus are evidently TPs or precursors of other identified (level 1 – 3) compounds that were identified in the samples. Features are selected as level 5 PFAS due to matching mass defects (-0.15 – 0.15 Da), PFAS typical m/C ratios (m/C > 30), homologous series, and/or typical $MS^2$ fragments.

**Table S2:** Applied confidence level modified according to Charbonnet et al. [2]. Only one structure is suggested for level 2 PFAS, whereas several structures are possible for level 3. Positional isomers or branched perfluoroalkyl chains are grouped together.

| Level | Definition | Reference Standard | Accurate Mass (< 10 ppm) | Isotopic Pattern Match (> 95%) | m/C (> 25) | Homologues (number) with consistent RT | $MS^2$ Fragments (number) | Experimental Data |
|---|---|---|---|---|---|---|---|---|
| **1** | Confirmed by reference standard | ✓ | ✓ | ✓ | ✓ | (✓) | (✓) | (✓) |
| **2** | Probable structure by diagnostic fragmentation evidence | **x** | ✓ | ✓ | ✓ | **x** | **≥ 3** | (✓) |
| | Probable structure by diagnostic homologue evidence | **x** | ✓ | ✓ | ✓ | **≥ 3** (at least one homologue at level ≥ 2) | **x** | (✓) |
| **3** | Circumstantial candidates with fragmentation evidence | **x** | ✓ | ✓ | ✓ | **x** | **≥ 2** | (✓) |
| | Circumstantial candidates with homologue evidence | **x** | ✓ | ✓ | ✓ | **≥ 2** (at least one homologue at level ≥ 3) | **x** | (✓) |
| **4** | Matching molecular formula | **x** | ✓ | ✓ | ✓ | **x** | **x** | (✓) |
| **5** | Nontarget PFAS exact mass of interest | At least one of condition must apply: PFAS typical mass defect, PFAS typical m/C ratio, homologous series, typical $MS^2$ fragments | | | | | | |

# ESM 1.E Extraction method

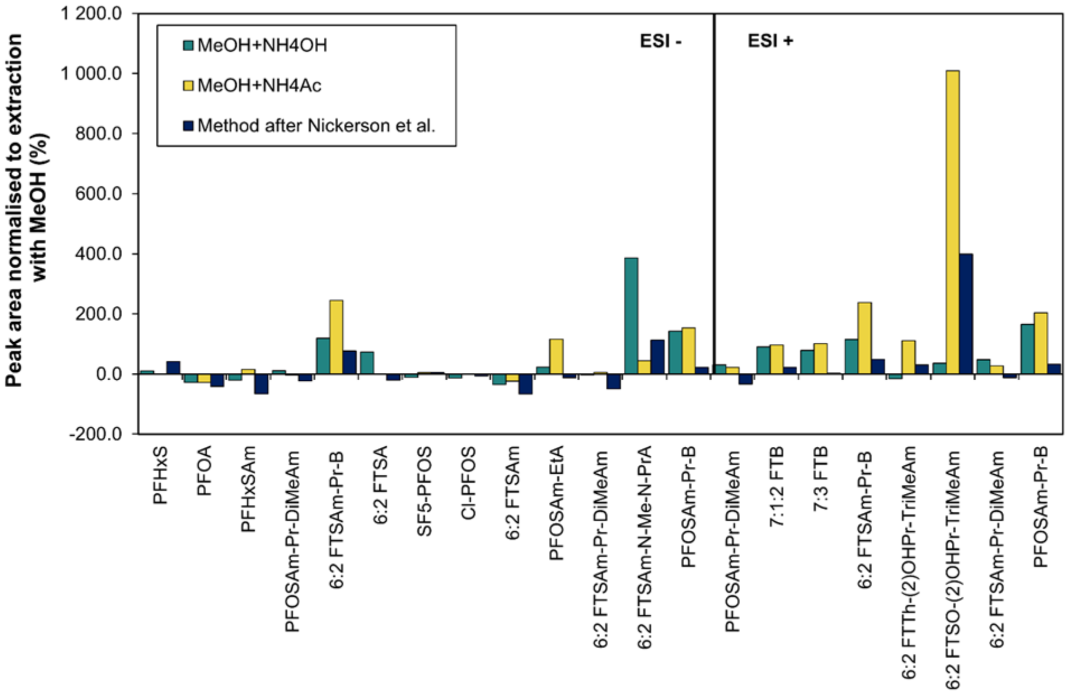


**Fig. S3:** Comparison of extraction efficiencies for 19 different PFAS with MeOH+$NH_4OH$ or MeOH+$NH_4Ac$ as extraction solvent and the extraction method after Nickerson et al. [3] relative to the extraction with pure MeOH in negative ionization mode (ESI-, left side) and positive ionization mode (ESI+, right side). Note: substances that appear in both ionization modes are zwitterionic substances.

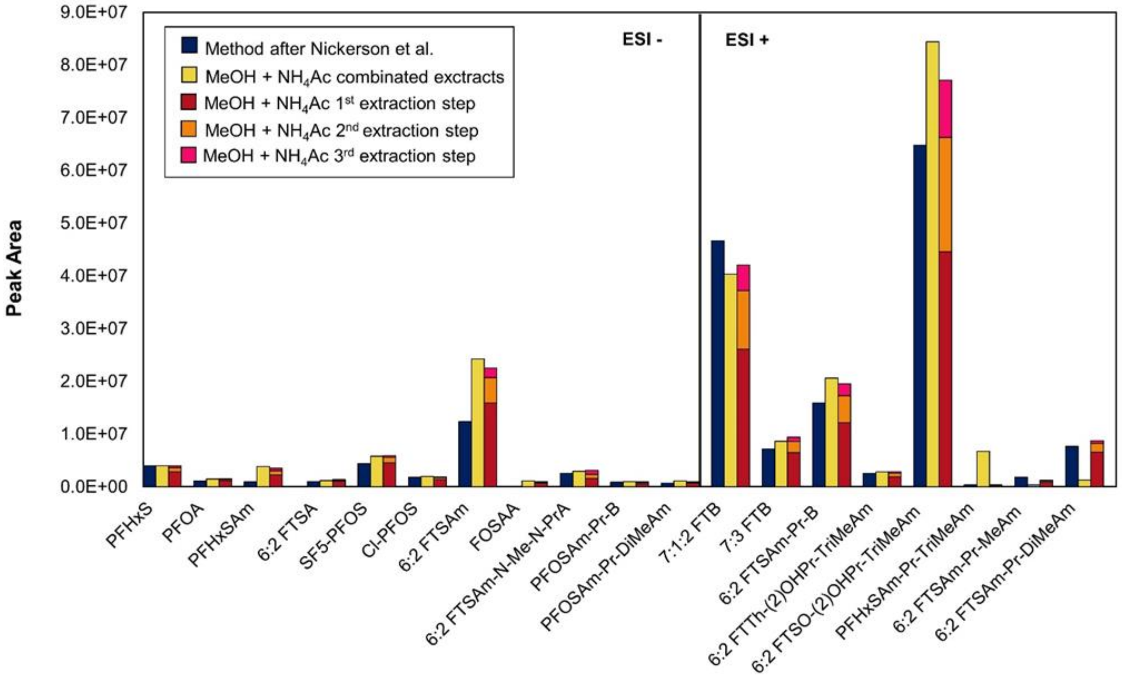


**Fig. S4:** Comparison of extraction efficiencies for 19 different PFAS with MeOH+$NH_4Ac$ as extraction solvent and a three-step extraction and the extraction method after Nickerson et al. [3] in negative ionization mode (ESI-, left side) and positive ionization mode (ESI+, right side).

# ESM 1.G Fragmentation evidence, chromatograms, and MS$^2$ spectra

**PFSA and U-PFSA derivatives (Table 3, row 2, 3, and 11): Cl-PFSAs, $SF_5$-PFSAs, H-PFSAs, U-PFSAs, and $SF_5$-U-PFSAs, PFASyAs (all ESI-)**

The diagnostic fragment $[SO_3]^-$ indicates a sulfonic acid in a molecule and the fragments $[SO_3F]^-$ or $[SO_2F]^-$ indicate a $SO_3$ or $SO_2$ group being located in direct vicinity to the fluorinated part of the molecule. All PFSA derivatives but PFASyA show the diagnostic fragment $[SO_3]^-$. The $[SO_2F]^-$ fragment was observed for PFOSyA (Fig. S23b) and the $[SO_3F]^-$ fragment was observed for all other PFSA subclasses but $SF_5$-U-PFSAs. Fragments of the fluorinated chain with mass fragment differences of $\Delta = 49.9968$ ($CF_2$) are also indicative for PFSA fragmentation. This was observed for $SF_5$-PFSAs (Fig. S20b, c), U-PFSAs (Fig. S24b, c), $SF_5$-U-PFSAs (Fig. S25b, c), Cl-PFSAs (Fig. S19c) and PFOSyA (Fig. S23b). In addition, attached moieties to the fluorinated chain such as $[SO_3Cl]^-$ (Cl-PFOS, Fig. S19c), $[SF_5]^-$/$[M-SF_5]^-$ ($SF_5$-PFSAs and $SF_5$-U-PFSAs, Fig. S20b, c and S24b, c) or $[SO_3H]^-$ (H-PFOS, Fig. S21b) can reveal valuable structural information and be matched to the proposed structure.

For Cl-PFOS, which shows a split peak two possible positional isomers could be identified due of differing MS$^2$ spectra (Fig. S19a-c). The spectrum obtained from the second peak indicates that the Cl-substitution is located directly next to the sulfonic acid group (fragment $[SO_3Cl]^-$ Fig. S19c, [4]), while the spectrum obtained from the first peak does not show this fragment (Fig. S19b), indicating the position of the Cl-substitution being at the end of the perfluoroalkyl chain. However, since it is unlikely that an RT shift of this magnitude results from the occurrence of only two positional isomers, it is possible that a mixture of linear and branched Cl-PFOS isomers is present, which is responsible for the split peak.

**PFASAm and PFASAm-Pr-based AFFF PFAS (Table 3, row 4 and 5): PFASAm-EtA (ESI-), PFASAm-PrSA (ESI-), PFASAm-Pr-B (ESI-/ESI+), PFASAm-Pr-TriMeAm (ESI+)**

PFASAm-EtA and PFASAm-PrSA both show the diagnostic fragment $[SO_2N]^-$ indicating the presence of the sulfonamide group (Fig. S28b, c and S29b) and fragments of the fluorinated chain with characteristic mass differences of $\Delta CF_2$. Also, both subclasses show fragments of PFASAm itself (e.g. $[C_6HF_{13}NO_2S]^-$ for n = 6, Fig. S26b and S29b), corresponding to $[M\text{-}CH_2COO]^-$ for PFASAm-EtA and $[M\text{-}C_3H_6SO_3]^-$ for PFASAm-PrSA. As both subclasses do not coelute with PFASAm itself, interference of PFASAm with the $MS^2$ spectra of the 2 subclasses can be excluded.

PFASAm-Pr-B is zwitterionic but ionizes better in positive mode (Fig. S30a, b). Thus, fragmentation spectra are only available in ESI+ for PFASAm-Pr-B. Fragments distinctive for the betaine moiety ($[C_4H_8NO_2]^+$, $[C_4H_{10}NO_2]^+$, $[C_5H_{12}NO_2]^+$) were observed (Fig. S30c, d) and three further insightful fragments such as $[M\text{-}CO_2]^-$ (599.0656 Da) could be assigned to the structure (Fig. S30d). For PFASAm-Pr-TriMeAm three diagnostic fragments confirming the presence of the sulphonamide and propyl trimethylamine moieties could be observed for the n = 8 homologue (Fig. S31b).

**FTBs (Table 2, row 2 and Table 3, row 6): n:2 FTBs, n:1:3 FTBs, n:4 FTBs, n:3 FTBs, n:1:2 FTBs (all ESI+)**

Fragmentation evidence for FTBs is discussed in the main document.

**FTSA derivatives (Table 2, row 3 and Table 3, row 10): K-n:2 FTSAs, U-n:2 FTSAs, OH-n:2 FTSAs (all ESI-)**

Fragmentation evidence for K-n:2 and U-n:2 FTSAs is discussed in the main document. OH-n:2 FTSA shows diagnostic fragments of the sulfonic acid group ($[SO_3]^-$ and $[CH_3SO_3]^-$) as

well as the sulfonic acid group plus the OH-substitution ($[C_2H_3O_4S]^-$, Fig. S46b, c). Fragments of the fluorinated chain were also observed. Further, a neutral loss of water ($[M\text{-}H_2O]^-$) could be identified in the $MS^2$ spectra of both homologues, matching the proposed structure (e.g. $[C_8H_2F_{13}O_3S]^-$ for OH-6:2 FTSA, Fig. S46b).

**FTSAm derivatives (Table 2, row 1 and Table 3, row 7): n:2/m:2 FTSAm dimer, n:2 FTSAm-PrA, n:2 FTSAm-U-Pr-DiMeAm, n:2 FTSAm-*N*-Me-*N*-PrA, n:2 FTSAm-PrA (ESI-), n:2 FTSAm-Pr-MeAm (ESI+), n:2 FTSAm-Pr-DiMeAm (ESI-/ESI+), and n:2 FTSAm-U-Pr-DiMeAm (ESI-)**

Fragmentation evidence for n:2/m:2 FTSAm dimer, n:2 FTSAm-PrA, n:2 FTSAm-U-Pr-DiMeAm, and n:2 FTSAm-*N*-Me-*N*-PrA is discussed in the main document.

Three diagnostic fragments reveal the presence of a sulfonamide propyl trimethylamine moiety ($[C_4H_{11}N\cdot]^+$, $[C_5H1_2N]^+$ and $[C_6H_{16}N_2]^+$) for the n = 8 homologue of n:2 FTSAm-Pr-MeAm (Fig. S39b).

n:2 FTSAm-Pr-DiMeAm is a zwitterionic substance but ionizes much better in ESI+ (Fig. S40a). Fragmentation evidence could be retrieved from both ionization modes for the n = 6 homologue (Fig. S40b, c). In ESI-, the fragmentation spectra is characterized by a series of 4 HF losses starting from the molecular precursor ion ($[M\text{-}2H]^-$, Fig. S40b) as well as the diagnostic fragment $[C_5H_{13}N_2O_2S]^-$ which is indicative for the sulfonamide propyl dimethyl amine moiety. In ESI+ n:2 FTSAm-Pr-DiMeAm shows various diagnostic fragments indicative for the propyl dimethylamine moiety (Fig. S35c, d) as well as two further fragments supporting the structure for the n = 6 homologue (Fig. S40c).

**FTSy derivatives (Table 2, row 4 and Table 3, row 8): n:2 FTSy-PrA, n:2 FTSy-(2')OHPr-TriMeAm, n:2 FTSy-Pr-Ad-(5',5')DiMeEtSA**

Fragmentation evidence of n:2 FTSy-(2')OHPr-TriMeAm is discussed in the main document. For the n = 8 homologue of n:2 FTSy-PrA a series of 4 HF losses could be observed, matching the proposed structure. In addition, the diagnostic fragment $[C_3H_5O_2]^-$ could be identified confirming the presence of the propanoic acid group (Fig. S41c).

For n:2 FTSy-Pr-Ad-(5',5')DiMeEtSA a total of four diagnostic fragments matching the proposed structure was identified for the n = 6 and n = 8 homologues, respectively ($[SO_3]^-$, $[C_4H_7O_3S]^-$, $[C_4H_{10}NO_3S]^{-\cdot}$, $[C_7H_{12}NO_4S]^-$, Fig. S42b, c).

**FTSO derivatives (Table 3, row 9): n:2 FTSO-(2')OHPr-TriMeAm (ESI+) and n:2 FTSO-Pr-Ad-(5',5')DiMeEtSA (ESI-)**

For the n = 6 and n = 8 homologues of n:2 FTSO-(2')OHPr-TriMeAm, a total of 10 $MS^2$ fragments could be matched to the proposed structure, respectively (Fig. S43b, c). The most prominent two diagnostic fragments being $[C_6H_{16}NO_2S]^+$ (166.0896 Da) and $[C_6H_{14}NO]^+$ (116.1070).

For n:2 FTSO-Pr-Ad-(5',5')DiMeEtSA a total of 5 fragments could be matched the proposed structure for the n = 6 homologue (Fig. S44b). The fragment with the highest intensity nearly reveals the complete non-fluorinated part of the molecule ($[C_7H_{14}NO_5S_2]^-$, 256.0319 Da).

**FTTh derivatives (Table 2, row 11): n:2 FTTh-(2')OHPr-TriMeAm (ESI+)**

The n = 6 and n = 8 homologues of n:2 FTTh-(2')OHPr-TriMeAm do not show any diagnostic fragments but 3 expressive fragments with higher molecular weights, respectively (Fig. S45b, c).

### ***Chromatograms and MS² spectra***

Chromatograms of all confirmed and potential PFAS with proposed names are displayed below (chromatograms of "unknown" potential PFAS not shown) as well as $MS^2$ spectra of all level 2 and 3 PFAS subclasses, if available with two homologues and in positive and negative ionization mode. The precursor ions are indicated by blue diamonds. The best chromatograms/$MS^2$ spectra from site *S1*, depths, and extracts (first extract/combined extract) were displayed.

# Newly identified Substances

**n:2/m:2 FTSAm dimers (n = 6, 8 / m = 6)**

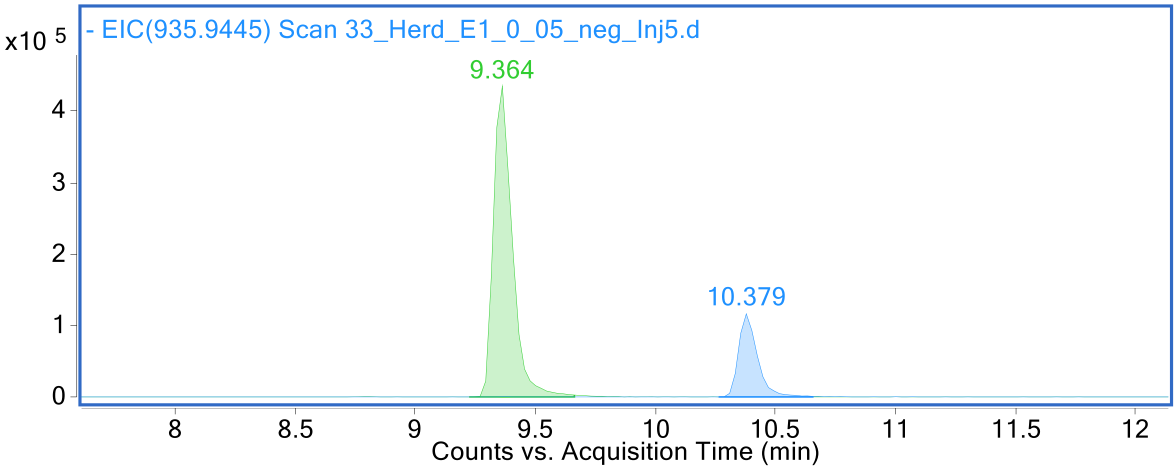


**Fig. S5a:** Chromatogram (ESI$^-$, soil *S1*, 0 – 0.5 m, first extract) of n:2/6:2 FTSAm dimers (n = 6 (green, m/z 835.9485, 9.364 min) and 8 (blue, m/z 935.9421, 10.379 min)).

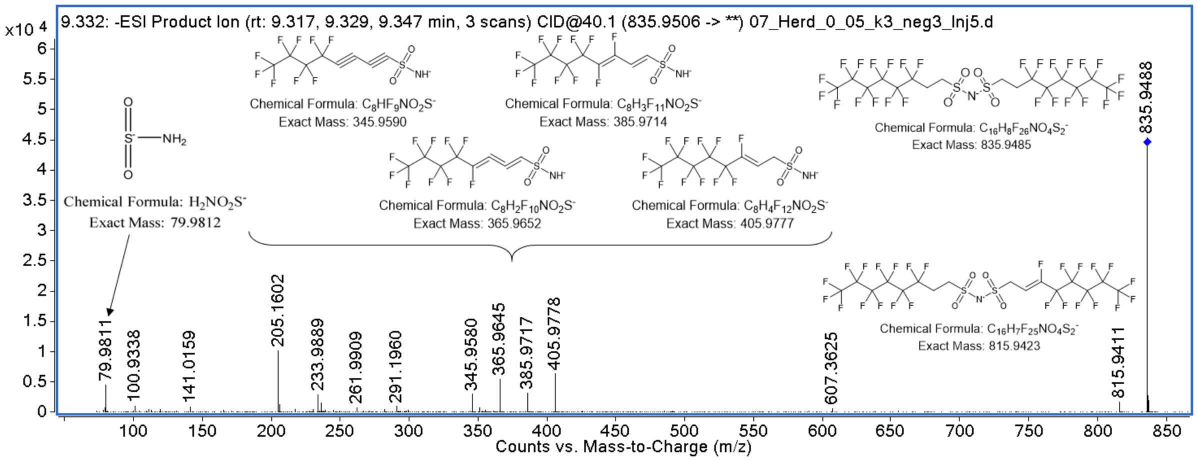


**Fig. S5b:** $MS^2$ spectrum (ESI$^-$, 40.1 eV, soil *S1*, 0 – 0.5 m, combined extract, iterative $MS^2$) of 6:2/6:2 FTSAm dimer (m/z 835.9485, 9.317, 9.329, and 9.347 min).

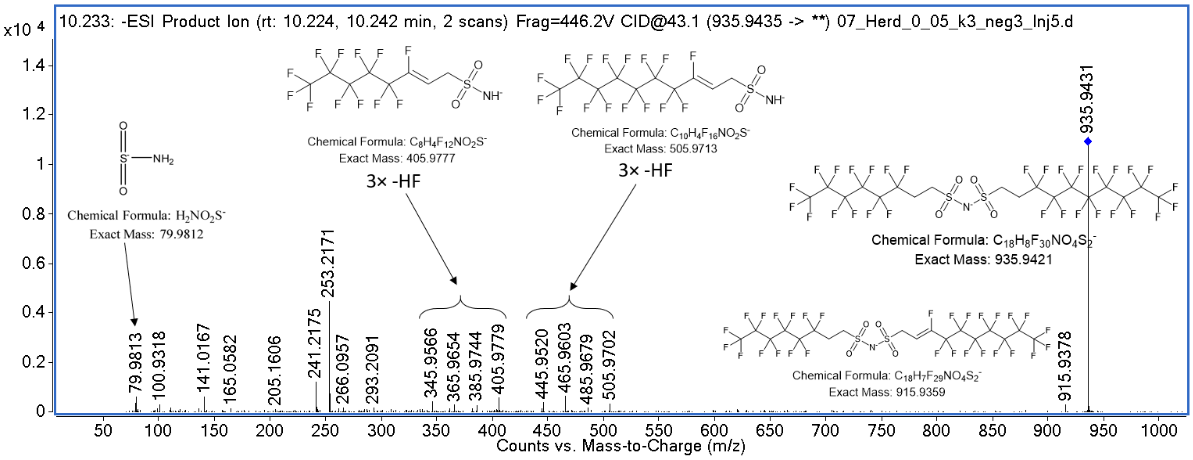


**Fig. S5c:** $MS^2$ spectrum ($ESI^-$, 43.1 eV, soil *S1*, 0 – 0.5 m, combined extract, iterative $MS^2$) of 6:2/8:2 FTSAm dimer (m/z 935.9421, 10.224 and 10.242 min).

# Substances detected for the first time in soil

### n:2 FTSAm-*N*-Me-*N*-PrAs (n = 6)

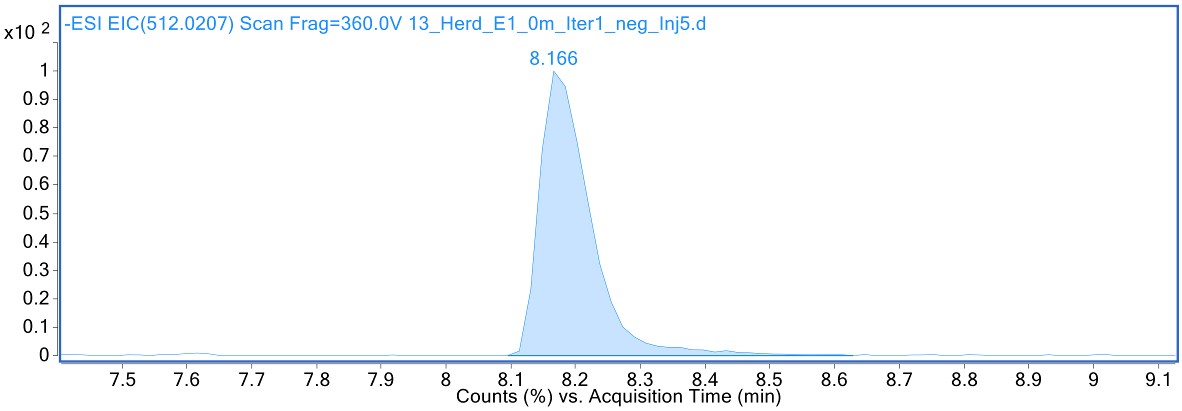


**Fig. S6a:** Chromatogram (ESI$^-$, soil *S1*, 0 – 0.5 m, first extract, iterative MS$^2$) of 6:2 FTSAm-*N*-Me-*N*-PrA (m/z 512.0207, 8.166 min).

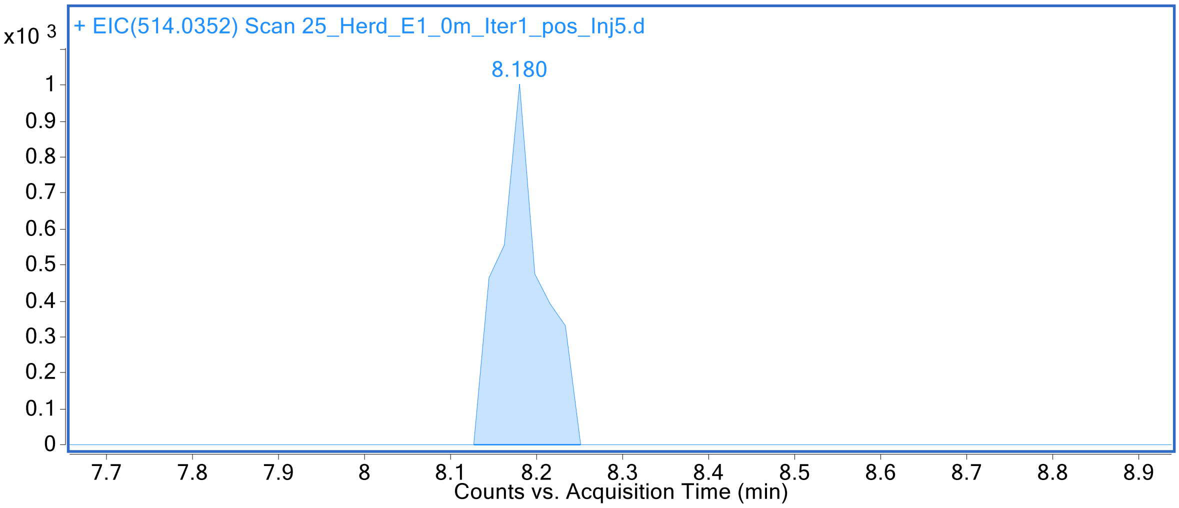


**Fig. S6b:** Chromatogram (ESI$^+$, soil *S1*, 0 – 0.5 m, first extract) of 6:2 FTSAm-*N*-Me-*N*-PrA (m/z 514.0352, 8.180 min).

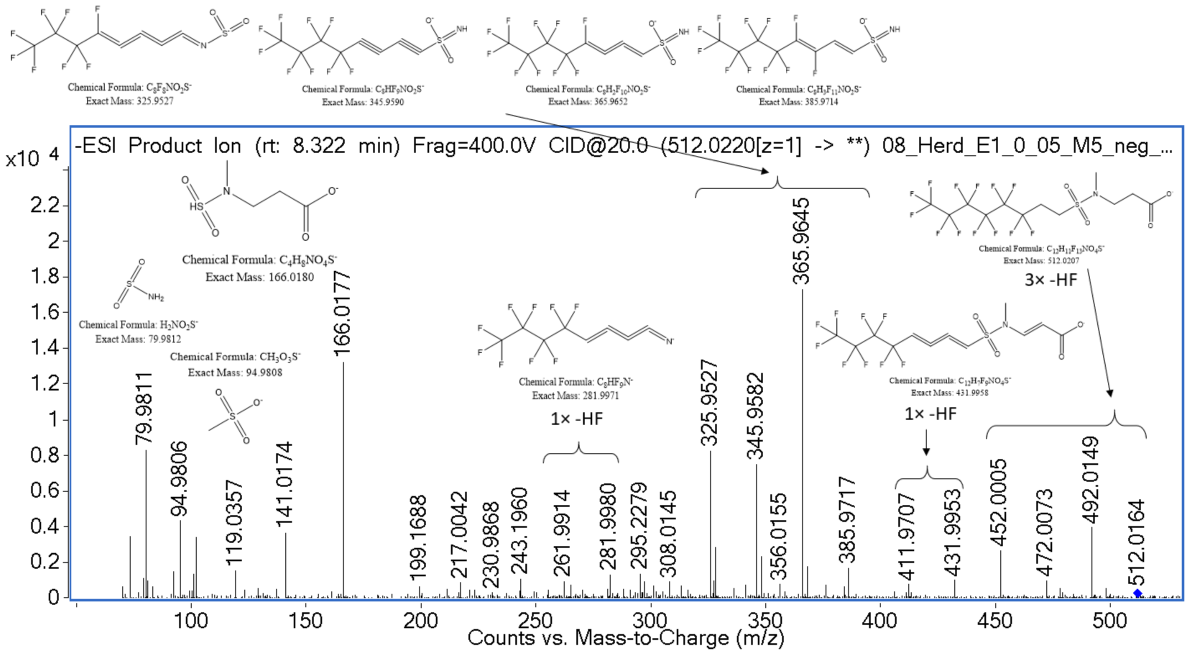


**Fig. S6c:** $MS^2$ spectrum (ESI$^-$, 20.0 eV, soil *S1*, 0 – 0.5 m, first extract, targeted $MS^2$) of 6:2 FTSAm-*N*-Me-*N*-PrA (m/z 512.0207, 8.322 min).

**n:2 FTSAm-U-Pr-DiMeAms (n = 6)**

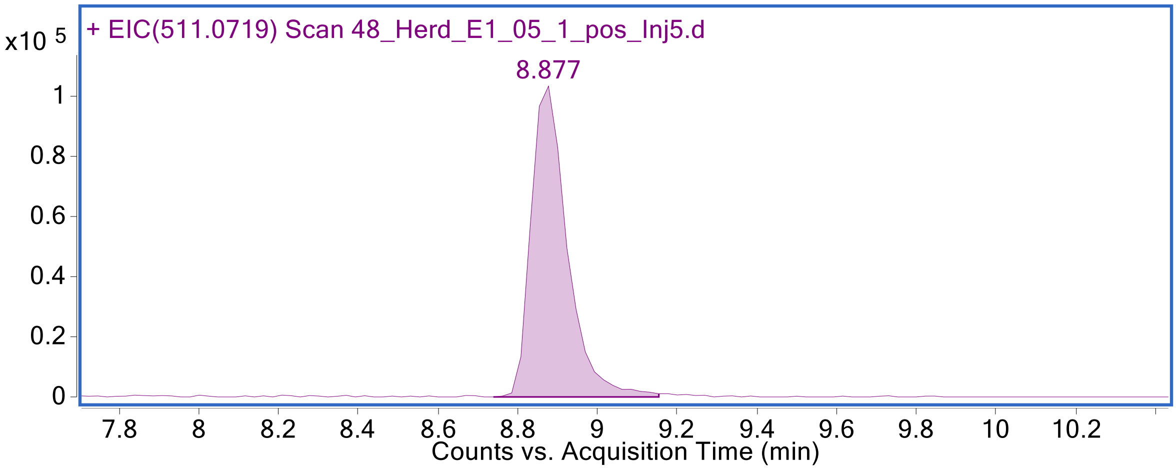


**Fig. S7a:** Chromatogram (ESI$^+$, soil *S1*, 0.5 – 1 m, first extract) of 6:2 FTSAm-U-Pr-DiMeAm (m/z 511.0719, 8.877 min).

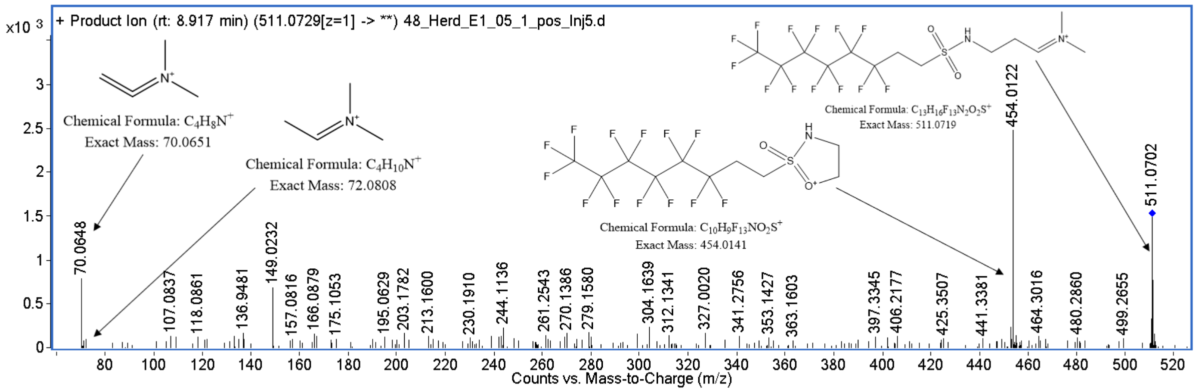


**Fig. S7b:** MS$^2$ spectrum (ESI$^+$, 30.3 eV, soil *S1*, 0.5 – 1 m, first extract) of 6:2 FTSAm-U-Pr-DiMeAm (m/z 511.0719, 8.917 min).

**n:2 FTSAm-PrAs (n = 6)**

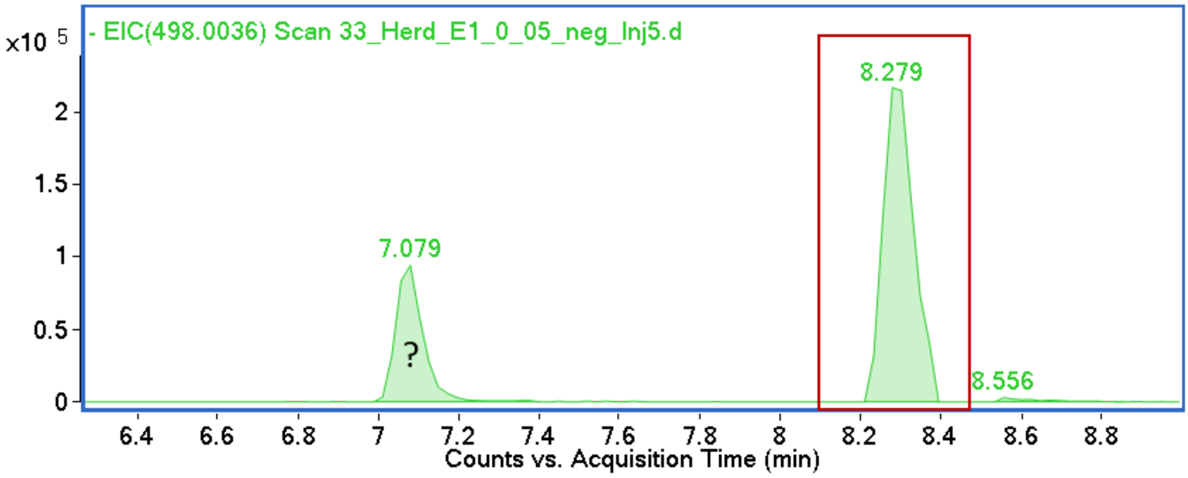


**Fig. S8a:** Chromatogram (ESI$^-$, soil *S1*, 0 – 0.5 m, first extract) of 6:2 FTSAm-PrA (m/z 498.0050, 8.279 min). First peak is possibly an isomer.

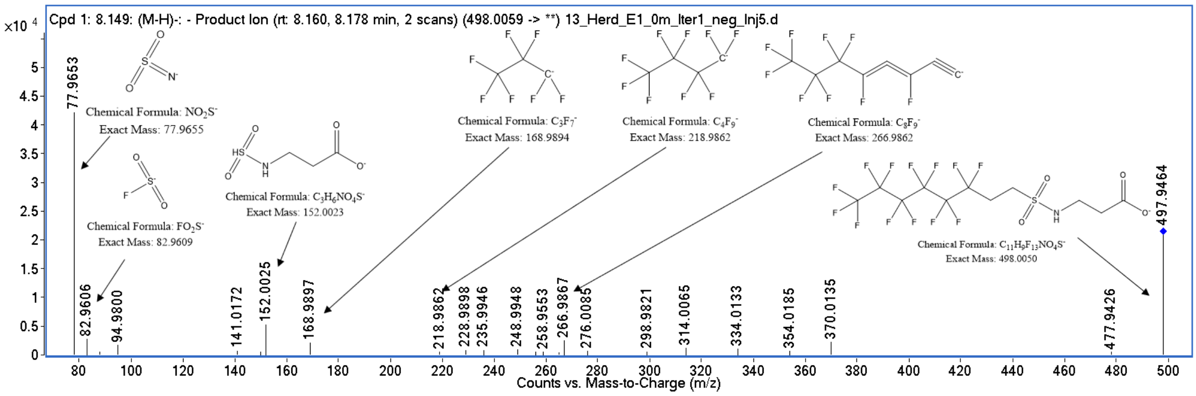


**Fig. S8b:** MS$^2$ spectrum (ESI$^-$, 29.9 eV, soil *S1*, 0 – 0.5 m, first extract, iterative MS$^2$) of 6:2 FTSAm-PrA (m/z 498.0050, 8.160 and 8.178 min).

**n:2 FTBs (n = 6, 8, 10, 12)**

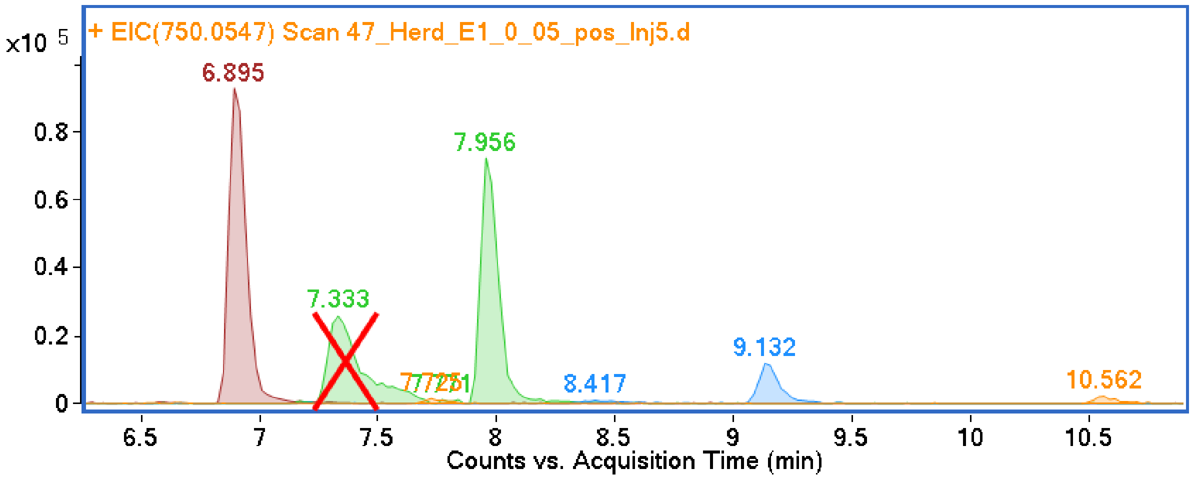


**Fig. S9a:** Chromatogram (ESI+, soil *S1*, 0 – 0.5 m, first extract) of n:2 FTBs (n = 6 (red, m/z 450.0733, 6.895 min), 8 (green, m/z 550.0669, 7.956 min), 10 (blue, m/z 650.0605, 9.132 min), and 12 (orange, m/z 750.0542, 10.562 min)). Note: The first peak of 8:2 FTB (green, 7.333 min) is crossed out as it belongs to the Na-adduct of 6:2 FTSy-(2')OHPr-TriMeAm.

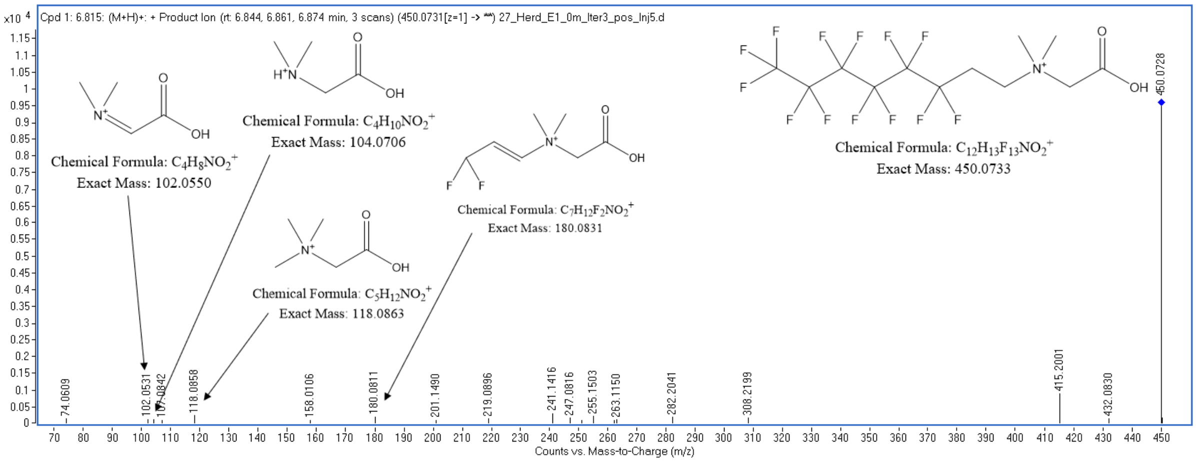


**Fig. S9b:** MS$^2$ spectrum (ESI+, 28.5 eV, soil *S1*, 0 – 0.5 m, first extract, iterative MS$^2$) of 6:2 FTB (m/z 450.0733, 6.844, 6.861, and 6.874 min).

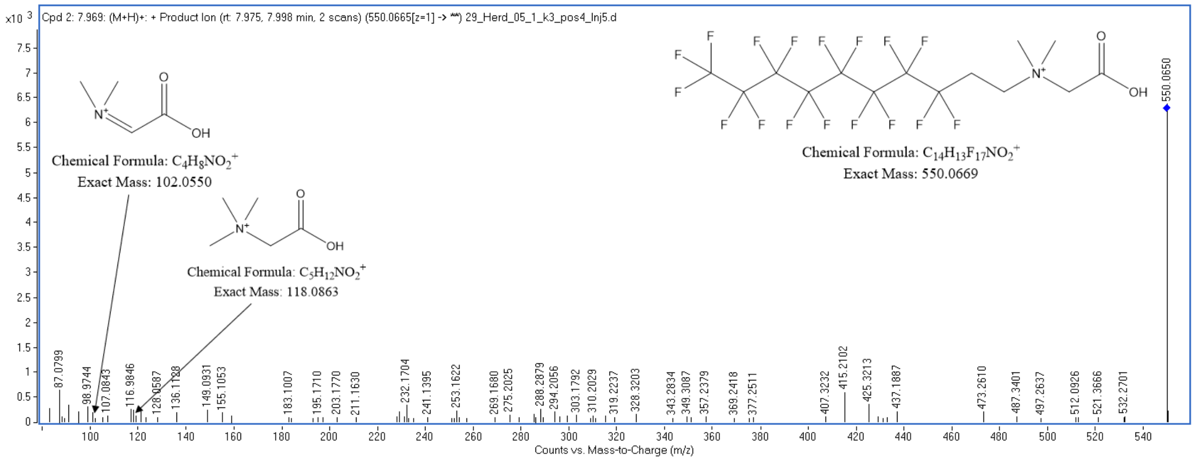


**Fig. S9c:** MS$^2$ spectrum (ESI+, 31.5 eV, soil *S1*, 0.5 – 1 m, combined extract, iterative MS$^2$) of 8:2 FTB (m/z 550.0669, 7.975 and 7.998 min).

**n:4 FTBs (n = 4, 6, 8, 10)**

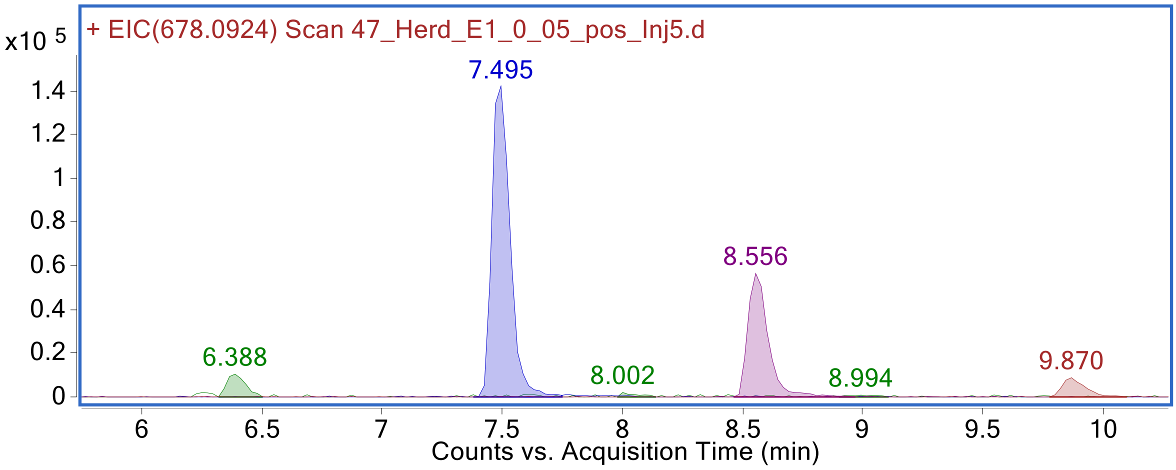


**Fig. S10a:** Chromatogram (ESI$^+$, soil *S1*, 0 – 0.5 m, first extract) of n:4 FTBs (n = 4 (green, m/z 378.1110, 6.388 min), 6 (blue, m/z 478.1046, 7.495 min), 8 (violet, m/z 578.0982, 8.556 min), and 10 (red, m/z 678.0918, 9.870 min)).

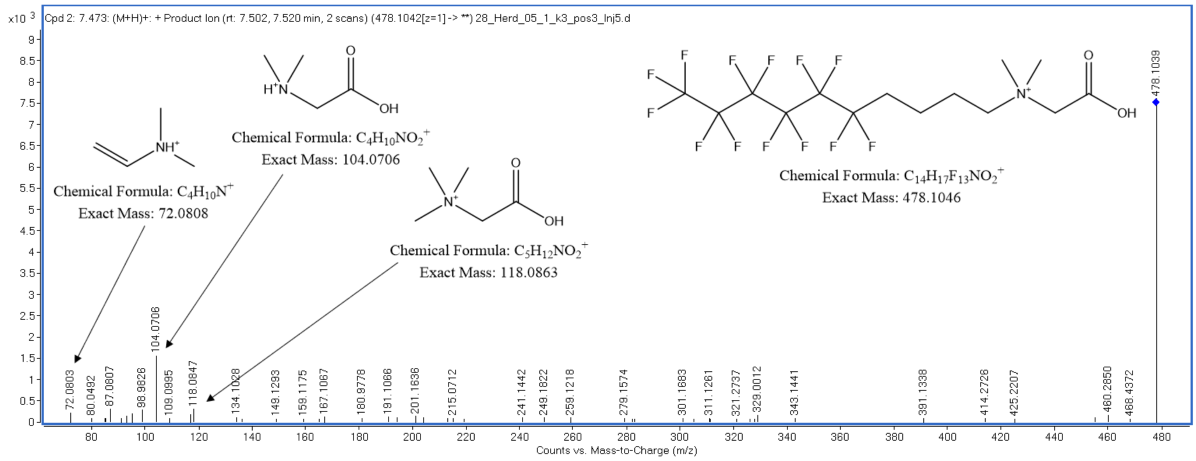


**Fig. S10b:** MS$^2$ spectrum (ESI$^+$, 29.3 eV, soil *S1*, 0.5 – 1 m, combined extract, iterative MS$^2$) of 6:4 FTB (m/z 478.1046, 7.502 and 7.520 min).

**n:1:3 FTBs (n = 5, 7, 9)**

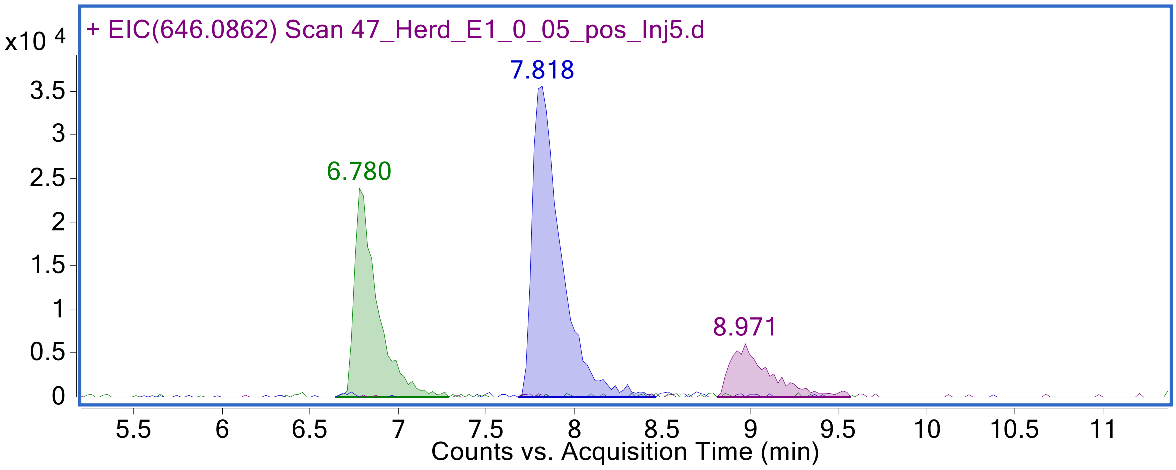


**Fig. S11a:** Chromatogram (ESI$^+$, soil *S1*, 0 – 0.5 m, first extract) of n:1:3 FTBs (n = 5 (green, m/z 446.0984, 6.780 min), 7 (blue, m/z 546.0920, 7.818 min), and 9 (violet, m/z 646.0856, 8.971 min)).

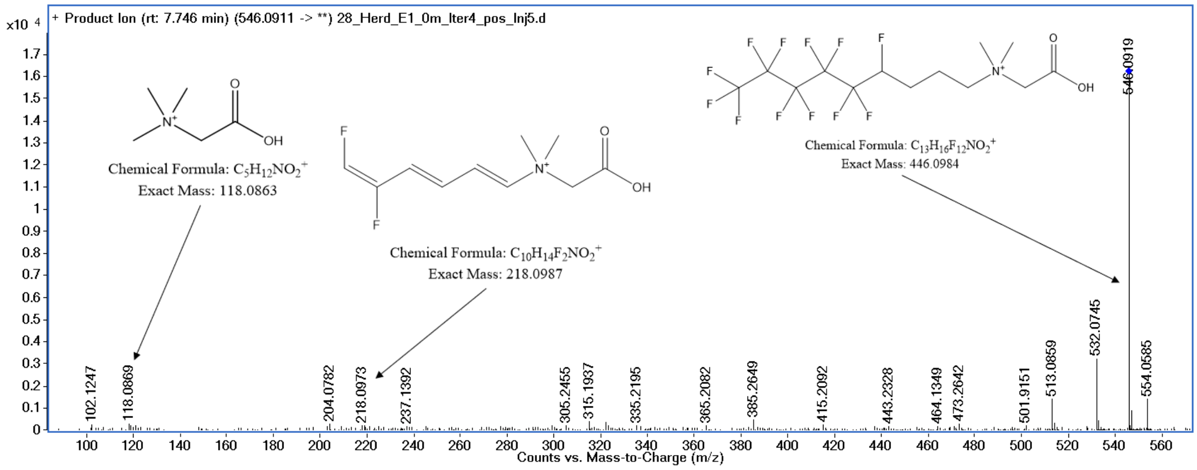


**Fig. S11b:** $MS^2$ spectrum ($ESI^+$, 28.4 eV, soil *S1*, 0 – 0.5 m, first extract, iterative $MS^2$) of 7:1:3 FTB (m/z 546.0920, 7.746 min).

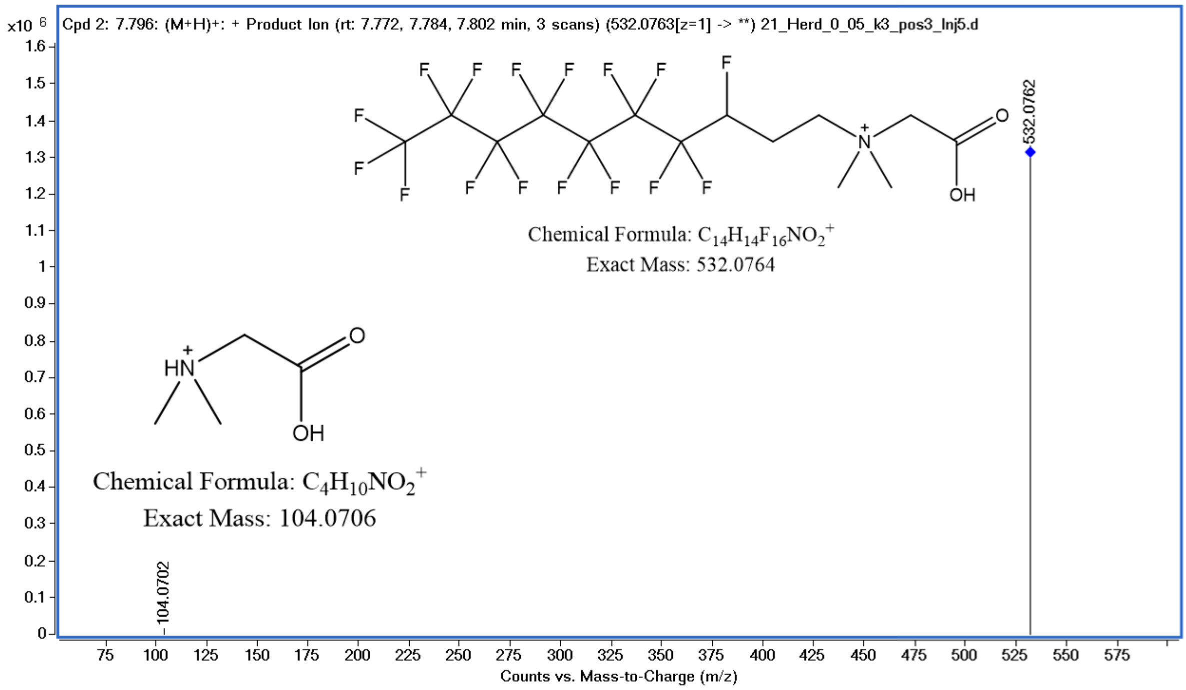


**Fig. S11d:** $MS^2$ spectrum ($ESI^+$, 31.0 eV, soil *S1*, 0 – 0.5 m, combined extract, iterative $MS^2$) of 7:1:2 FTB (m/z 532.0764, 7.772, 7.784 and 7.802 min).

**K-n:2 FTSAs (n = 6, 8, 10)**

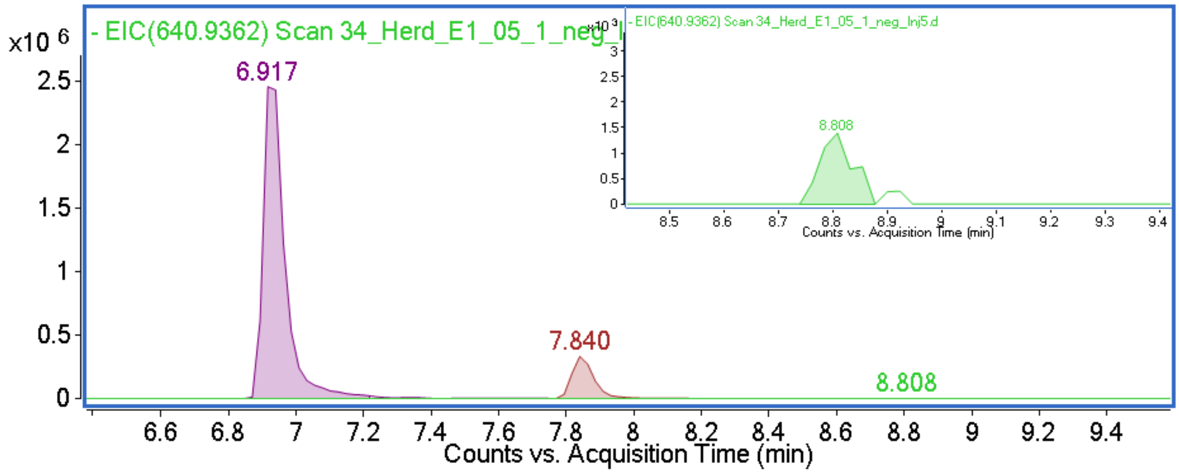


**Fig. S12a:** Chromatogram (ESI$^-$, soil *S1*, 0.5 – 1 m, first extract) of K-n:2 FTSAs (n = 6 (violet, m/z 440.9472, 6.917 min), 8 (red, m/z 540.9408, 7.840 min), and 10 (green, m/z 640.9344, 8.808 min)). Scale-up in top right corner.

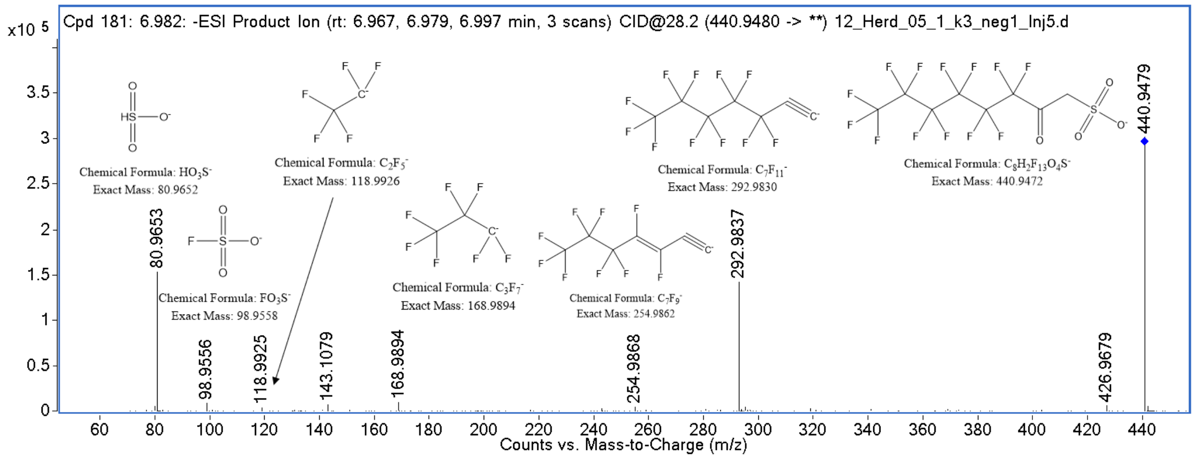


**Fig. S12b:** MS$^2$ spectrum (ESI$^-$, 28.2 eV, soil *S1*, 0.5 – 1 m, combined extract, iterative MS$^2$) of K-6:2 FTSA (m/z 440.9472, 6.967, 6.979, and 6.997 min).

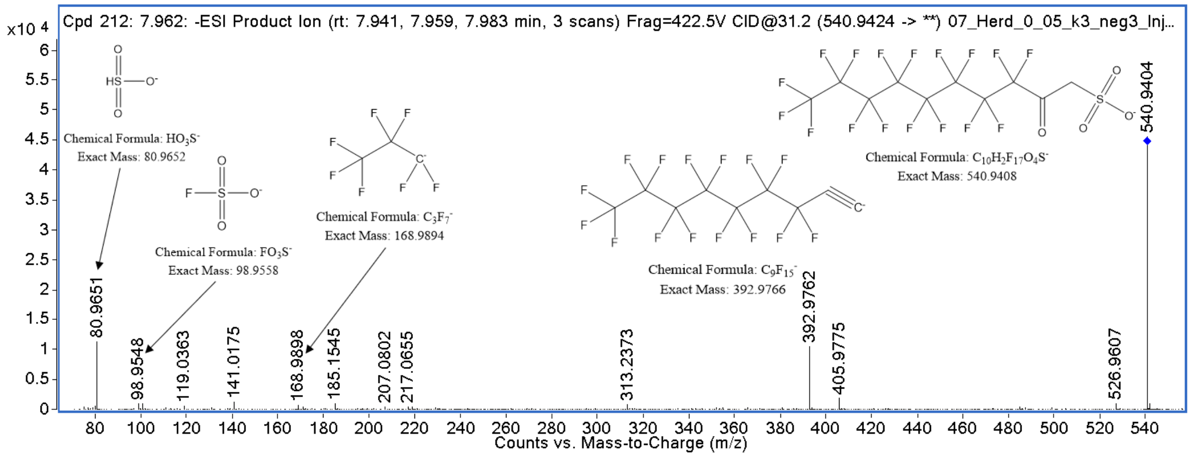


**Fig. S12c:** MS$^2$ spectrum (ESI$^-$, 31.2 eV, soil *S1*, 0 – 0.5 m, combined extract, iterative MS$^2$) of K-8:2 FTSA (m/z 540.9408, 7.941, 7.959, and 7.983 min).

## U-n:2 FTSAs (n = 6, 8)

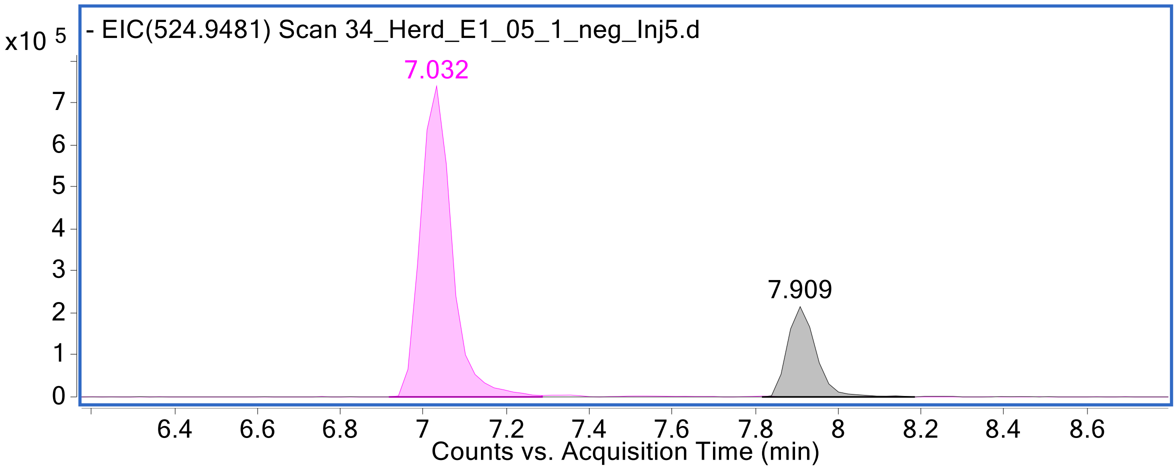


**Fig. S13a:** Chromatogram (ESI$^-$, soil *S1*, 0.5 – 1 m, first extract) of U-n:2 FTSAs (n = 6 (pink, m/z 424.9523, 7.032 min) and 8 (grey, m/z 524.9459, 7.909 min)).

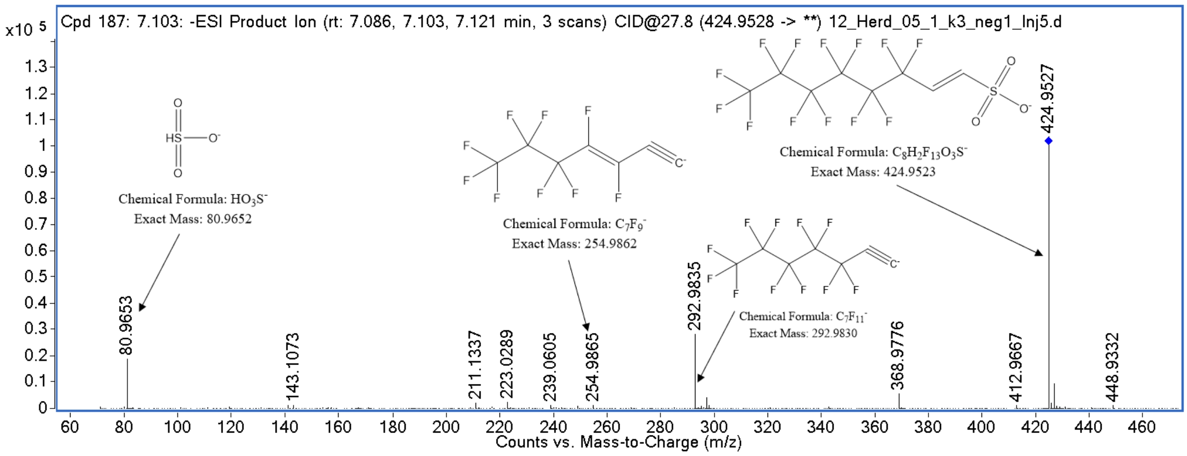


**Fig. S13b:** MS$^2$ spectrum (ESI$^-$, 27.8 eV, soil *S1*, 0.5 – 1 m, combined extract, iterative MS$^2$) of U-6:2 FTSA (m/z 424.9523, 7.086, 7.103, and 7.121 min).

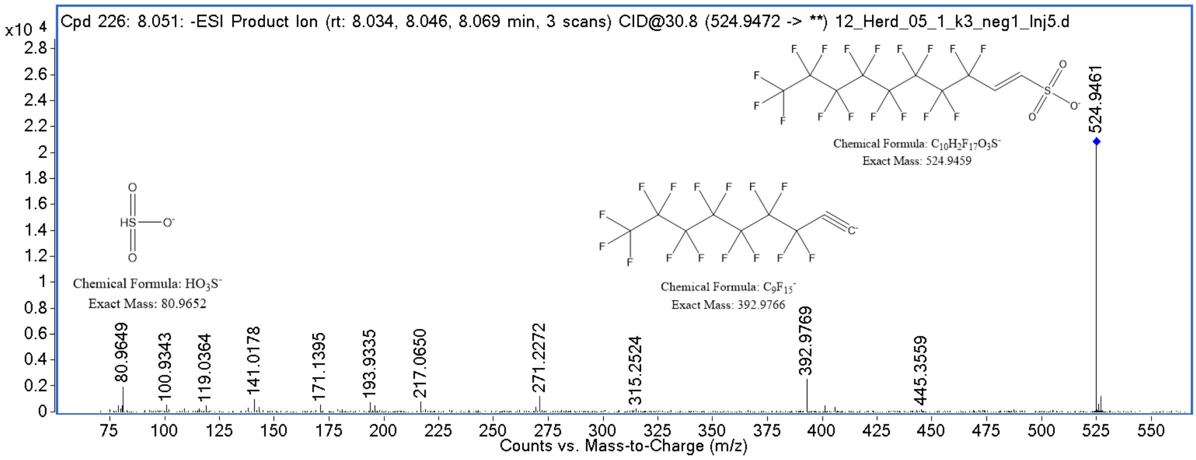


**Fig. S13c:** MS$^2$ spectrum (ESI$^-$, 30.8 eV, soil *S1*, 0.5 – 1 m, combined extract, iterative MS$^2$) of U-8:2 FTSA (m/z 524.9459, 8.034, 8.046, and 8.069 min).

**n:2 FTSy-(2')OHPr-TriMeAms (n = 4, 6, 8)**

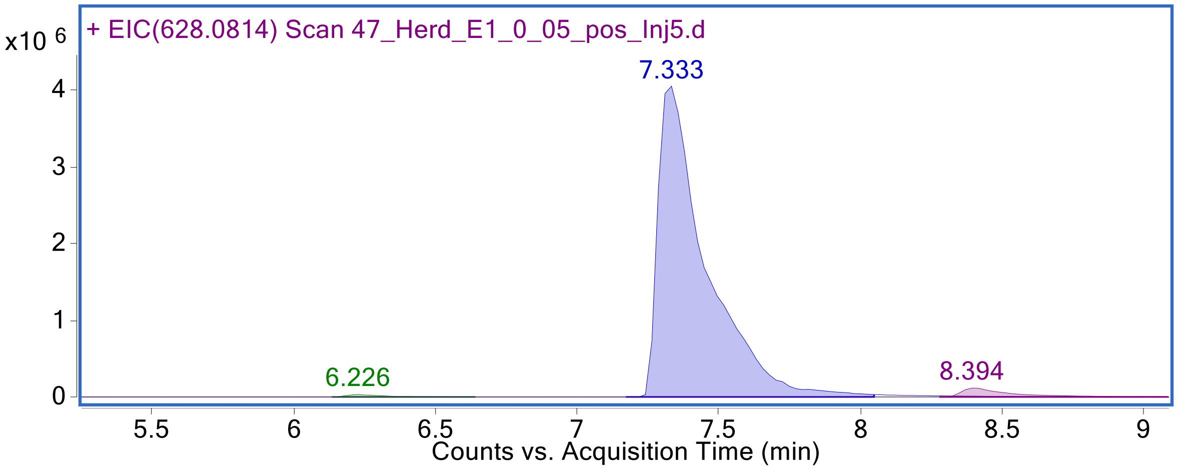


**Fig. S14a:** Chromatogram (ESI$^+$, soil *S1*, 0 – 0.5 m, first extract) of n:2 FTSy-(2')OHPr-TriMeAms (n = 4 (green, m/z 428.0936, 6.226 min), 6 (blue, m/z 528.0873, 7.333 min), and 8 (violet, m/z 628.0809, 8.394 min)).

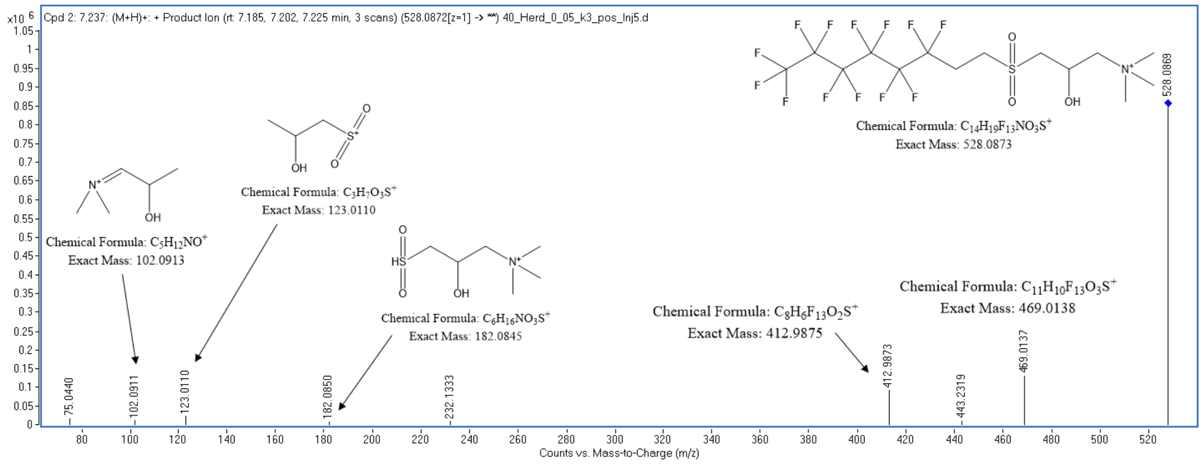


**Fig. S14b:** MS$^2$ spectrum (ESI$^+$, 30.8 eV, soil *S1*, 0 – 0.5 m, combined extract) of 6:2 FTSy-(2')OHPr-TriMeAm (m/z 528.0873, 7.185, 7.202, and 7.225 min).

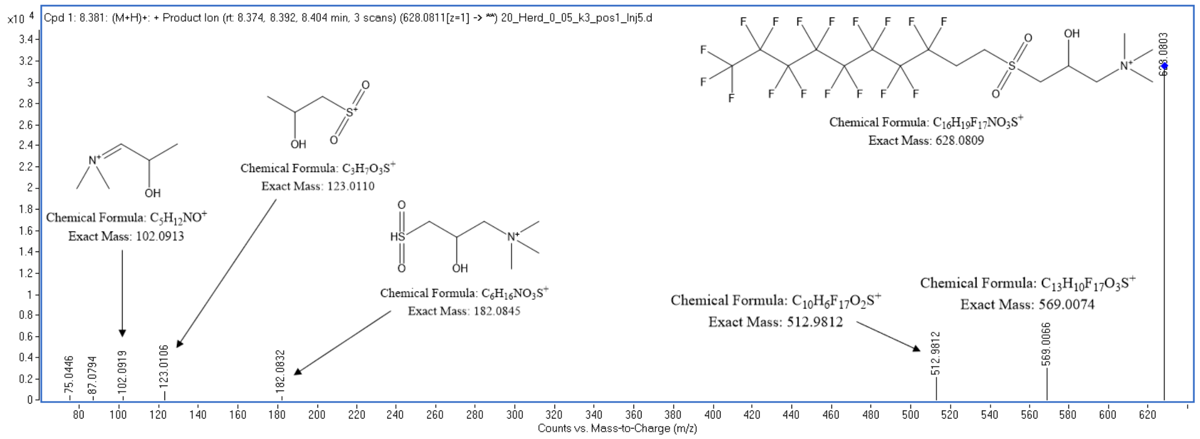


**Fig. S14c:** MS$^2$ spectrum mode (ESI$^+$, 33.8 eV, soil *S1*, 0 – 0.5 m, combined extract, iterative MS$^2$) of 8:2 FTSy-(2')OHPr-TriMeAm (m/z 628.0809, 8.374, 8.392, and 8.404 min).

# Previously identified Substances

## 1. PFCA, PFSA, FTCA, FTSA

### PFCAs (n = 3 - 11)

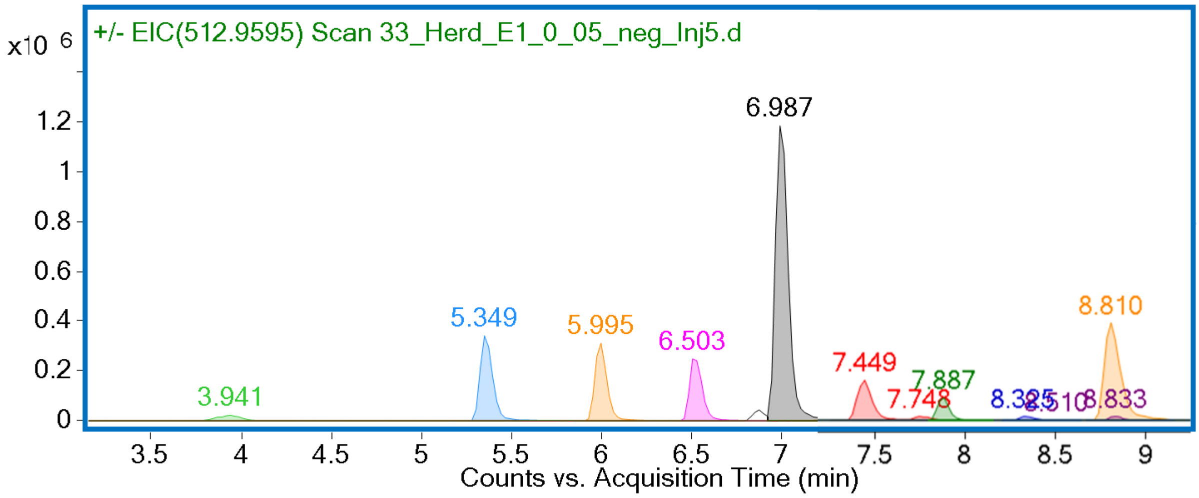


**Fig. S15:** Chromatogram (ESI$^-$, soil *S1*, 0 – 0.5 m, first extract) of PFCAs (n = 3 (green, m/z 212.9792, 3.941 min), 4 (blue, m/z 262.9760, 5.349 min), 5 (orange, m/z 312.9728, 5.995 min), 6 (pink, m/z 362.9696, 6.503 min), 7 (grey, m/z 412.9664, 6.987 min), 8 (red, m/z 462.9632, 7.449 min), 9 (dark green, m/z 512.9600, 7.887 min), 10 (dark blue, m/z 562.9568, 8.325 min), and 11 (violet, m/z 612.9537, 8.833 min)). Note: PFHxA (orange) shows a second peak at a retention time of 8.810 min. No MS$^2$ spectra are shown as fragmentation of PFCAs is already well known.

### PFSAs (n = 3-13)

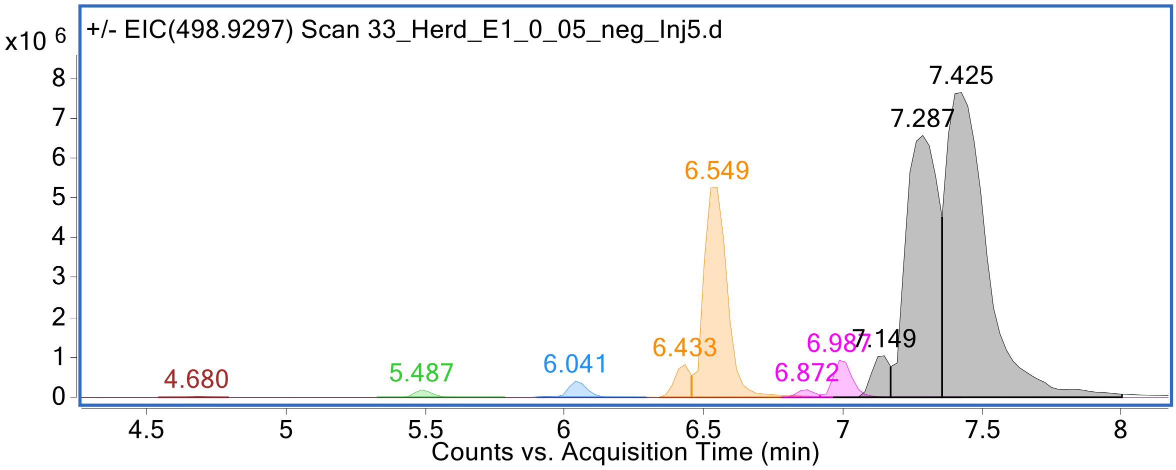


**Fig. S16a:** Chromatogram (ESI$^-$, soil *S1*, 0 – 0.5 m, first extract) of PFSAs (n = 3 – 8; n = 3 (dark red, m/z 248.9462, 4.680 min), 4 (green, m/z 298.9430, 5.487 min), 5 (blue, m/z 348.9398, 6.041 min), 6 (orange, m/z 398.9366, 6.433 and 6.549 min), 7 (pink, m/z 448.9334, 6.872 and 6.987 min), and 8 (grey, m/z 498.9302, 7.149, 7.287, and 7.425 min)). Note: The PFOS (n = 8) peak is in saturation. No MS$^2$ spectra are shown as fragmentation of PFSAs is already well known.

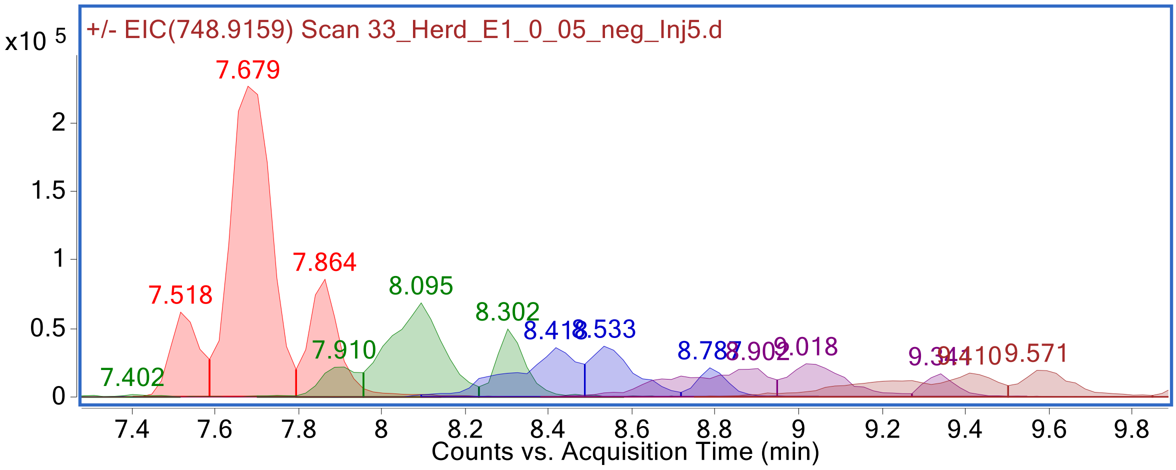


**Fig. S16b:** Chromatogram (ESI$^-$, soil *S1*, 0 – 0.5 m, first extract). of PFSAs (n = 9 – 13); n = 9 (red, m/z 548.9270, 7.518, 7.679, and 7.864 min), 10 (dark green, m/z 598.9238, 7.910, 8.095, and 8.302 min), 11 (dark blue, m/z 648.9206, 8.418, 8.533, and 8.787 min), 12 (violet, m/z 698.9174, 8.902, 9.018, and 9.341 min), and 13 (dark red, m/z 748.9143, 9.419 and 9.571 min)).

**n:3 FTCAs (n = 3 - 9)**

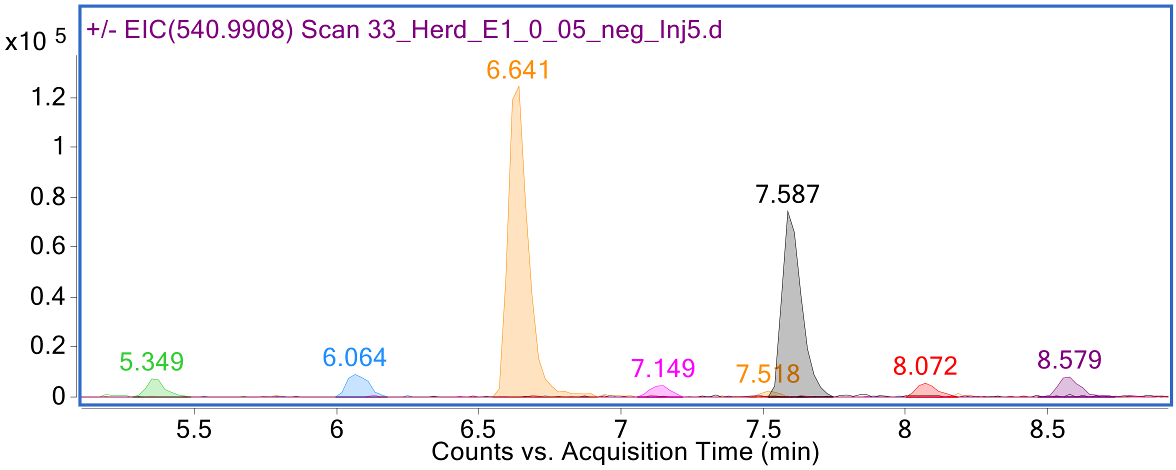


**Fig. S17:** Chromatogram (ESI$^-$, soil *S1*, 0 – 0.5 m, first extract) of n:3 FTCAs (n = 3 (green, m/z 241.0105, 5.349 min), 4 (blue, m/z 291.0073, 6.064 min), 5 (orange, m/z 341.0041, 6.641 min), 6 (pink, m/z 391.0009, 7.149 min), 7 (gray, m/z 440.9977, 7.587 min), 8 (red, m/z 490.9945, 8.072 min), and 9 (violet, m/z 540.9913, 8.579 min)).

**n:2 FTSAs (n = 6, 8, 10, 12, 14)**

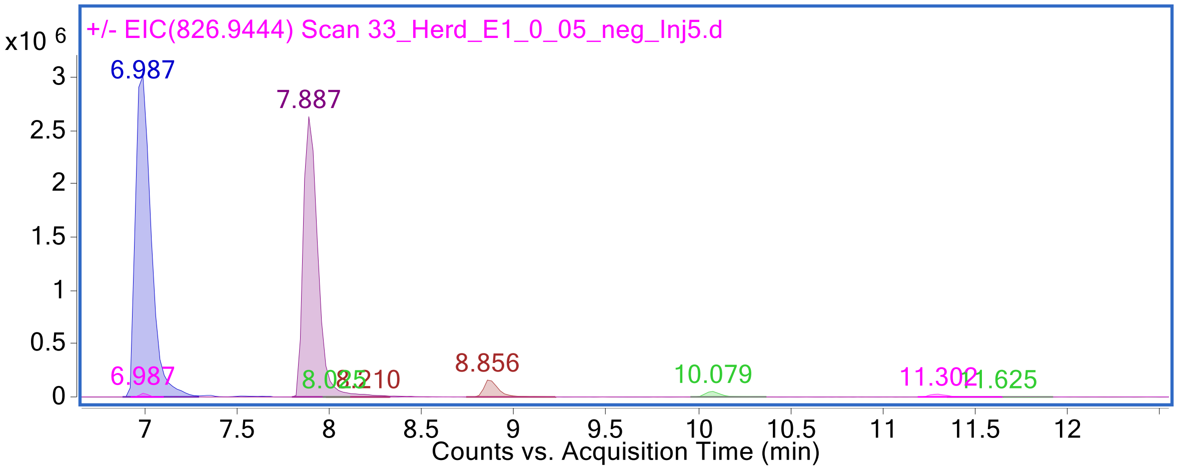


**Fig. S18:** Chromatogram (ESI-, soil *S1*, 0 – 0.5 m, first extract) of n:2 FTSAs (n = 6 (blue, m/z 426.9679, 6.987 min), 8 (violet, m/z 526.9615, 7.887 min), 10 (red, m/z 626.9551, 8.856 min), 12 (green, m/z 726.9487, 10.079 min), and 14 (pink, m/z 826.9424, 11.302 min)).

## 2. PFSA- and U-PFSA derivatives

### Cl-PFSAs (n = 6, 8)

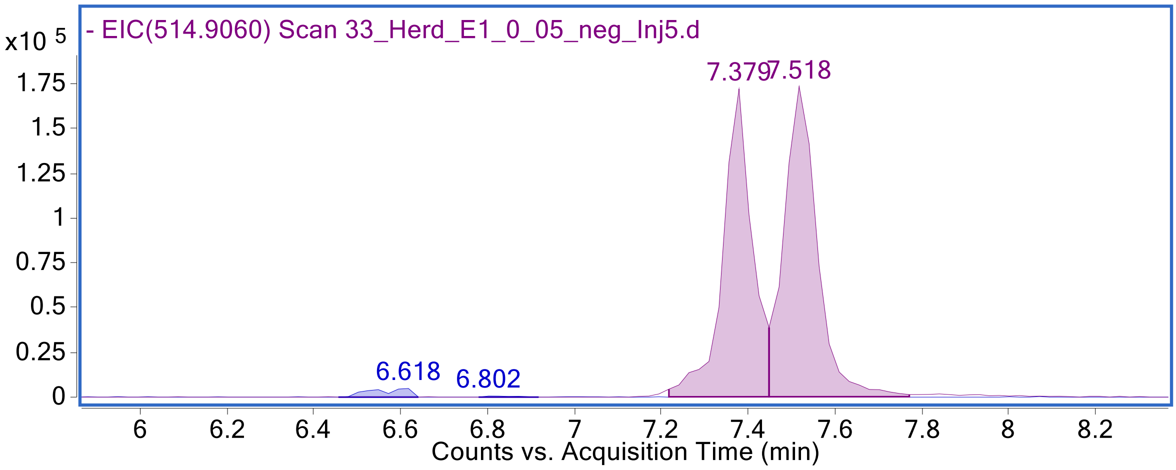


**Fig. S19a:** Chromatogram (ESI$^-$, soil *S1*, 0 – 0.5 m, first extract) of Cl-PFSAs (n = 6 (blue, m/z 414.9071, 6.618 and 6.802 min) and 8 (violet, m/z 514.9007, 7.379 and 7.518 min)).

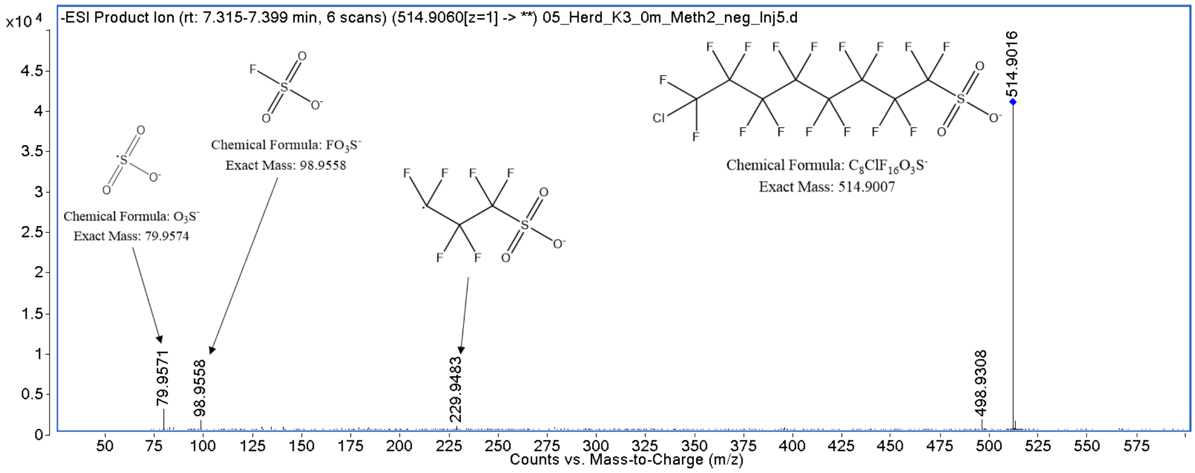


**Fig. S19b:** MS$^2$ spectrum (ESI$^-$, different CEs (10 – 40 eV), soil *S1*, 0 – 0.5 m, combined extract, targeted MS$^2$) of (8)Cl-PFOS (m/z 514.9007, 7.315 – 7.399 min).

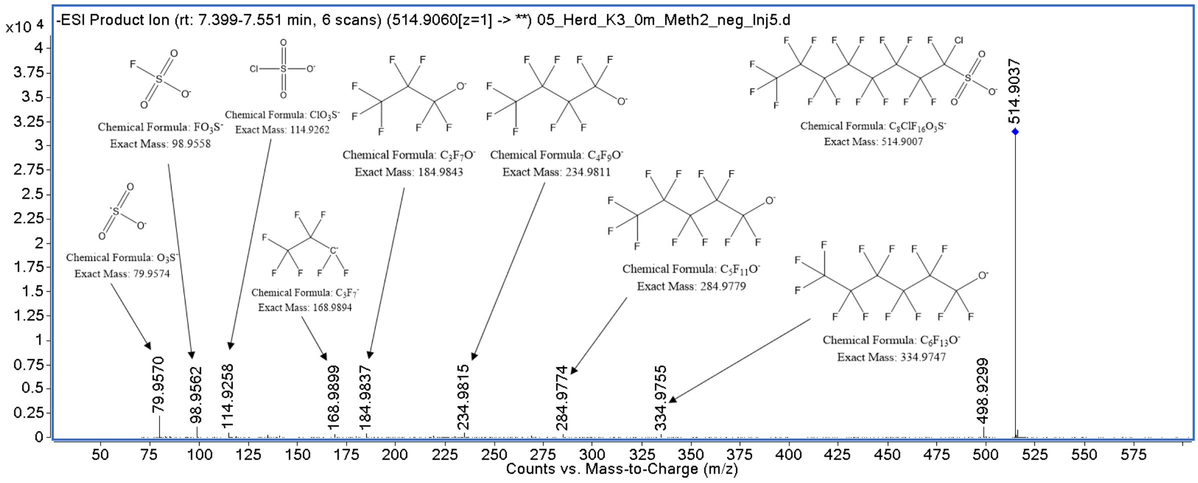


**Fig. S19c:** MS$^2$ spectrum (ESI$^-$, different CEs (10 – 40 eV), soil *S1*, 0 – 0.5 m, combined extract, targeted MS$^2$) of (1)Cl-PFOS (m/z 514.9007, 7.399 – 7.551 min).

## $SF_5$-PFSAs (n = 7 - 10)

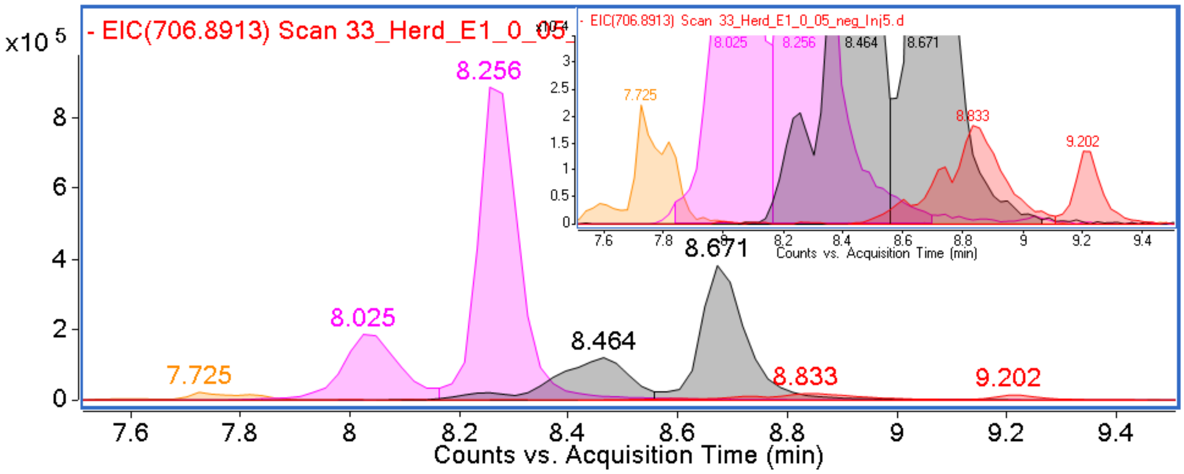

**Fig. S20a:** Chromatogram (ESI$^-$, soil *S1*, 0 – 0.5 m, first extract) of $SF_5$-PFSAs (n = 7 (orange, m/z 556.8991, 7.725 min), 8 (pink, m/z 606.8959, 8.025 and 8.256 min), 9 (grey, m/z 656.8927, 8.464 and 8.671 min), and 10 (red, m/z 706.8895, 8.833 nad 9.202 min)). Scale-up in top right corner.

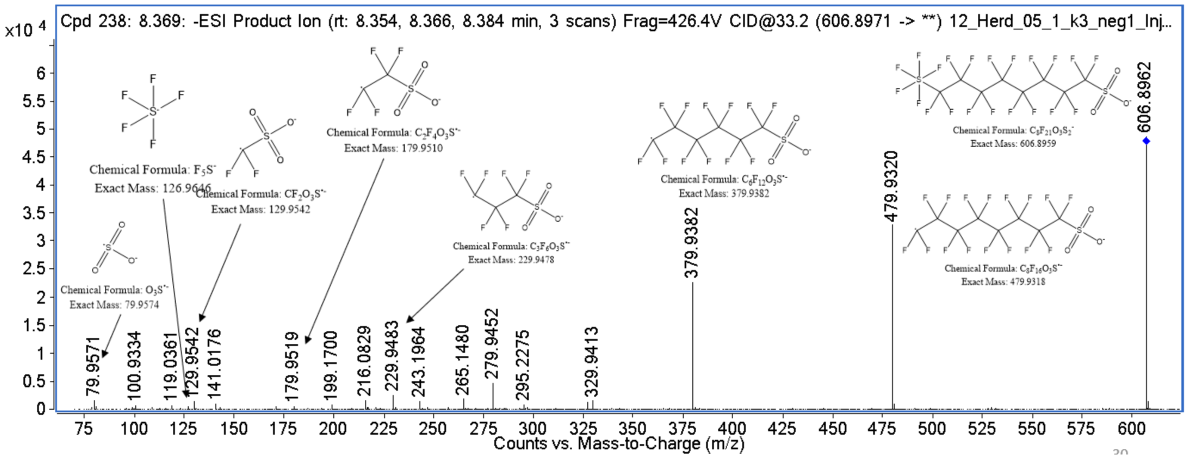

**Fig. S20b:** $MS^2$ spectrum (ESI$^-$, 33.2 eV, soil *S1*, 0.5 – 1 m, combined extract, iterative $MS^2$) of $SF_5$-PFOS (m/z 606.8959, 8.354, 8.366, and 8.384 min).

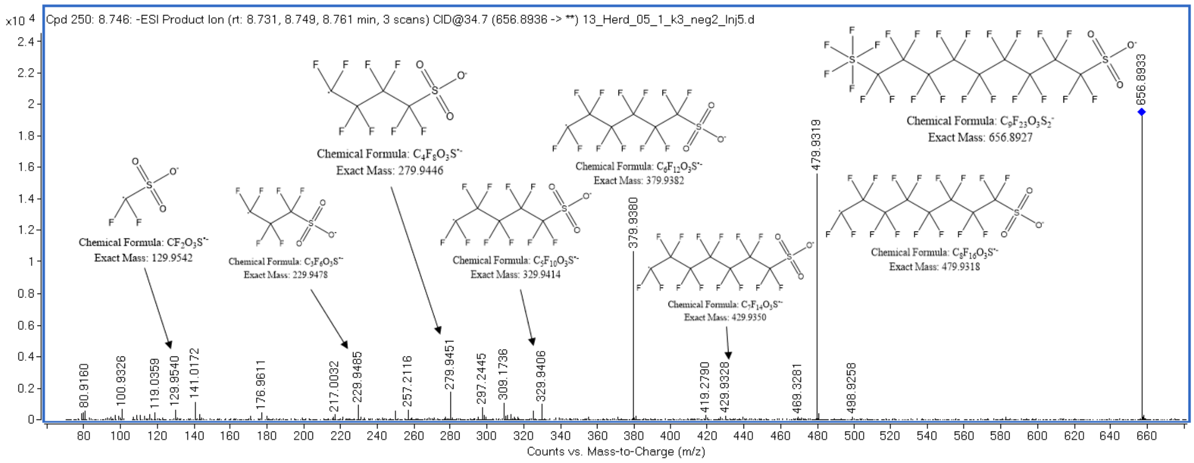

**Fig. S20c:** $MS^2$ spectrum (ESI$^-$, 34.7 eV, soil *S1*, 0.5 – 1 m, combined extract, iterative $MS^2$) of $SF_5$-PFNS (m/z 656.8927).

## H-PFSAs (n = 8, 10)

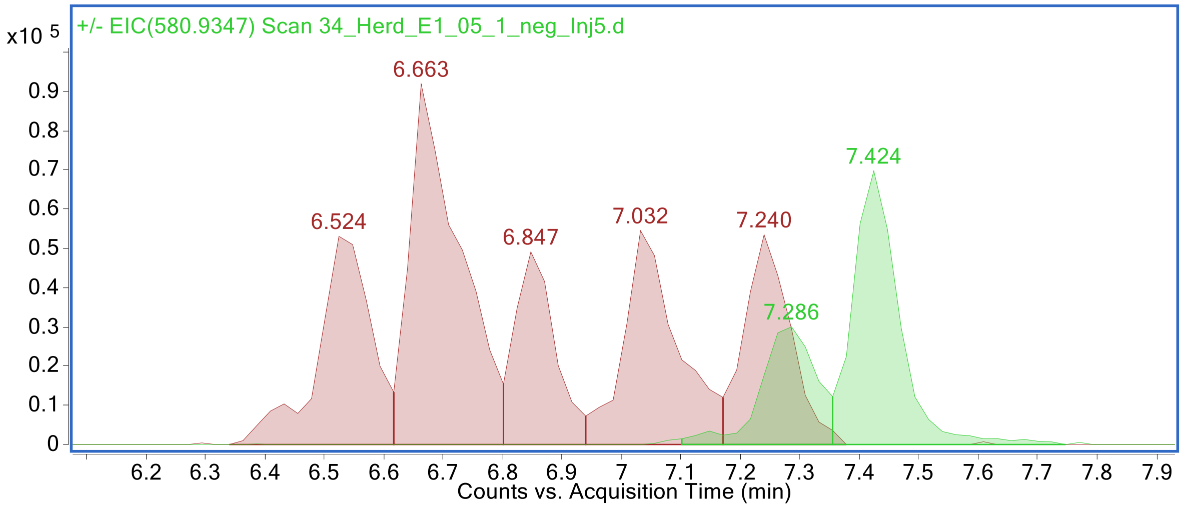


**Fig. S21a:** Chromatogram (ESI$^-$, soil *S1*, 0.5 – 1 m, first extract) of H-PFSAs (n = 8 (red, m/z 480.9396, 6.524, 6.663, 6.847, 7.032, and 7.240 min) and 10 (green, m/z 580.9333, 7.286 and 7.424 min)).

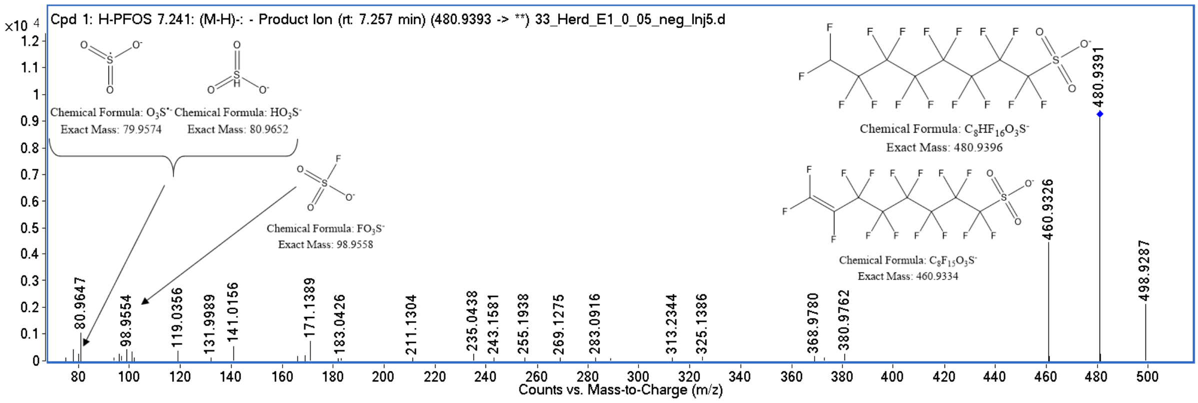


**Fig. 21b:** MS$^2$ spectrum (ESI$^-$, 29.4 eV, soil *S1*, 0 – 0.5 m, first extract) of H-PFOS (m/z 480.9396, 7.257 min).

**K-PFSAs/U-E-PFSAs (n = 6/7, 7/8, 8/9, 9/10, 10/11, 11/12, 12/13, 13/14, 14/15)**

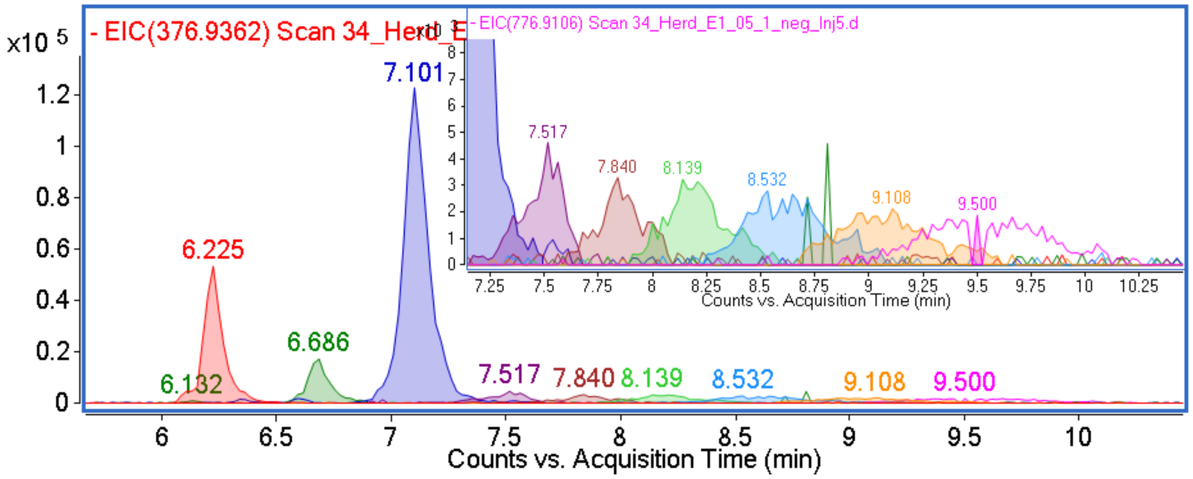


**Fig. S22a:** Chromatogram (ESI$^-$, soil *S1*, 0.5 – 1 m, first extract) of K-PFSAs/U-E-PFSAs (n = 6/7 (red, m/z 376.9347, 6.225 min), 7/8 (dark green, m/z 426.9315, 6.686 min), 8/9 (dark blue, m/z 476.9283, 7.101 min), 9/10 (violet, m/z 526.9251, 7.517 min), 10/11 (dark red, m/z 576.9219, 7.840 min), 11/12 (green, m/z 626.9187, 8.139 min), 12/13 (blue, m/z 676.9156, 8.532 min), 13/14 (orange, m/z 726.9124, 9.108 min), and 14/15 (pink, m/z 776.9092, 9.500 min)). Scale-up in top right corner.

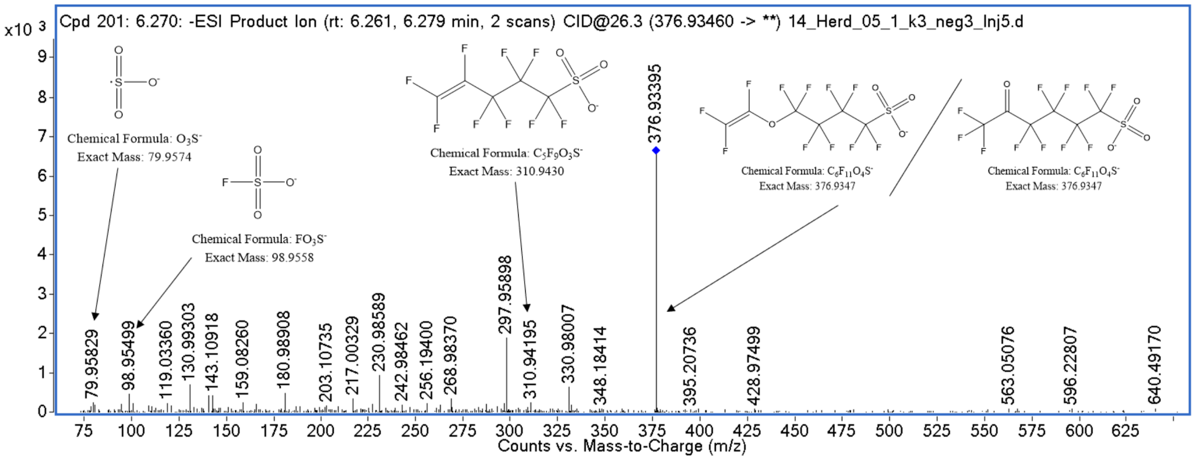


**Fig. S22b:** MS$^2$ spectrum (ESI$^-$, 26.3 eV, soil *S1*, 0.5 – 1 m, combined extract, iterative MS$^2$) of U-E-PFHpS/K-PFHxS (m/z 376.9347, 6.261 and 6.279 min).

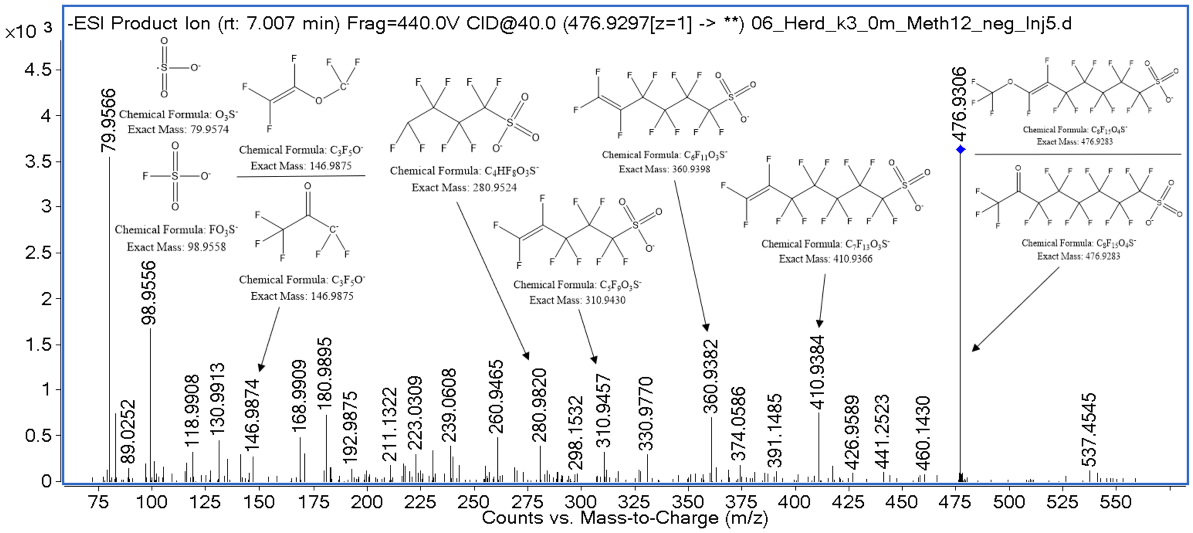

**Fig. S22c:** MS[2] spectrum (ESI$^-$, 40.0 eV, soil *S1*, 0 – 0.5 m, combined extract, targeted MS[2]) of U-E-PFNS/K-PFOS (m/z 476.9283, 7.007 min).

**PFASyAs (n = 8)**

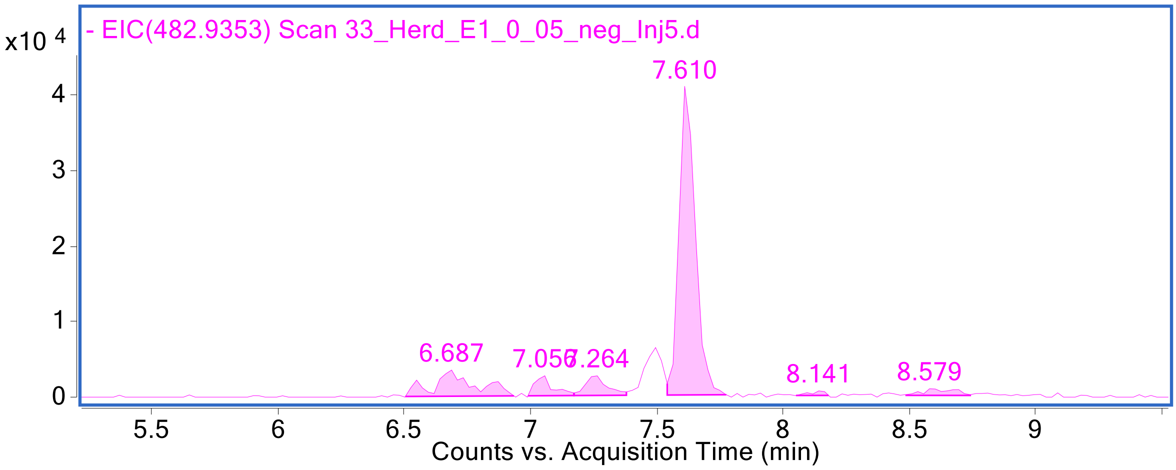

**Fig. S23a:** Chromatogram (ESI$^-$, soil *S1*, 0 – 0.5 m, first extract) of PFOSyA (m/z 482.9353, 7.610 min).

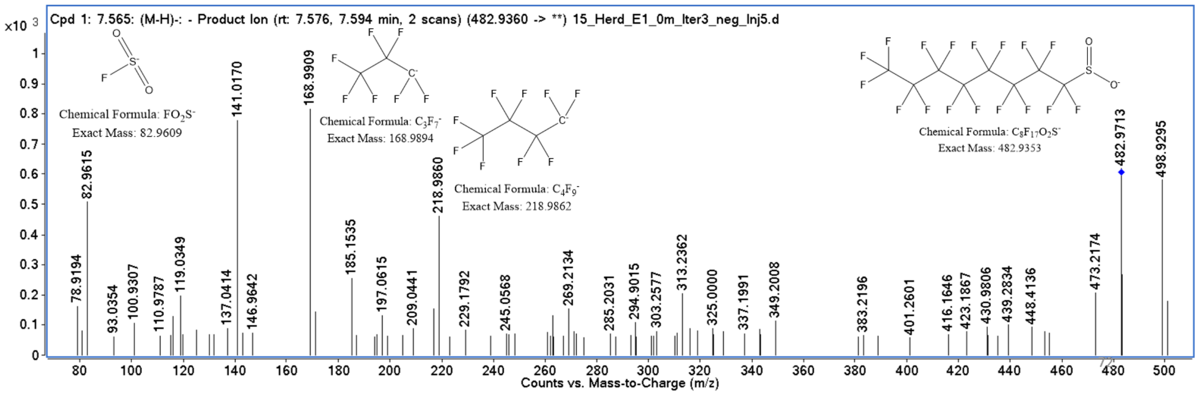

**Fig. S23b:** MS[2] spectrum (ESI$^-$, 29.5 eV, soil *S1*, 0 – 0.5 m, first extract, iterative MS[2]) of PFOSyA (m/z 482.9353, 7.576 and 7.594 min).

## U-PFSAs (n = 6 - 15)

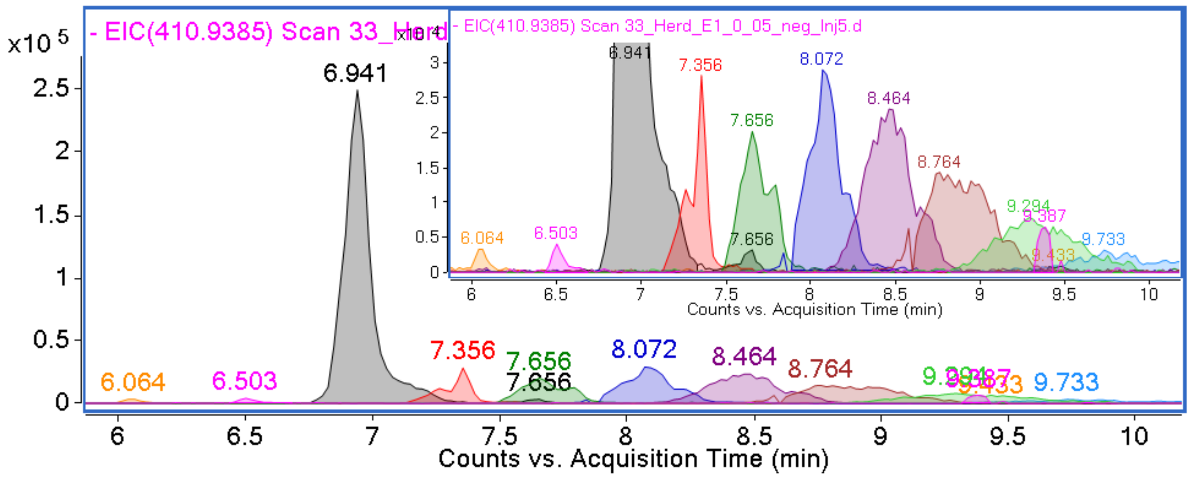


**Fig. S24a:** Chromatogram (ESI$^-$, soil *S1*, 0 – 0.5 m, first extract) of U-PFSAs (n = 6 (orange, m/z 360.9398, 6.064 min), 7 (pink, m/z 410.9366, 6.503 min), 8 (grey, m/z 460.9334, 6.941 min), 9 (red, m/z 510.9302, 7.356 min), 10 (dark green, m/z 560.9270, 7.656 min), 11 (dark blue, m/z 610.9238, 8.072 min), 12 (violet, m/z 660.9206, 8.464 min), 13 (dark red, m/z 710.9174, 8.764 min), 14 (green, m/z 760.9143, 9.294 min), and 15 (blue, m/z 810.9111, 9.733 min)). Scale-up in top right corner.

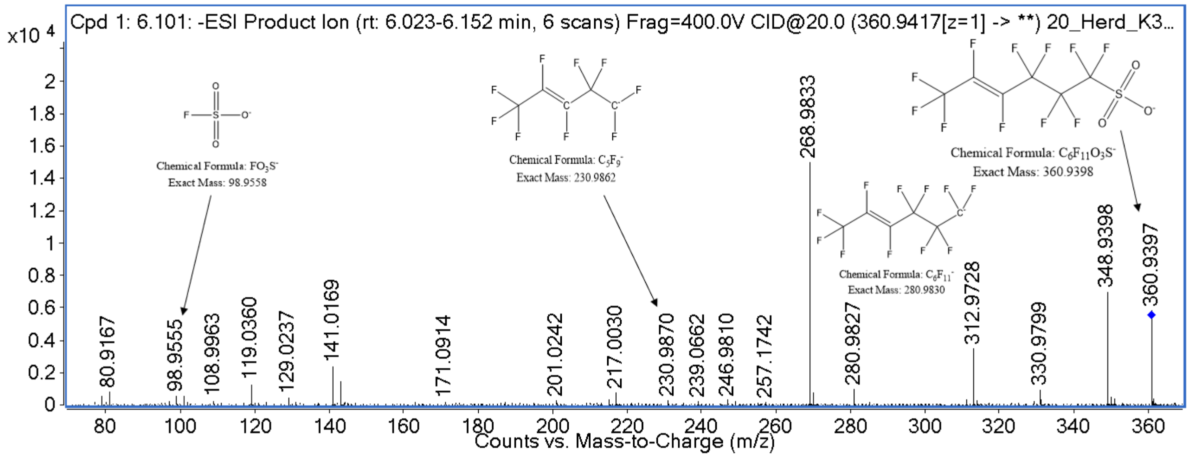


**Fig. S24b:** MS$^2$ spectrum (ESI$^-$, 20.0 eV, soil *S1*, 1.5 – 2 m, combined extract, targeted MS$^2$) of U-PFHxS (m/z 360.9398, 6.023 – 6.152 min).

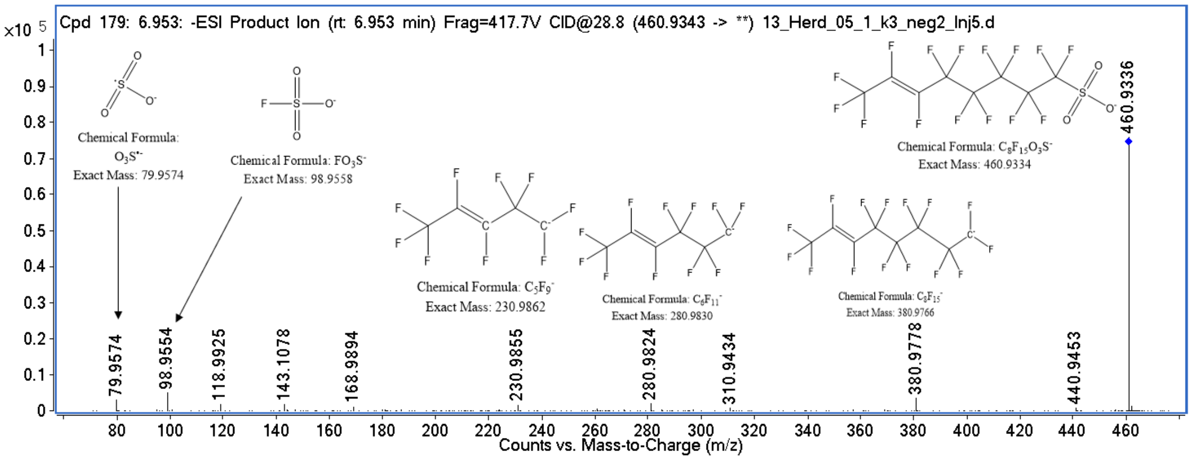


**Fig. S24c:** MS$^2$ spectrum (ESI$^-$, 28.8 eV, soil *S1*, 0.5 – 1 m, combined extract, iterative MS$^2$) of U-PFOS (m/z 460.9334, 6.953 min).

## $SF_5$-U-PFSAs (n = 8, 9, 10)

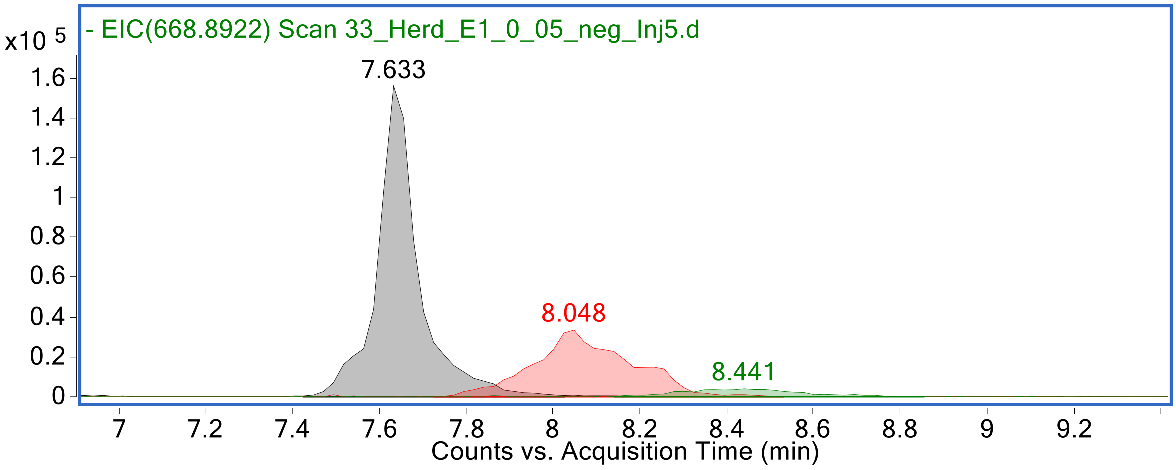


**Fig. S25a:** Chromatogram (ESI$^-$, soil *S1*, 0 – 0.5 m, first extract) of $SF_5$-U-PFSAs (n = 8 (grey, m/z 568.8991, 7.633 min), 9 (red, m/z 618.8959, 8.048 min), and 10 (green, m/z 668.8927, 8.441 min)).

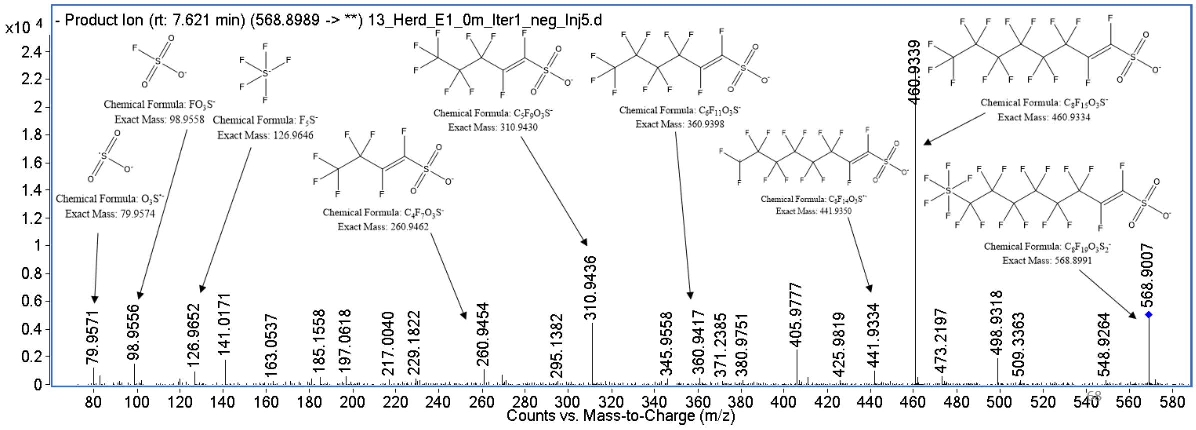


**Fig. S25b:** MS$^2$ spectrum (ESI$^-$, 32.1 eV, soil *S1*, 0 – 0.5 m, first extract, iterative MS$^2$) of $SF_5$-U-PFOS (m/z 568.8991, 7.621 min).

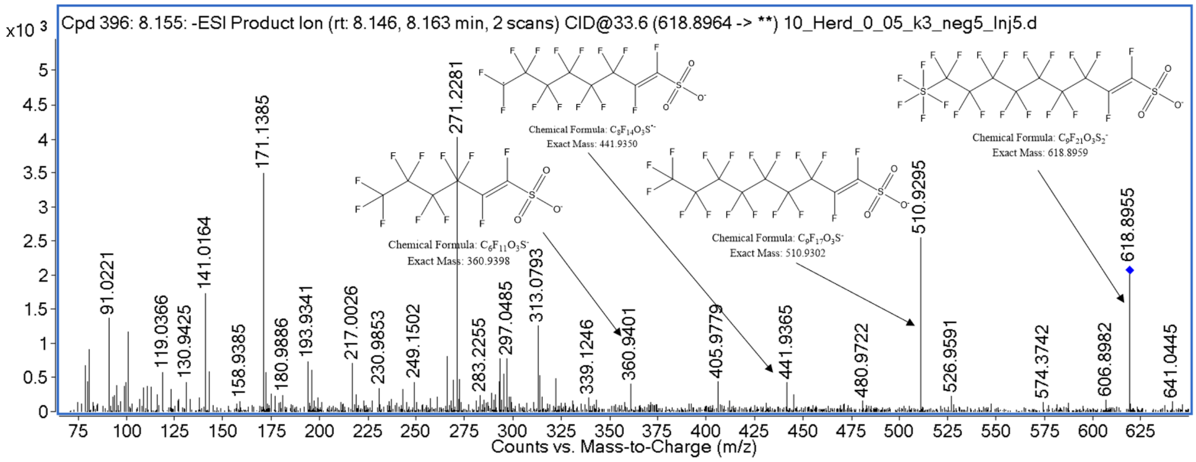


**Fig. S25c:** MS$^2$ spectrum (ESI$^-$, 33.6 eV, soil *S1*, 0 – 0.5 m, combined extract, iterative MS$^2$) of $SF_5$-U-PFNS (m/z 618.8959, 8.146 and 8.163 min).

# 3. PFASAm and PFASAm-Pr derivatives

### PFASAms (n = 3-8)

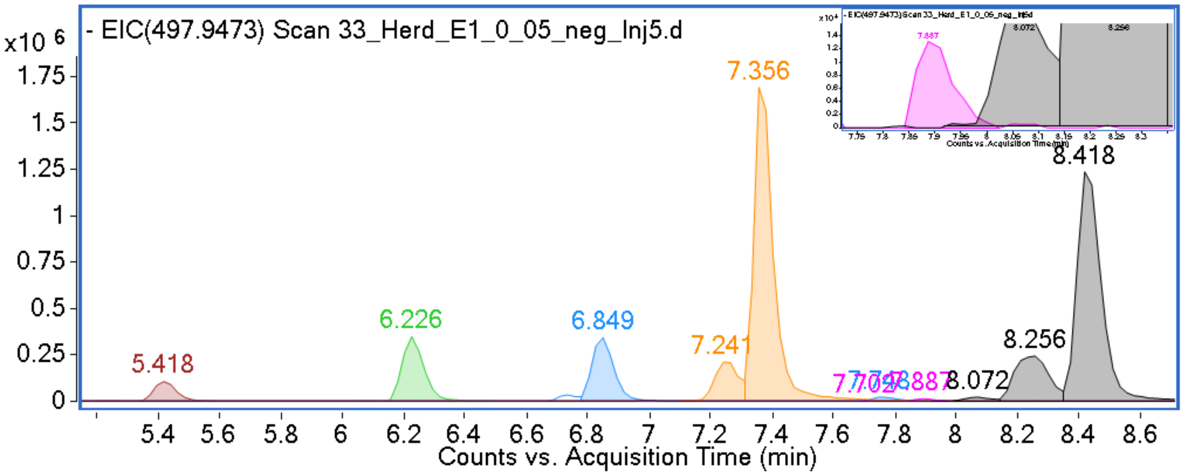


**Fig. S26a:** Chromatogram (ESI-, soil *S1*, 0 – 0.5 m, first extract) of PFASAms (n = 3 (red, m/z 247.9622, 5.418 min), 4 (green, m/z 297.9590, 6.226 min), 5 (blue, m/z 347.9558, 6.849 min), 6 (orange, m/z 397.9526, 7.241 and 7.356 min), 7 (pink, m/z 447.9494, 7.887 min), and 8 (grey, m/z 497.9462, 8.072, 8.256, and 8.418 min)). Scale-up in top right corner.

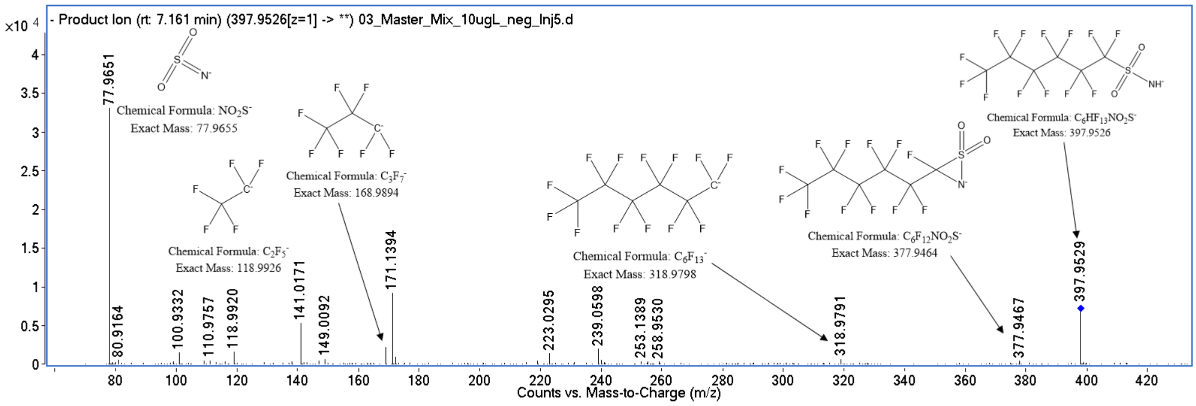


**Fig. S26b:** $MS^2$ spectrum (ESI-, 26.9 eV, reference standard) of PFHxSAm (m/z 397.9526, 7.161 min).

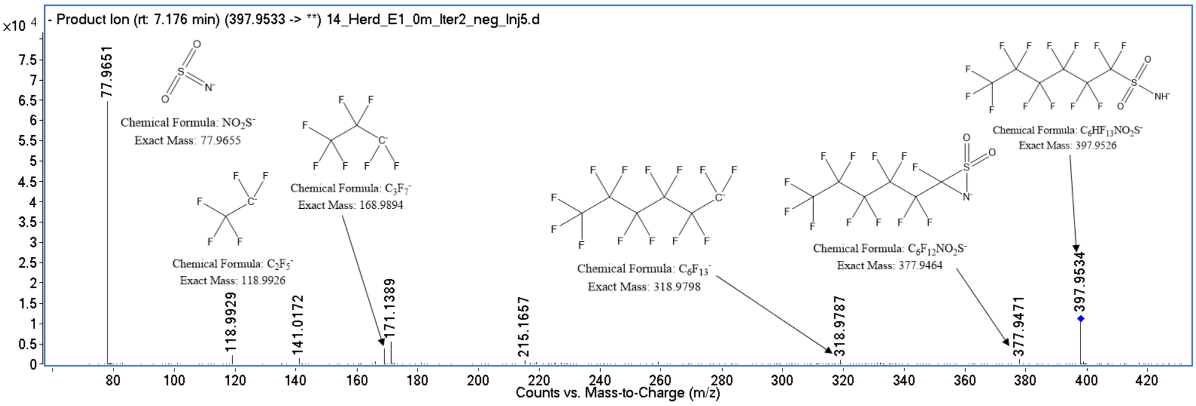


**Fig. S26c:** $MS^2$ spectrum (ESI-, 26.9 eV, soil *S1*, 0 – 0.5 m, first extract, iterative $MS^2$) of PFHxSAm (m/z 397.9526, 7.176 min).

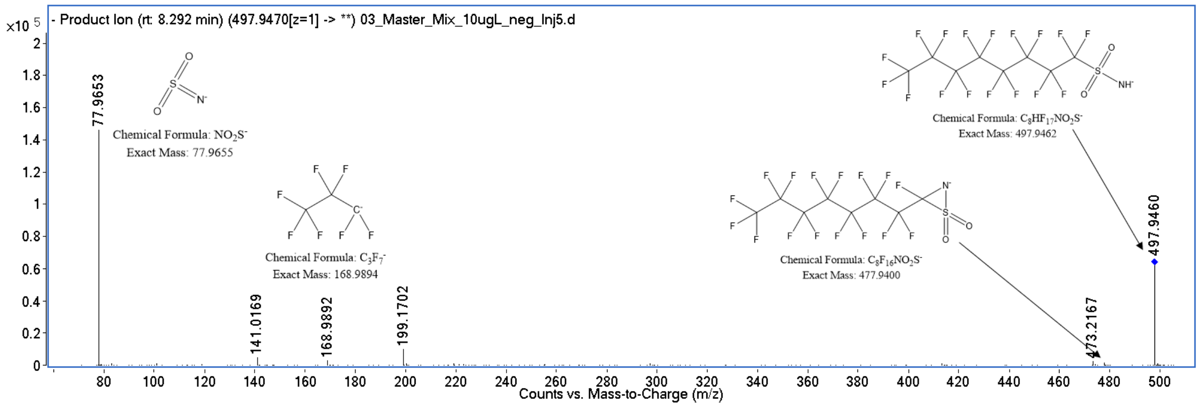


**Fig. S26d:** MS[2] spectrum (ESI-, 29.9 eV, reference standard) of PFOSAm (m/z 497.9462, 8.292 min).

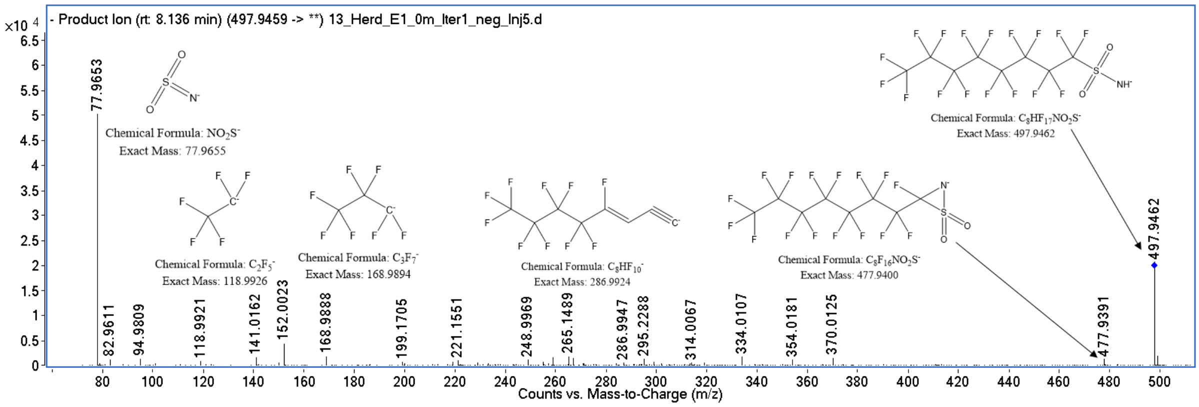


**Fig. S26e:** MS[2] spectrum (ESI-, 29.9 eV, soil *S1*, 0 – 0.5 m, first extract, iterative MS[2]) of PFOSAm (m/z 497.9462, 8.136 min).

## PFASAm-Pr-DiMeAm (n = 6, 8)

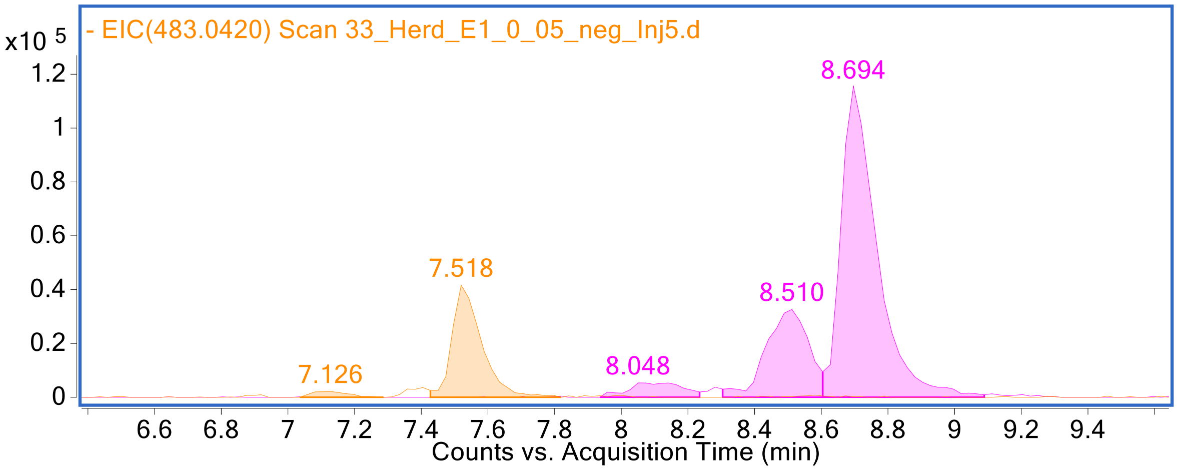


**Fig. S27a:** Chromatogram (ESI-, soil *s*, 0 – 0.5 m, first extract) of PFHxSAm-Pr-DiMeAm (orange peak, m/z 483.0417, 7.126 and 7.518 min) and PFOSAm-Pr-DiMeAm (pink peak, m/z 583.0353, 8.510 and 8.694 min).

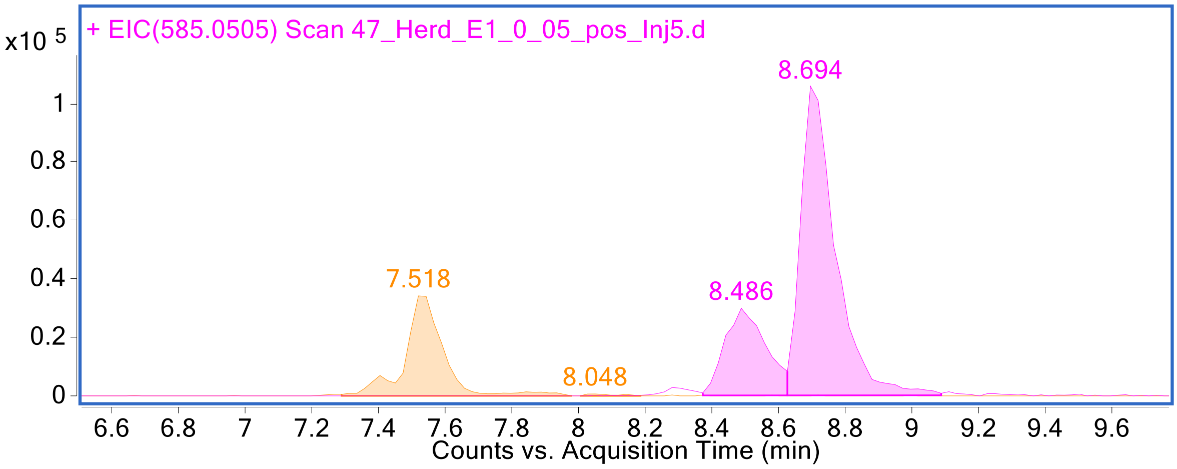


**Fig. S27b:** Chromatogram (ESI$^+$, soil *S1*, 0 – 0.5 m, first extract) of PFHxSAm-Pr-DiMeAm (orange peak, m/z 485.0563, 7.518 min) and PFOSAm-Pr-DiMeAm (pink peak, m/z 585.0499, 8.486 and 8.694 min).

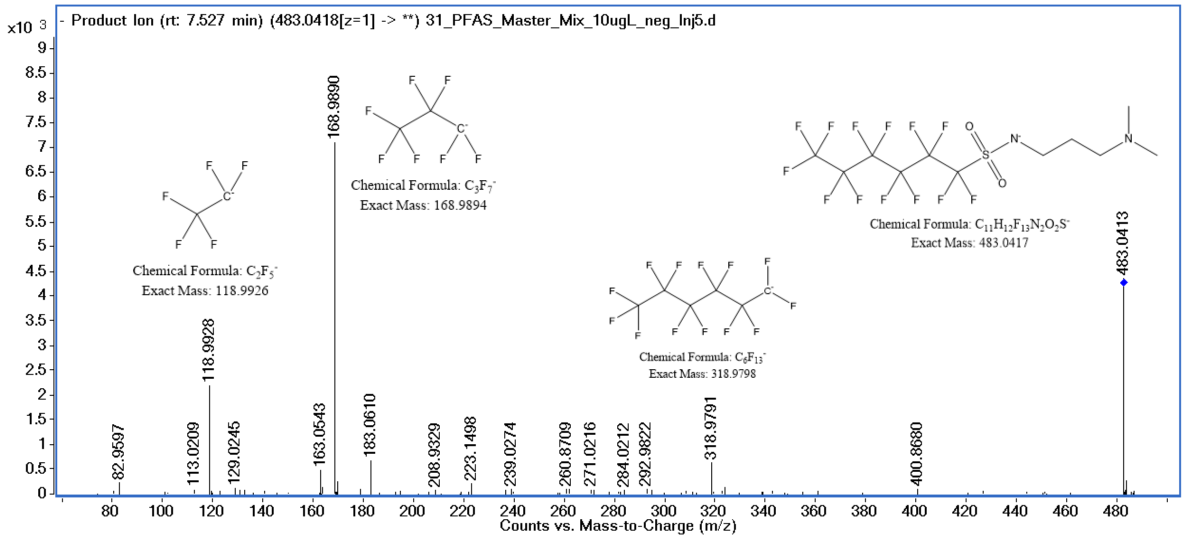


**Fig. S27c:** MS$^2$ spectrum (ESI$^-$, 29.5 eV, reference standard) of PFHxSAm-Pr-DiMeAm (m/z 483.0417, 7.527 min).

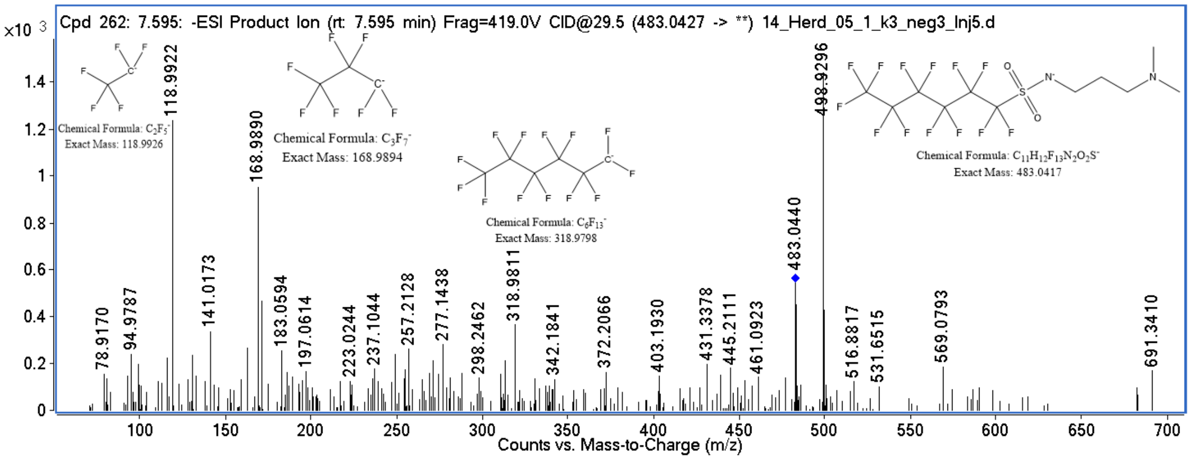


**Fig. S27d:** MS$^2$ spectrum (ESI$^-$, 29.5 eV, soil *S1*, 0.5 – 1 m, combined extract, iterative MS$^2$) of PFHxSAm-Pr-DiMeAm (m/z 483.0417, 7.595 min).

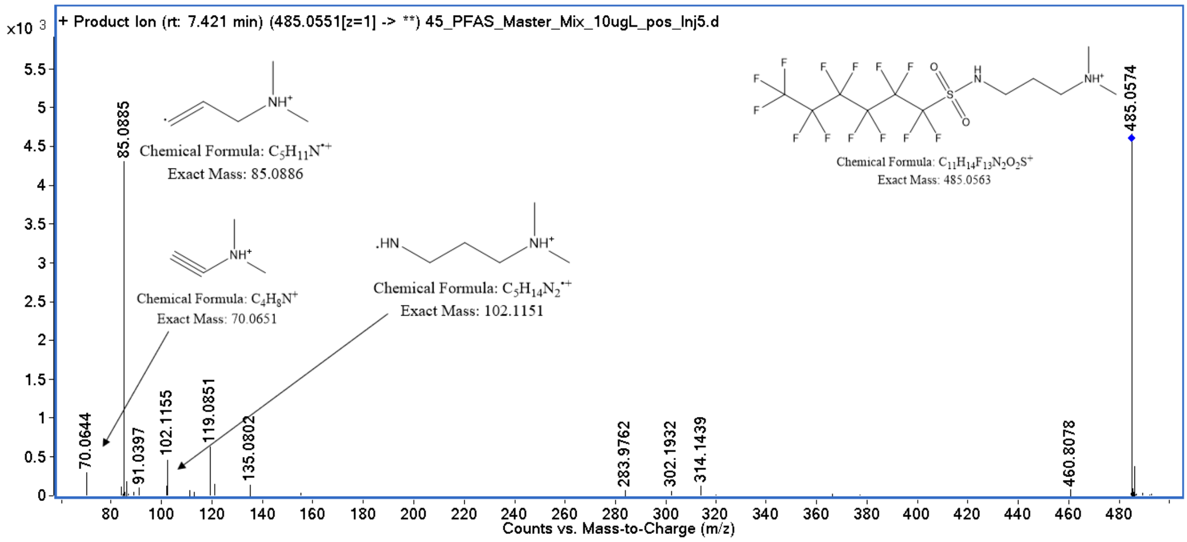

**Fig. S27e:** $MS^2$ spectrum ($ESI^+$, 29.6 eV, reference standard) of PFHxSAm-Pr-DiMeAm (m/z 485.0563, 7.421 min).

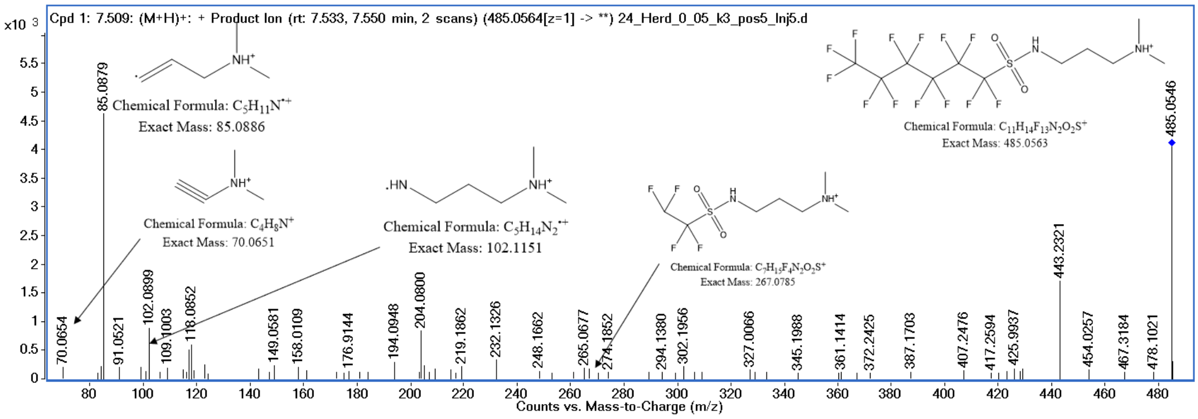

**Fig. S27f:** $MS^2$ spectrum ($ESI^+$, 29.6 eV, soil *S1*, 0 – 0.5 m, combined extract, iterative $MS^2$) of PFHxSAm-Pr-DiMeAm (m/z 485.0563, 7.533 and 7.550 min).

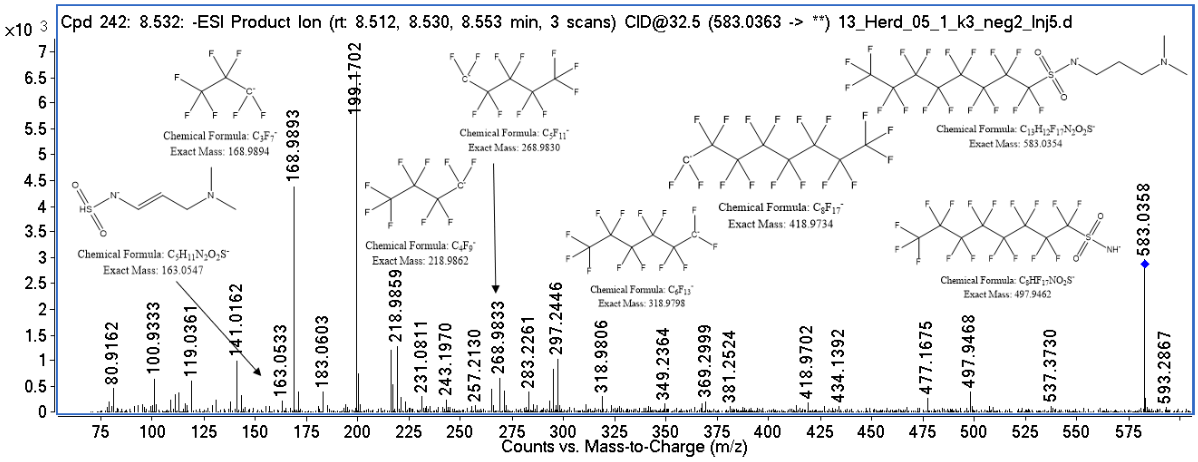

**Fig. S27g:** $MS^2$ spectrum ($ESI^-$, 32.5 eV, soil *S1*, 0.5 – 1 m, combined extract, iterative $MS^2$) of PFOSAm-Pr-DiMeAm (m/z 583.0353, 8.512, 8.530 and 8.553 min).

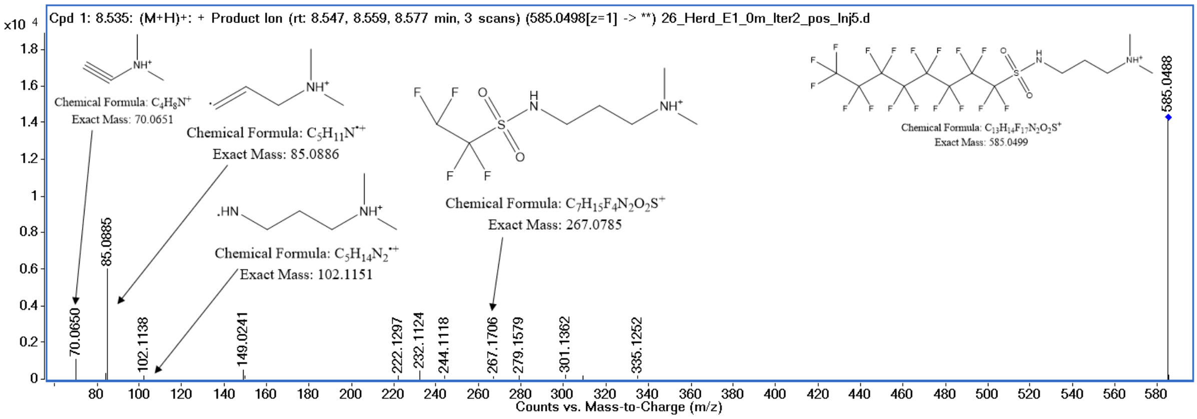


**Fig. S27h:** MS$^2$ spectrum (ESI$^+$, 32.6 eV, soil *S1*, 0 – 0.5 m, first extract, iterative MS$^2$) of PFOSAm-Pr-DiMeAm (m/z 585.0499, 8.547, 8.559 and 8.577 min).

## PFASAm-EtAs (n = 6, 8)

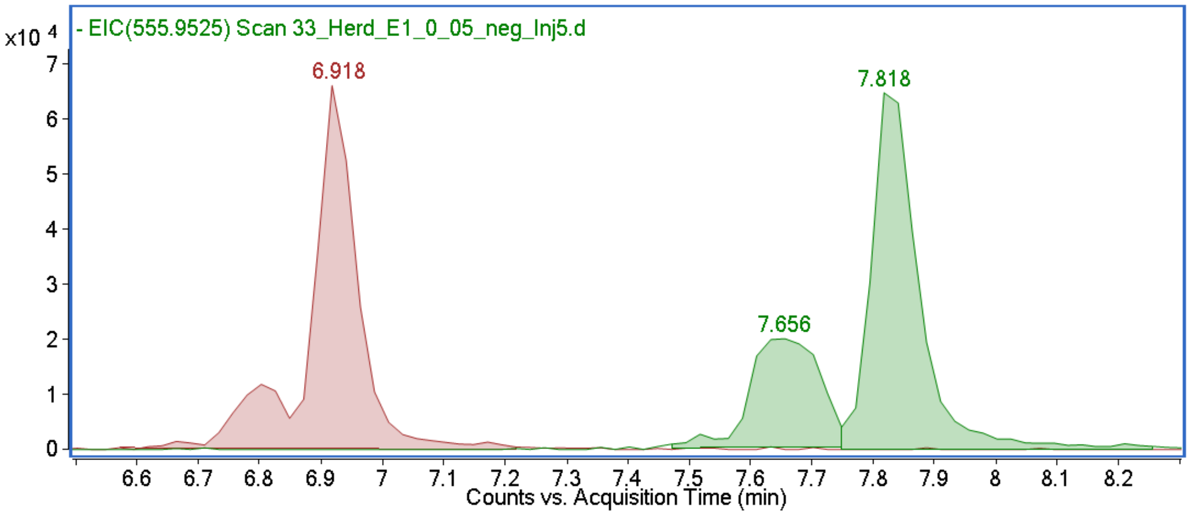


**Fig. S28a:** Chromatogram (ESI$^-$, soil *S1*, 0 – 0.5 m, first extract) of PFASA-EtA (n = 6 (red, m/z 455.9581, 6.918 min) and 8 (green, m/z 555.9517, 7.656 and 7.818 min)).

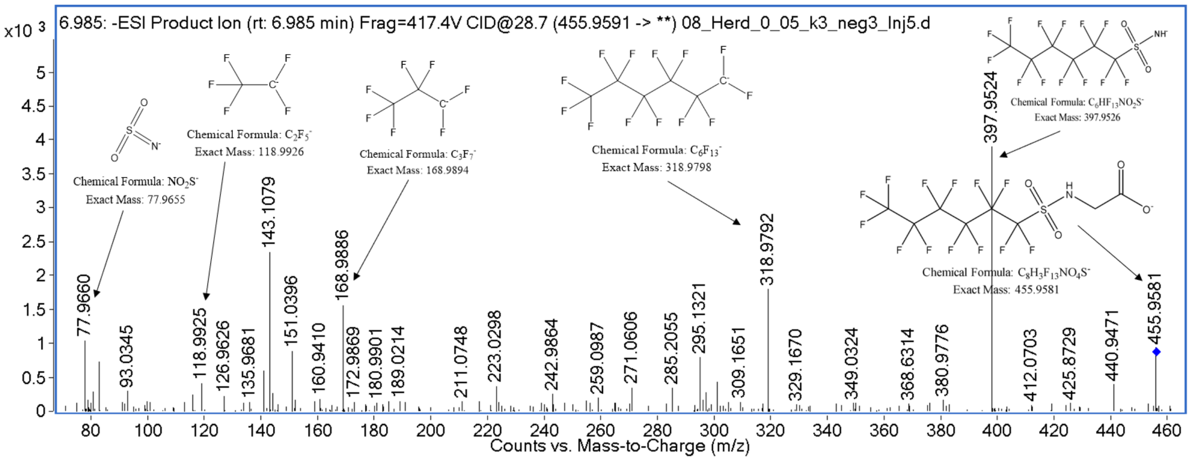


**Fig. S28b:** MS$^2$ spectrum (ESI$^-$, 28.7 eV, soil *S1*, 0 – 0.5 m, combined extract, iterative MS$^2$) of PFHxSAm-EtA (m/z 455.9581, 6.985 min).

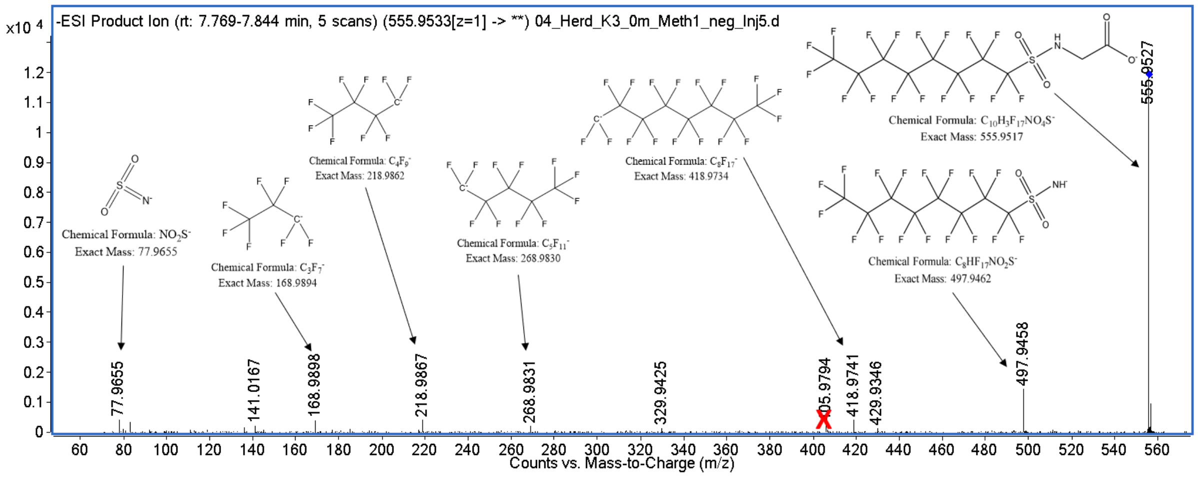


**Fig. S28c:** $MS^2$ spectrum (ESI$^-$, different CEs (10 – 40 eV), soil *S1*, 0 – 0.5 m, combined extract, targeted $MS^2$) of PFOSAm-EtA (m/z 555.9517, 7.769 – 7.844 min).

## PFASAm-PrSAs (n = 4, 5, 6)

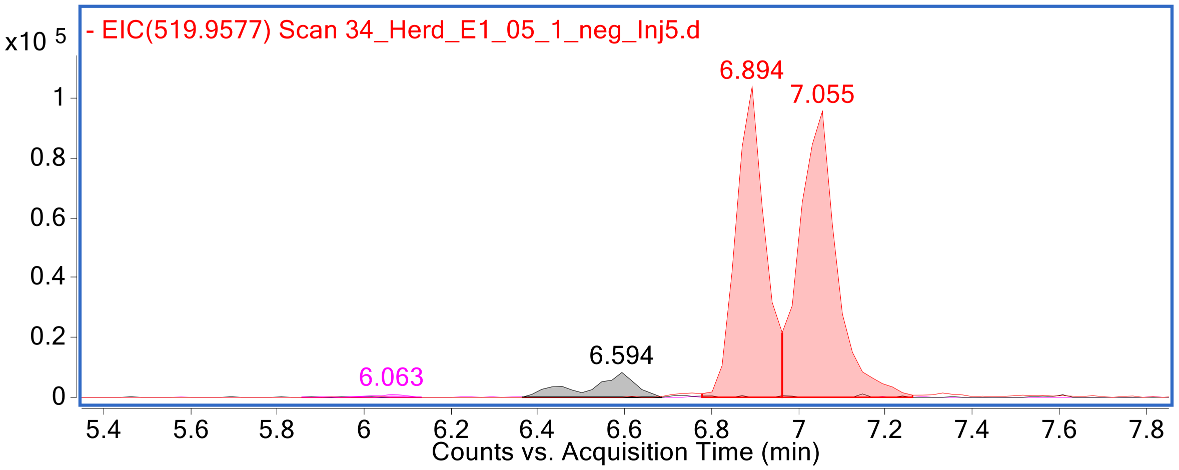


**Fig. S29a:** Chromatogram (ESI$^-$, soil *S1*, 0.5 – 1 m, first extract) of PFASAm-PrSAs (n = 4 (pink, m/z 419.9627, 6.063 min), 5 (grey, m/z 469.9595, 6.594 min), and 6 (red, m/z 519.9564, 6.894 and 7.055 min)).

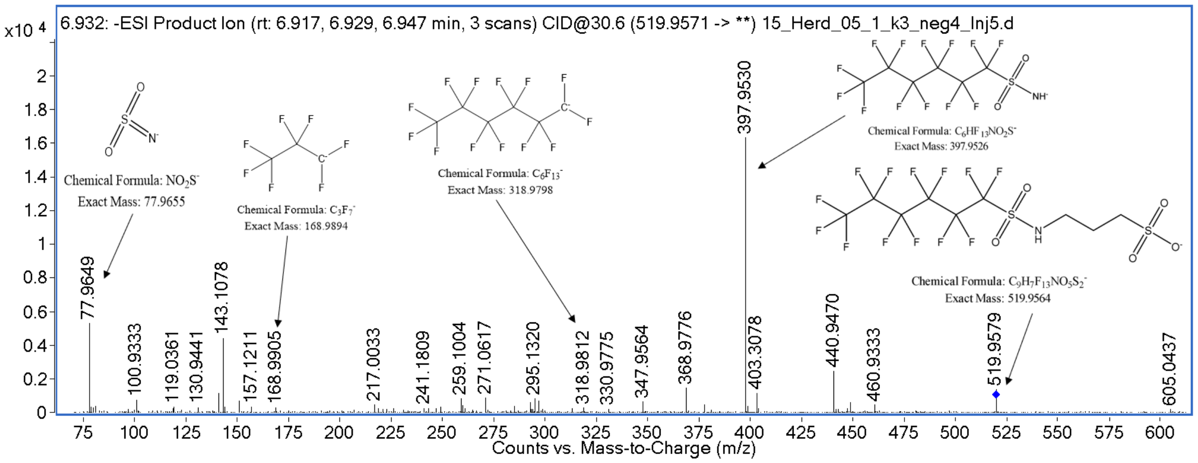


**Fig. S29b:** $MS^2$ spectrum (ESI$^-$, 30.6 eV, soil *S1*, 0.5 – 1 m, combined extract, iterative $MS^2$) of PFHxSAm-PrSA (m/z 519.9564, 6.917, 6.929, and 6.947 min).

## PFASAm-Pr-Bs (n = 6, 8)

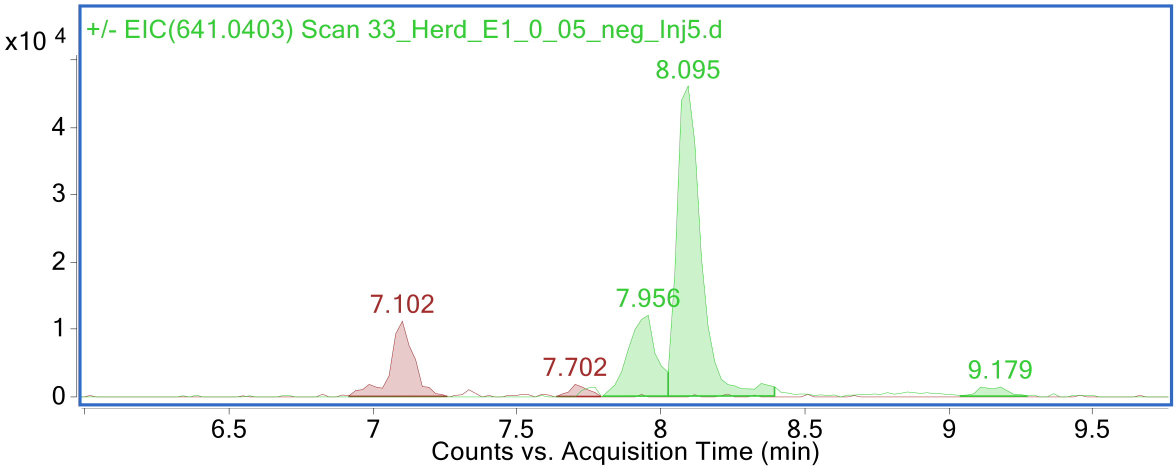


**Fig. S30a:** Chromatogram (ESI$^-$, soil *S1*, 0 – 0.5 m, first extract) of PFASAm-Pr-Bs (n = 6 (red, m/z 541.0472, 7.102 min) and 8 (green, m/z 641.0408, 7.956 and 8.095 min)).

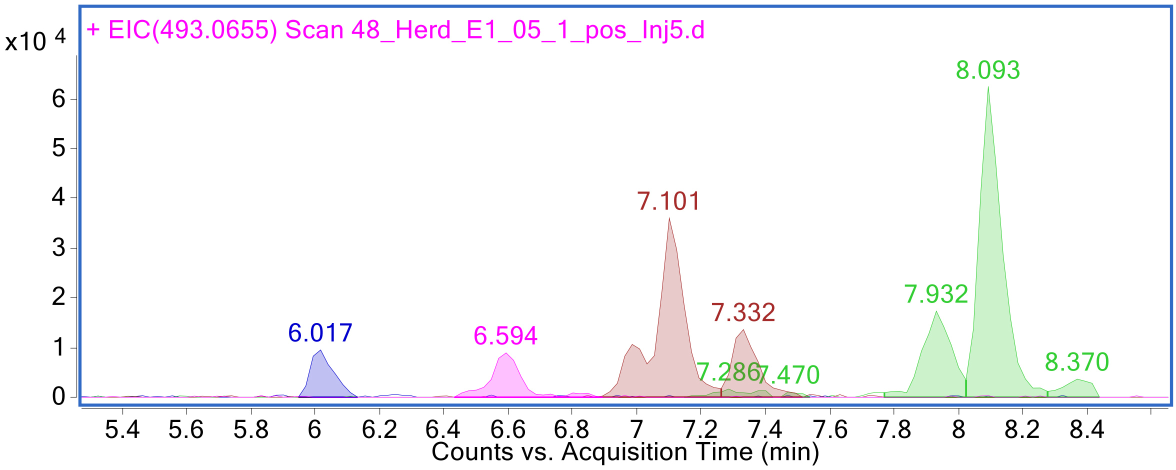


**Fig. S30b:** Chromatogram (ESI$^+$, soil *S1*, 0.5 – 1 m, first extract) of PFASAm-Pr-Bs (n = 4 (blue, m/z 443.0682, 6.017 min), 5 (pink, m/z 493.0650, 6.594 min), 6 (red, m/z 543.0618, 7.101 and 7.332 min), and 8 (green, m/z 643.0554, 7.932, 8.093, and 8.370 min)).

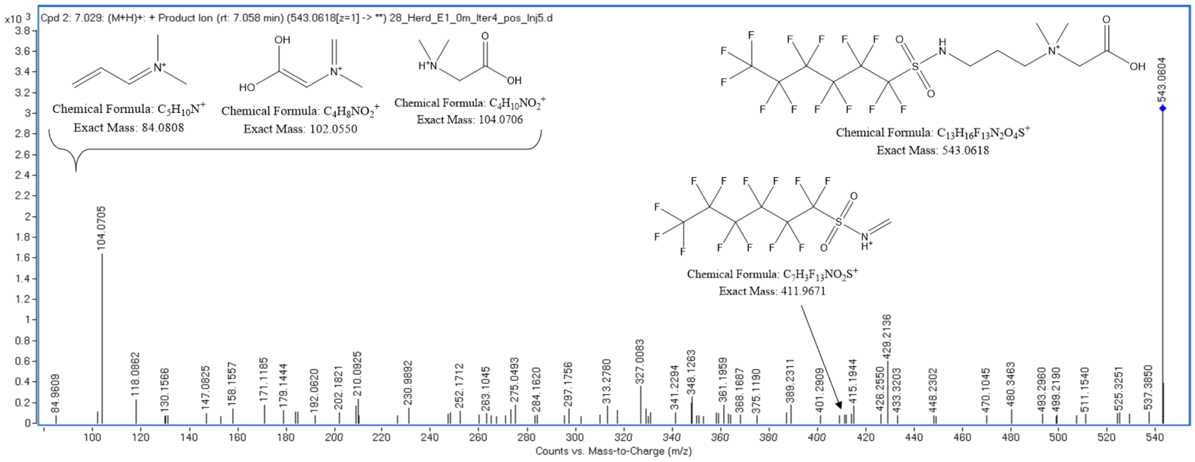


**Fig. S30c:** MS$^2$ spectrum (ESI$^+$, 31.3 eV, soil *S1*, 0 – 0.5 m, first extract, iterative MS$^2$) of PFHxSAm-Pr-B (m/z 543.0618, 7.058 min).

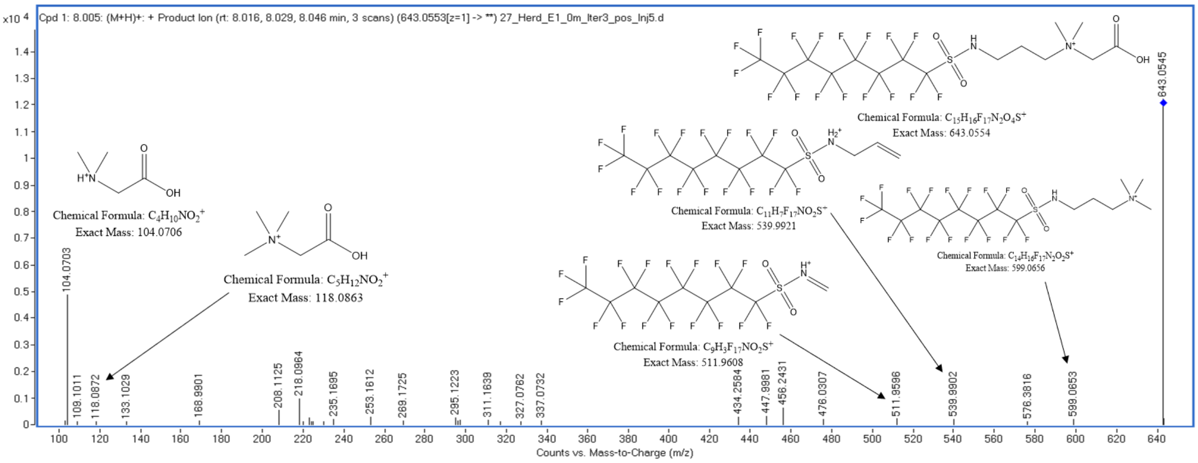


**Fig. S30d:** $MS^2$ spectrum ($ESI^+$, 34.3 eV, soil *S1*, 0 – 0.5 m, first extract, iterative $MS^2$) of PFOSAm-Pr-B (m/z 643.0554, 8.016, 8.029, and 8.046 min).

## PFASAm-Pr-TriMeAms (n = 6, 8)

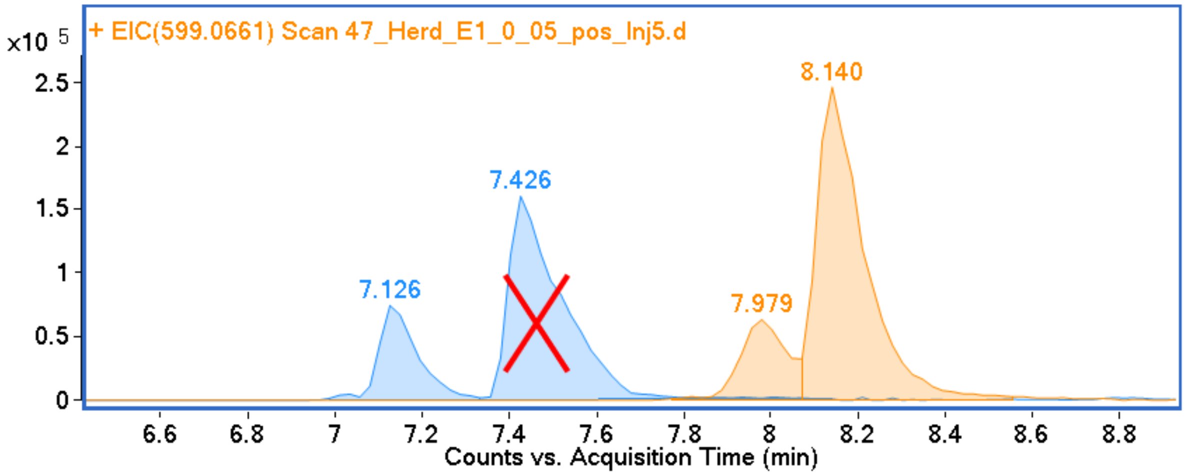


**Fig. S31a:** Chromatogram ($ESI^+$, soil *S1*, 0 – 0.5 m, first extract) of PFASAm-Pr-TriMeAms (n = 6 (blue, m/z 499.0719, 7.126 min) and 8 (orange, m/z 599.0656, 7.979 and 8.140 min)). Note: The second peak of PFHxSAm-Pr-TriMeAm (blue) is crossed out as it belongs to 6:2 FTSAm-Pr-MeAm.

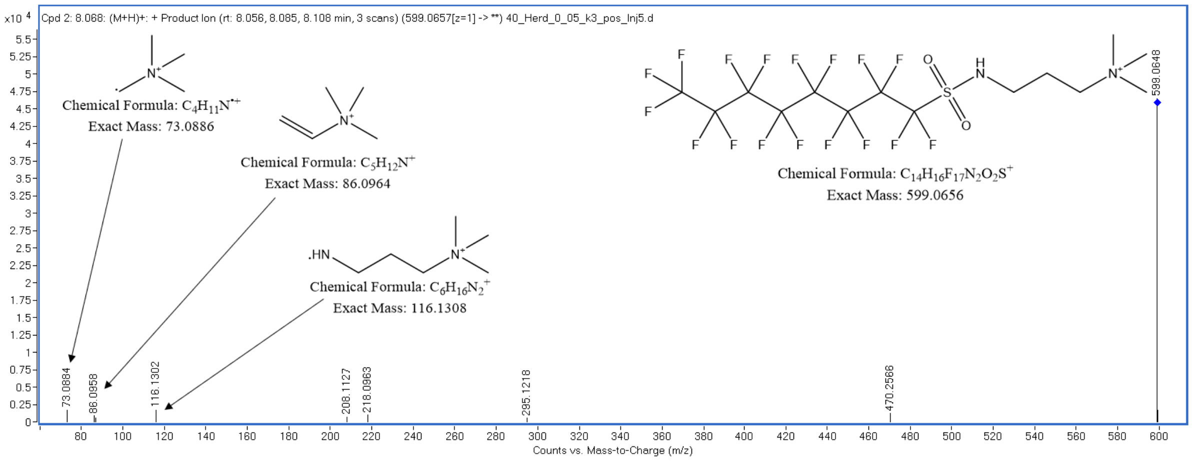


**Fig. S31b:** $MS^2$ spectrum ($ESI^+$, 33.0 eV, soil *S1*, 0 – 0.5 m, combined extract) of PFOSAm-Pr-TriMeAm (m/z 599.0656, 8.056, 8.085, and 8.108 min).

## PFASAm-Mes (n = 4, 6, 8)

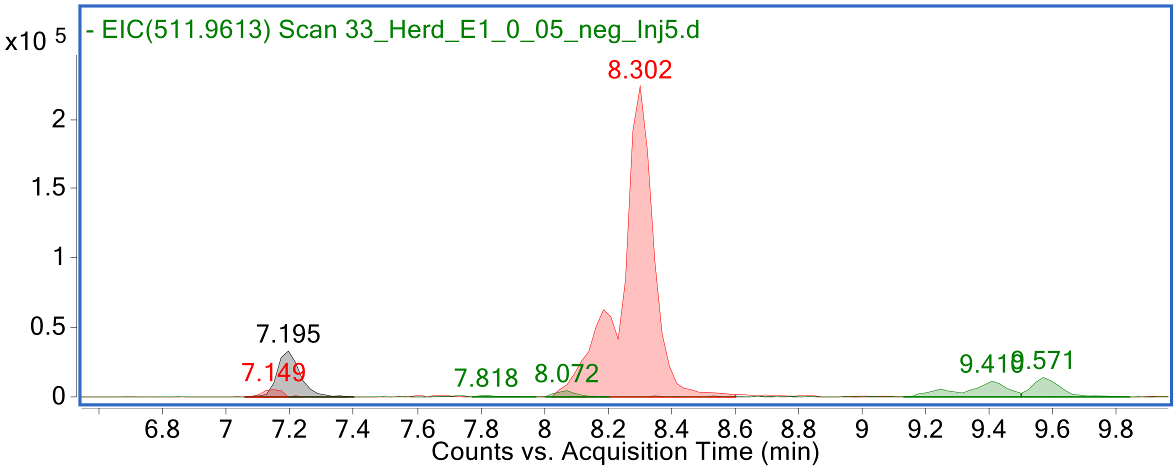


**Fig. S32a:** Chromatogram (ESI$^-$, soil *S1*, 0 – 0.5 m, first extract) of PFASAm-Mes (n = 4 (grey, m/z 311.9746, 7.195 min), 6 (red, m/z 411.9682, 8.302 min), and 8 (green, m/z 511.9619, 9.410 min)).

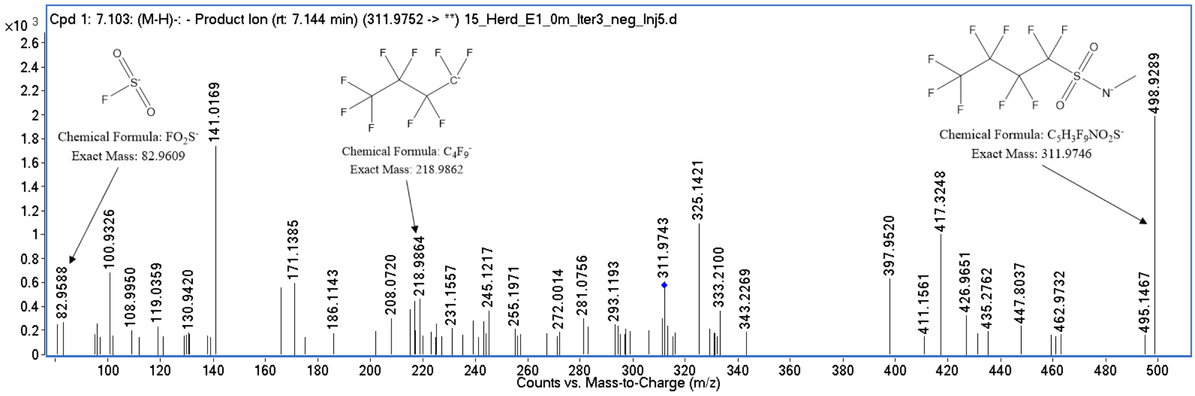


**Fig. S32b:** MS$^2$ spectrum (ESI$^-$, 24.4 eV, soil *S1*, 0 – 0.5 m, first extract, iterative MS$^2$) of PFBSAm-Me (m/z 311.9746, 7.144 min).

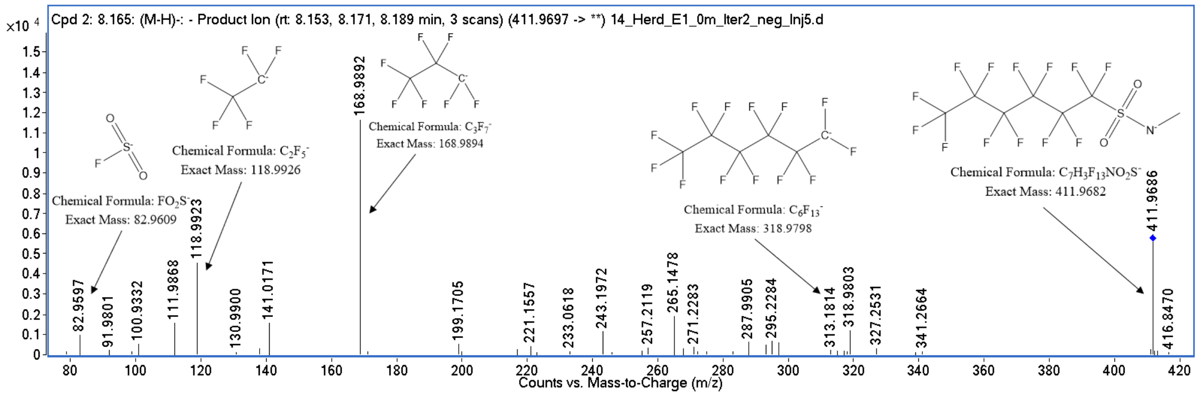


**Fig. S32c:** MS$^2$ spectrum (ESI$^-$, 27.4 eV, soil *S1*, 0 – 0.5 m, first extract, iterative MS$^2$) of PFHxSAm-Me (m/z 411.9682, 8.153, 8.171, and 8.189 min).

**PFASAm-*N*-PrSA-*N*-Pr-DiMeAms (n = 4, 5, 6)**

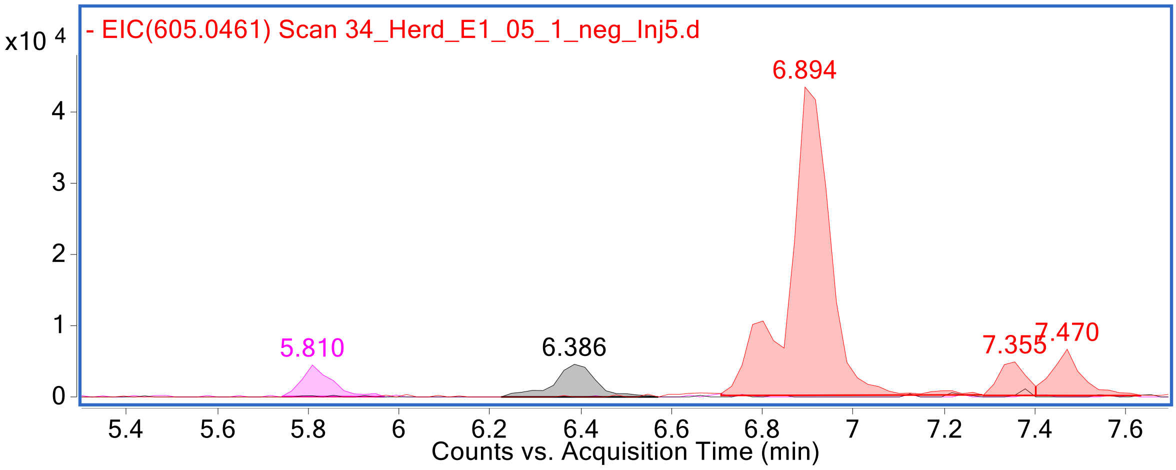


**Fig. S33a:** Chromatogram (ESI$^-$, soil *S1*, 0.5 – 1 m, first extract) of PFASAm-*N*-PrSA- -*N*-Pr-DiMeAms (n = 4 (pink, m/z 505.0519, 5.810 min), 5 (grey, m/z 555.0487, 6.386 min), and 6 (red, m/z 605.0455, 6.894 min)).

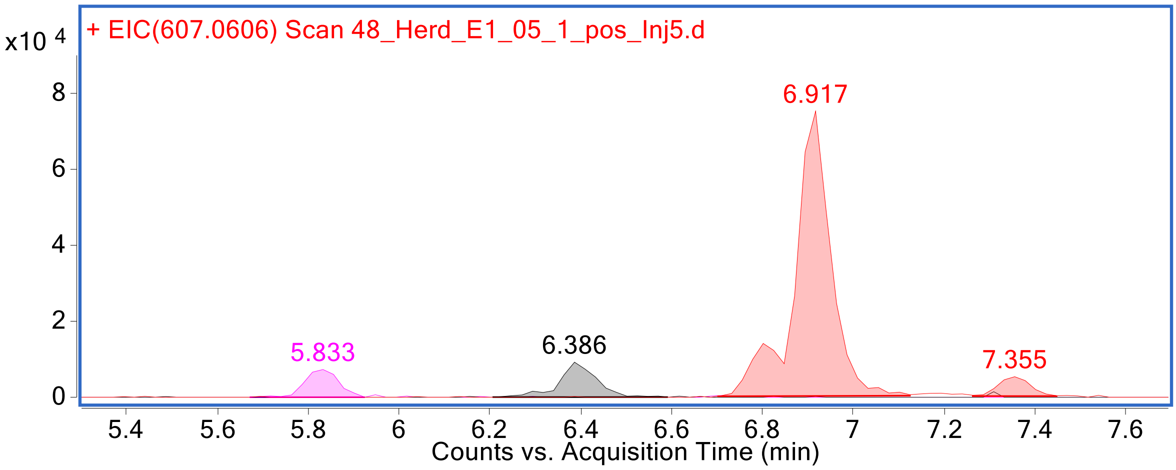


**Fig. S33b:** Chromatogram (ESI$^+$, soil *S1*, 0.5 – 1 m, first extract) of PFASAm-*N*-PrSA--*N*-Pr-DiMeAms (n = 4 (pink, m/z 557.0633, 5.833 min), 5 (grey, m/z 557.0633, 6.386 min), and 6 (red, m/z 607.0601, 6.917 min)).

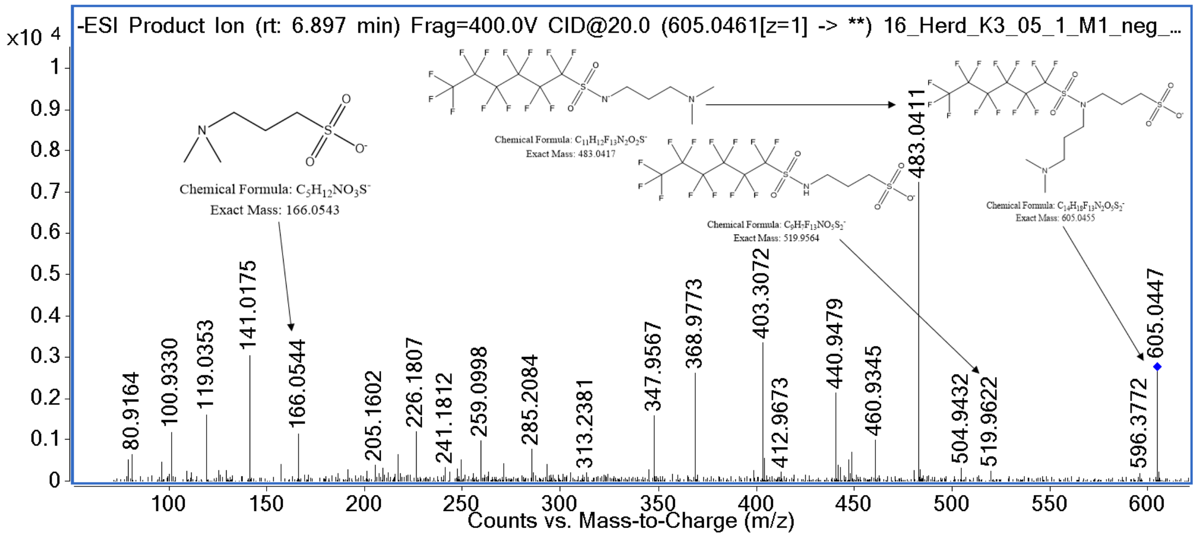


**Fig. S33c:** MS$^2$ spectrum (ESI$^-$, 20.0 eV, soil *S1*, 0.5 – 1 m, combined extract, targeted MS$^2$) of PFHxSAm-*N*-PrSA--*N*-Pr-DiMeAm (m/z 605.0455, 6.897 min).

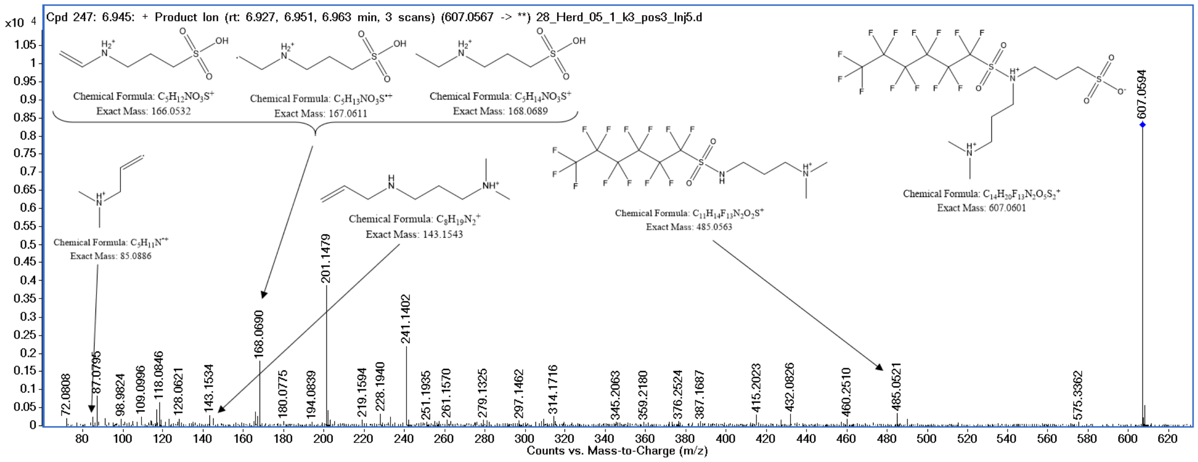


**Fig. S33d:** $MS^2$ spectrum ($ESI^+$, 33.2 eV, soil *S1*, 0.5 – 1 m, combined extract, iterative $MS^2$) of PFHxSAm-*N*-PrSA--*N*-Pr-DiMeAm (m/z 607.0601, 6.927, 6.951, and 6.963 min).

# 4. FTBs

### n:1:2 FTBs (n = 5, 7, 9, 11, 13, 15)

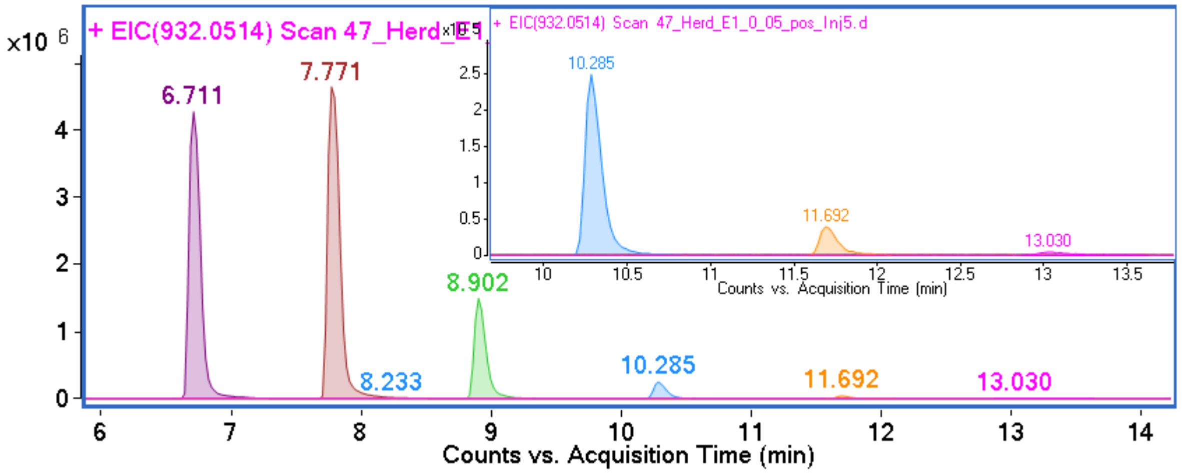


**Fig. S34a:** Chromatogram ($ESI^+$, soil *S1*, 0 – 0.5 m, first extract) of n:1:2 FTBs (n = 5 (violet, m/z 432.0827, 6.711 min), 7 (red, m/z 532.0764, 7.771 min), 9 (green, m/z 632.0700, 8.902 min), 11 (blue, m/z 732.0636, 10.285 min), 13 (orange, m/z 832.0572, 11.692 min), and 15 (pink, m/z 932.0508, 13.030 min)). Scale-up in top right corner.

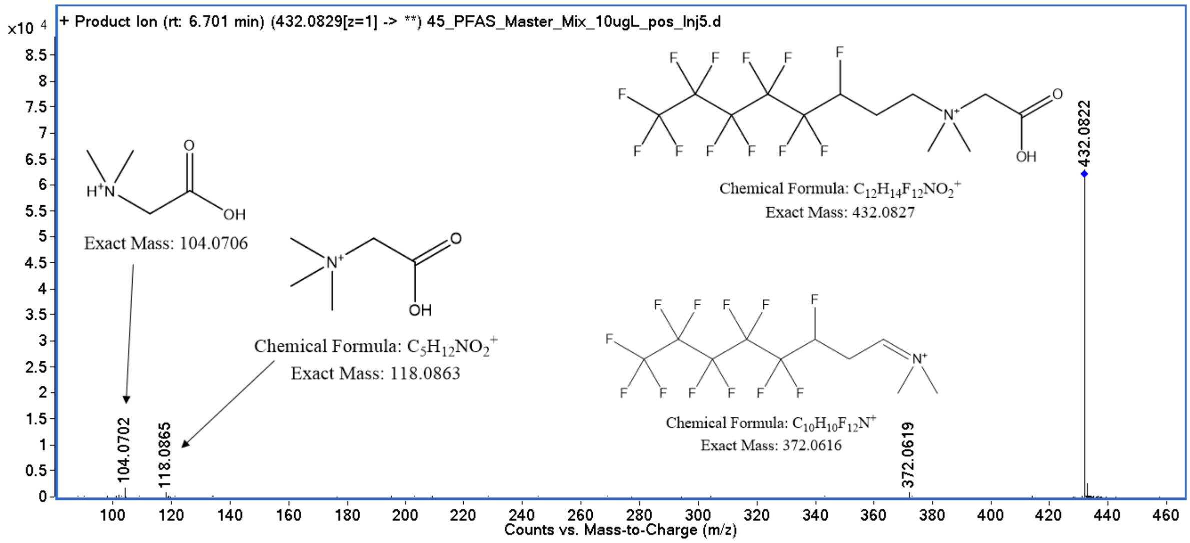


**Fig. S34b:** $MS^2$ spectrum ($ESI^+$, 28.0 eV, reference standard) of 5:1:2 FTB (m/z 432.0827, 6.701 min).

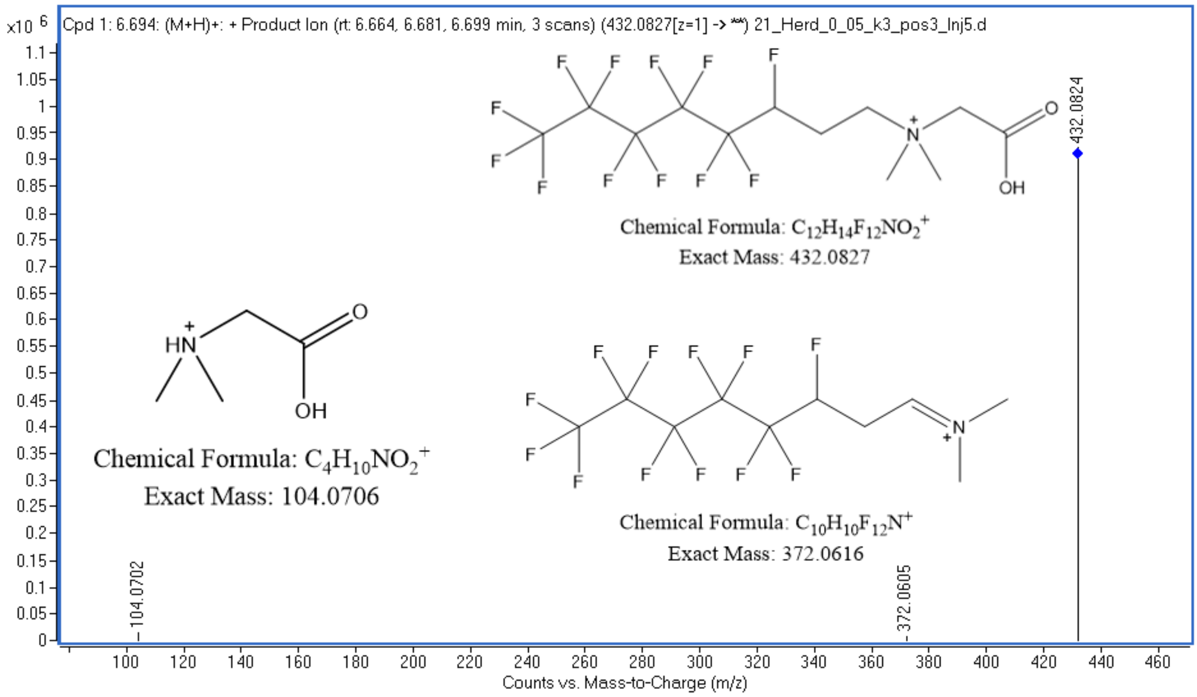


**Fig. S34c:** $MS^2$ spectrum ($ESI^+$, 28.0 eV, soil *S1*, 0 – 0.5 m, combined extract, iterative $MS^2$) of 5:1:2 FTB (m/z 432.0827, 6.694 min).

## n:3 FTBs (n = 5, 7, 9, 11, 13)

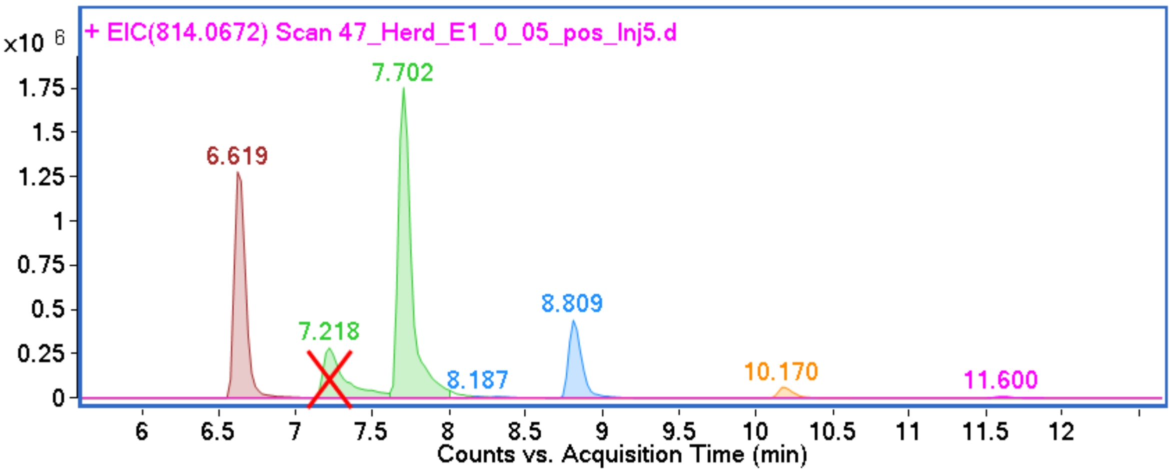


**Fig. S35a:** Chromatogram (ESI$^+$, soil *S1*, 0 – 0.5 m, first extract) of n:3 FTBs (n = 5 (red, m/z 414.0922, 6.619 min), 7 (green, m/z 514.0858, 7.702 min), 9 (blue, m/z 614.0794, 8.809 min), 11 (orange, m/z 714.0730, 10.170 min), and 13 (pink, m/z 814.0666, 11.600 min)).

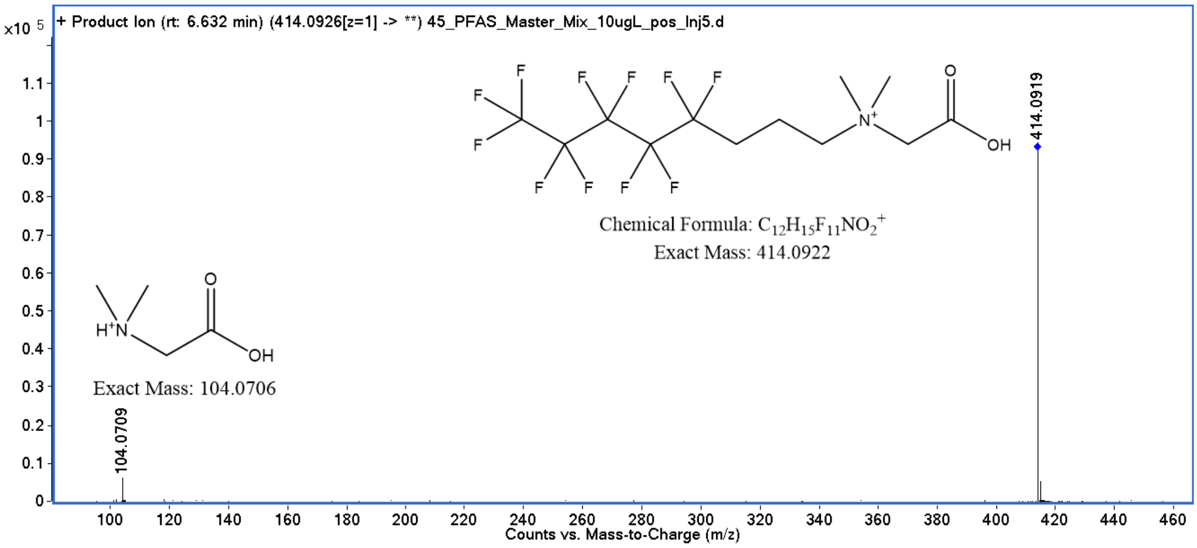


**Fig. S35b:** MS$^2$ spectrum (ESI$^+$, 27.4 eV, reference standard) of 5:3 FTB (m/z 414.0922, 6.632 min).

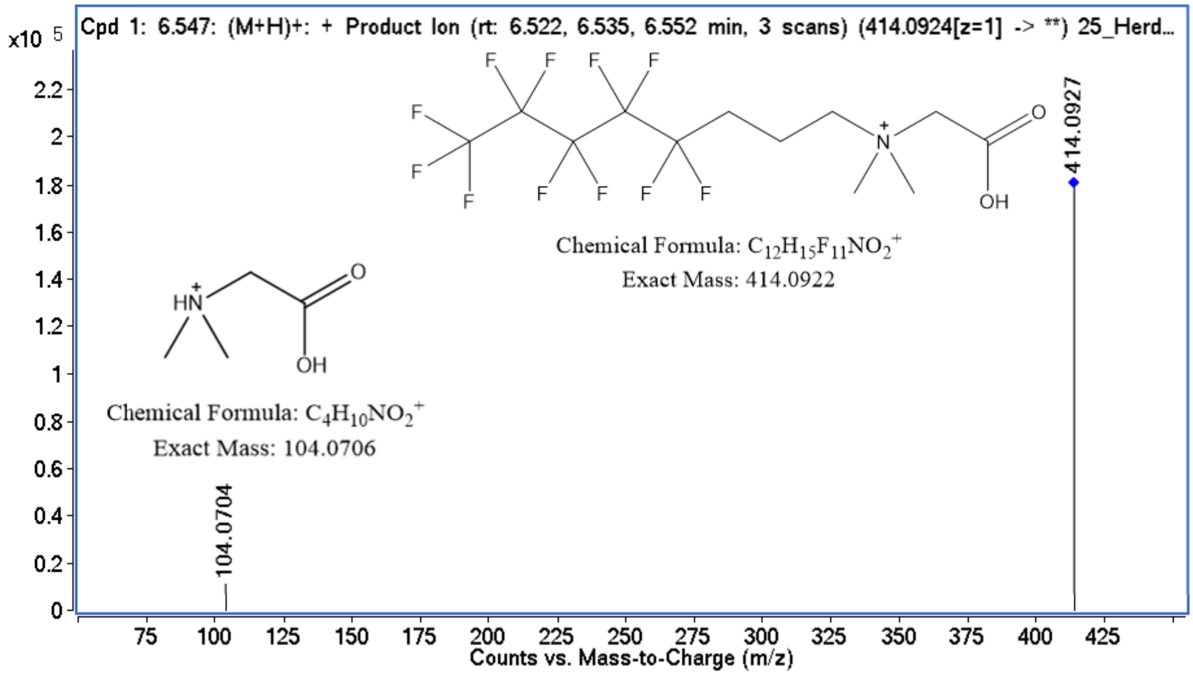


**Fig. S35c:** $MS^2$ spectrum ($ESI^+$, 27.4 eV, soil *S1*, 0 – 0.5 m, first extract, iterative $MS^2$) of 5:3 FTB (m/z 414.0922, 6.522, 6.535 and 6.552 min).

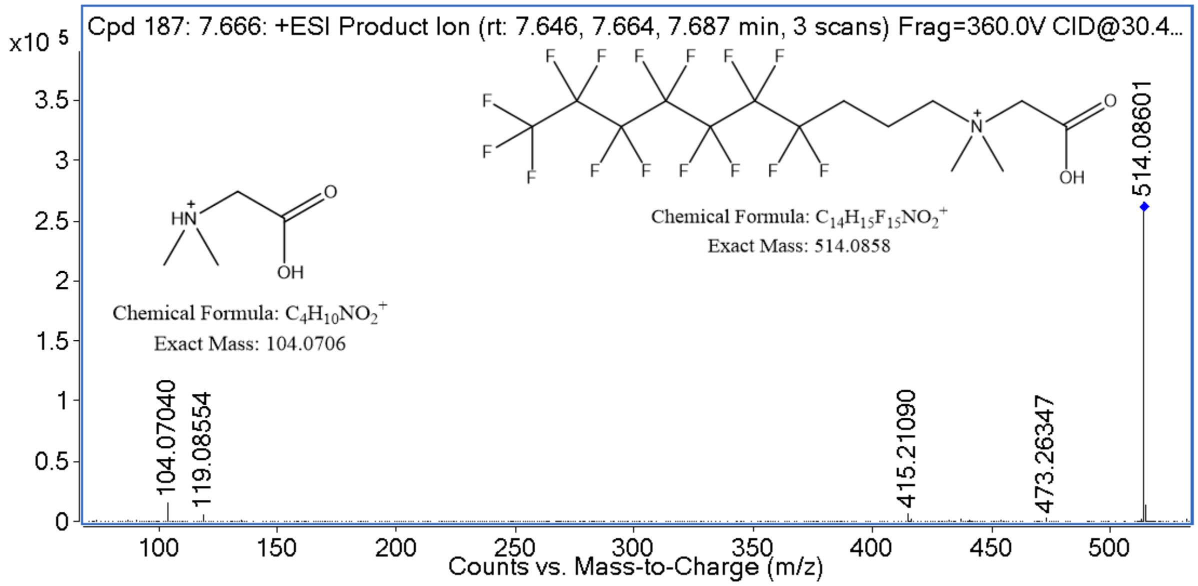


**Fig. S35d:** $MS^2$ spectrum ($ESI^+$, 30.4 eV, soil *S1*, 0 – 0.5 m, first extract, iterative $MS^2$) of 7:3 FTB (m/z 514.0858, 7.646, 7.664 and 7.687 min).

## 5. FTSAm-derivatives

### n:2 FTSAms (n = 6, 8, 10)

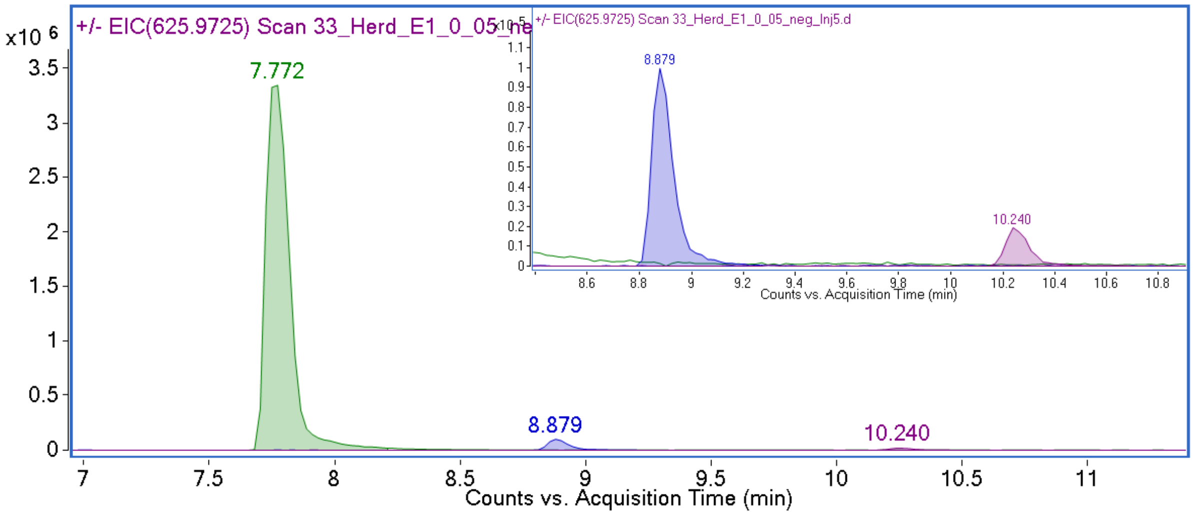


**Fig. S36a:** Chromatogram (ESI$^-$, soil *S1*, 0 – 0.5 m, first extract) of n:2 FTSAms (n = 6 (green, m/z 425.9839, 7.772 min), 8 (blue, m/z 525.9775, 8.879 min), and 10 (violet, m/z 625.9711, 10.240 min)). Scale-up in top right corner.

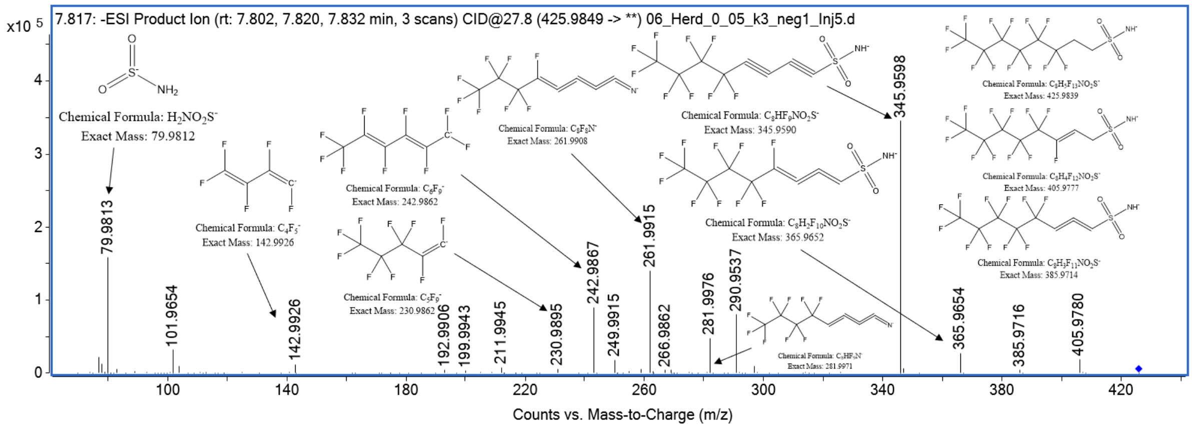


**Fig. S36b:** MS$^2$ spectrum (ESI$^-$, 27.8 eV, soil *S1*, 0 – 0.5 m, combined extract, iterative MS$^2$). of 6:2 FTSAm (m/z 425.9839, 7.802, 7.820, and 7.832 min).

## n:2 FTSAm-Pr-Bs (n = 6, 8, 10)

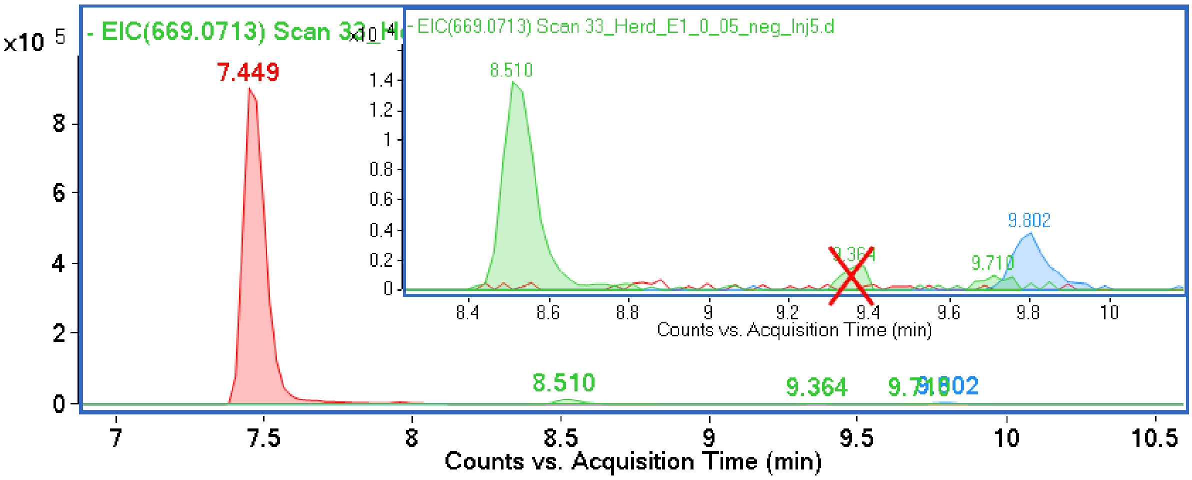


**Fig. S37a:** Chromatogram (ESI$^-$, soil *S1*, 0 – 0.5 m, first extract) of n:2 FTSAm-Pr-Bs (n = 6 (red, m/z 569.0785, 7.449 min), 8 (green, m/z 669.0721, 8.510 min), and 10 (blue, m/z 769.0657, 9.802 min)).

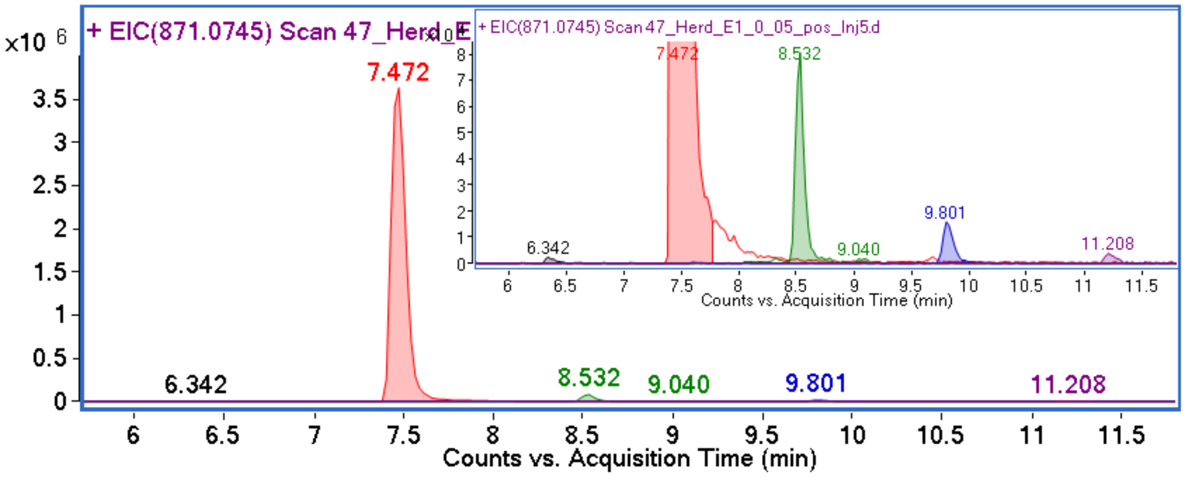


**Fig. S37b:** Chromatogram (ESI$^+$, soil *S1*, 0 – 0.5 m, first extract) of n:2 FTSAm-Pr-Bs (n = 4 (grey, m/z 471.0995, 6.342 min), 6 (red, m/z 571.0931, 7.472 min), 8 (green, m/z 671.0867, 8.532 min), 10 (blue, m/z 771.0803, 9.801 min), and 12 (violet, m/z 871.0739, 11.208 min)). Scale-up in top right corner.

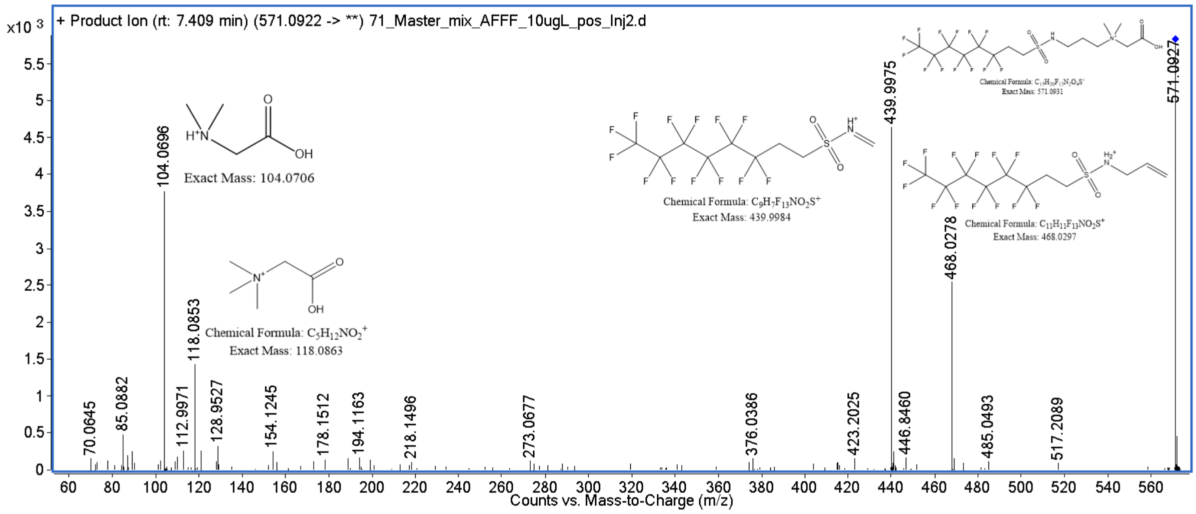


**Fig. S37c:** MS$^2$ spectrum (ESI$^+$, 32.1 eV, reference standard) of 6:2 FTSAm-Pr-B (m/z 571.0931, 7.409 min).

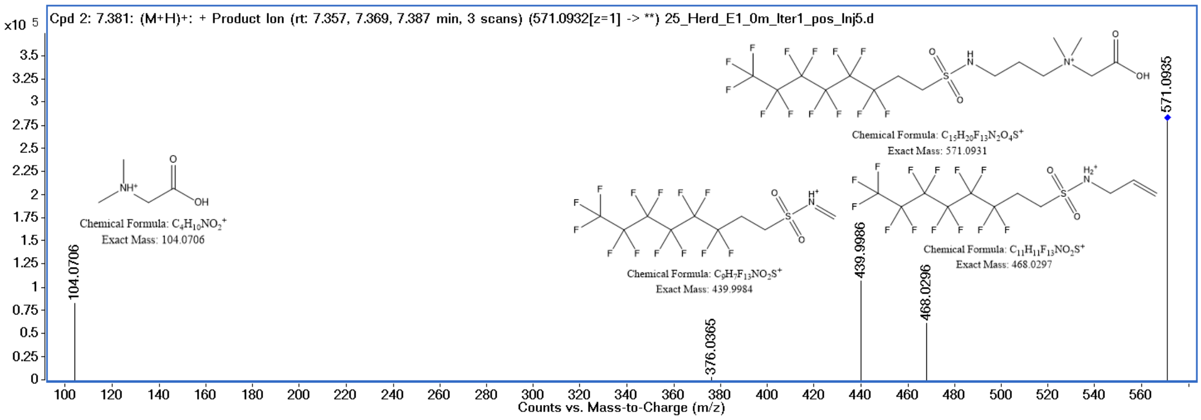


**Fig. S37d:** MS$^2$ spectrum (ESI$^+$, 32.1 eV, soil *S1*, 0 – 0.5 m, first extract, iterative MS$^2$) of 6:2 FTSAm-Pr-B (m/z 571.0931, 7.357, 7.369 and 7.387 min).

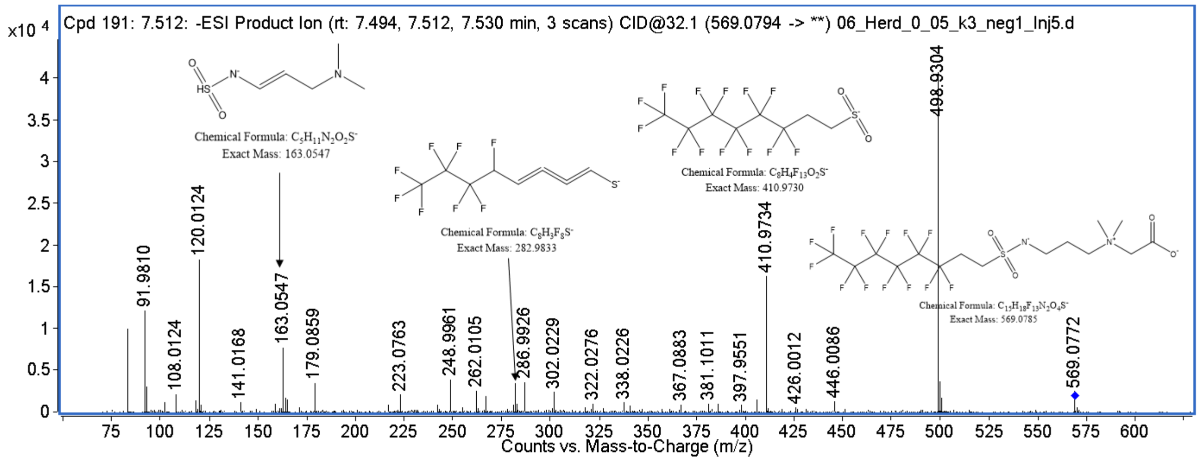


**Fig. S37e:** MS$^2$ spectrum (ESI$^-$, 32.1 eV, soil *S1*, 0 – 0.5 m, combined extract, iterative MS$^2$) of 6:2 FTSAm-Pr-B (m/z 569.0785, 7.494, 7.512 and 7.530 min).

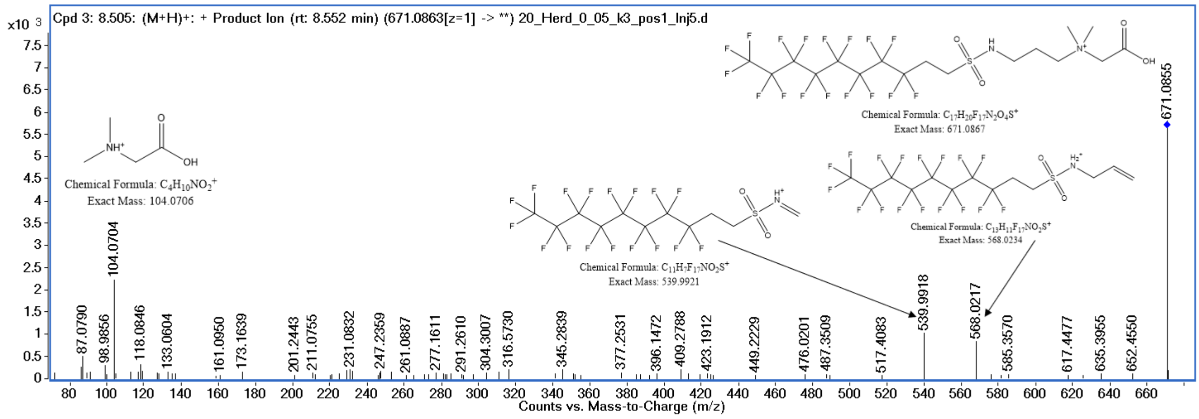


**Fig. S37f:** MS$^2$ spectrum (ESI$^+$, 35.1 eV, soil *S1*, 0 – 0.5 m, combined extract, iterative MS$^2$) of 8:2 FTSAm-Pr-B (m/z 671.0867, 8.552 min).

**n:2 FTSAm-Pr-DiMeNOs (n = 6, 8)**

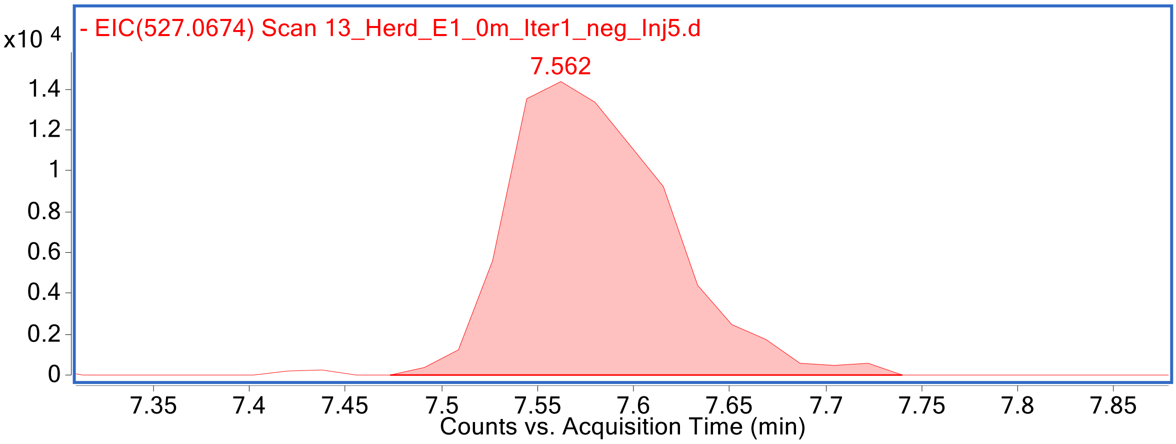


**Fig. S38a:** Chromatogram (ESI-, soil *S1*, 0 – 0.5 m, first extract) of 6:2 FTSAm-Pr-DiMeNO (red, m/z 527.0680, 7.562 min).

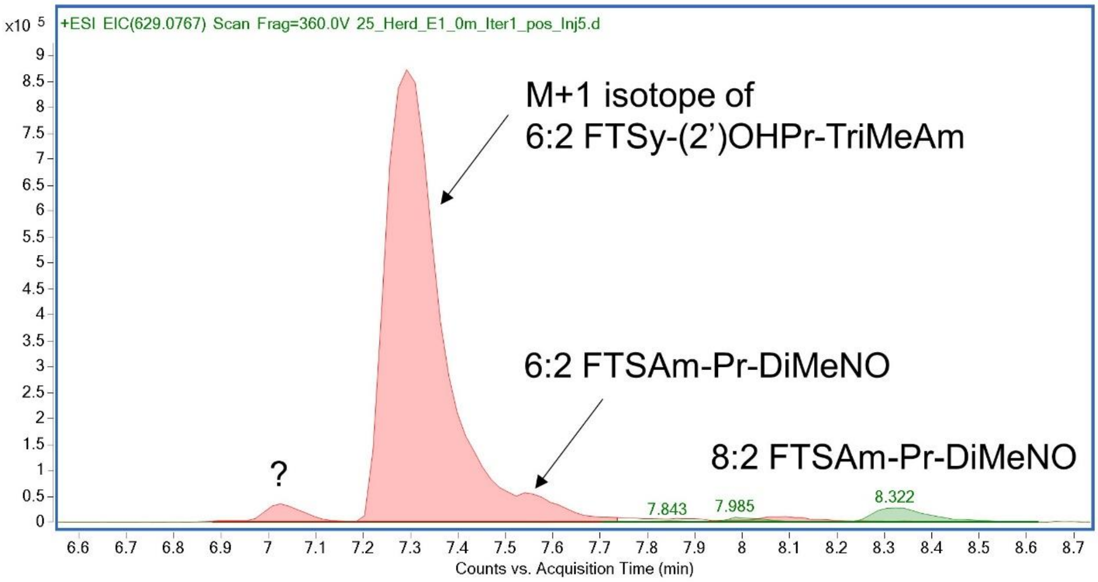


**Fig. S38b:** Chromatogram (ESI+, soil *S1*, 0 – 0.5 m, first extract) of n:2 FTSAm-Pr-DiMeNOs (n = 6 (red, m/z 529.0825, ≈ 7.57 min) and 8 (green, m/z 629.0761, 8.322 min)).

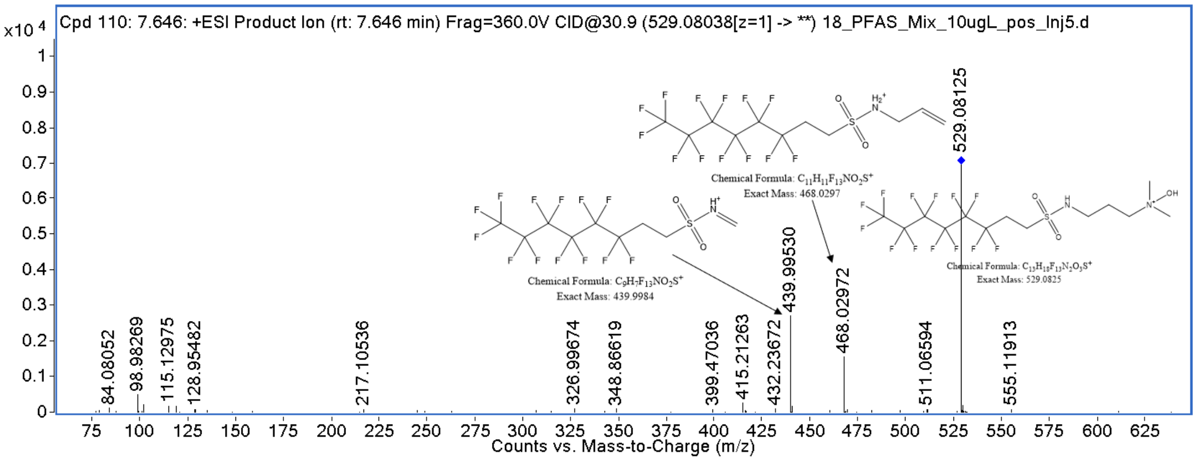


**Fig. S38c:** MS[2] spectrum (ESI+, 30.9 eV, reference standard) of 6:2 FTSAm-Pr-DiMeNO (m/z 529.0825, 7.646 min). Note: there was no MS[2] for 6:2 FTSAm-Pr-DiMeNO in the soil sample available in ESI+ due to overlapping of the M+1 peak of 6:2 FTSy-(2')OHPr-TriMeAm (compare Fig. S9 (b)) and no MS[2] was available in the negative mode due to low peak intensity.

**n:2 FTSAm-Pr-MeAms (n = 6)**

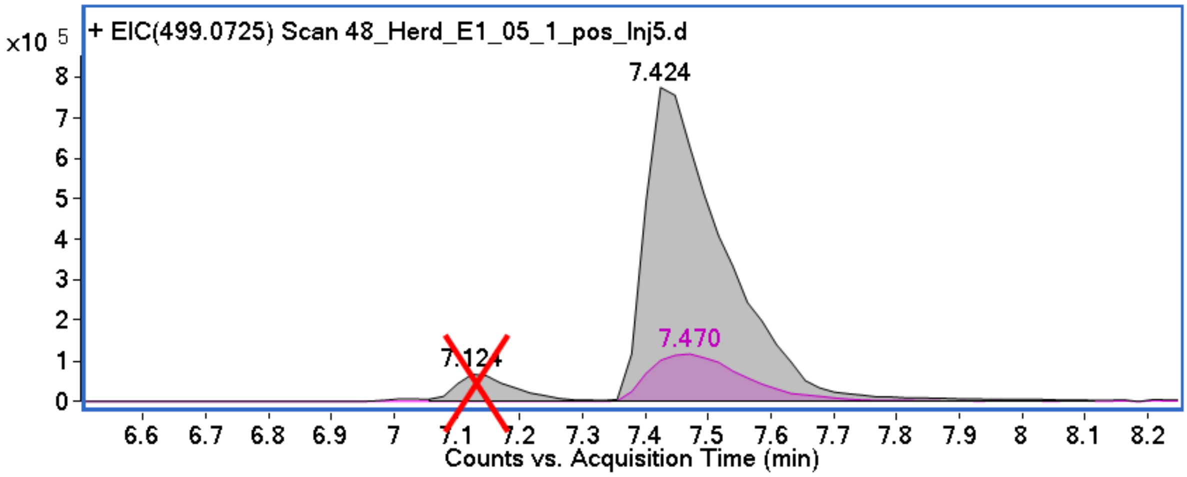


**Fig. S39a:** Chromatogram (ESI$^-$/ESI$^+$, soil *S1*, 0.5 – 1 m, first extract) of 6:2 FTSAm-Pr-MeAms in negative (pink, m/z 497.0574, 7.570 min) and positive (grey, m/z 499.0719, 7.424 min) ionization mode. Note: The first peak of 6:2 FTSAm-Pr-MeAm in positive mode is crossed out as it belongs to PFHxSAm-Pr-TriMeAm.

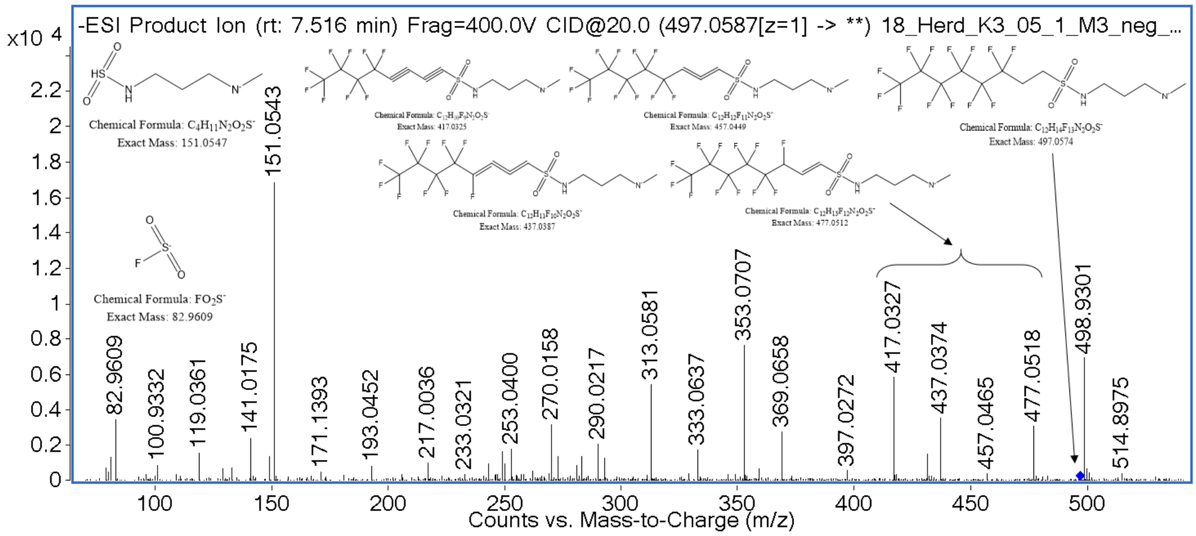


**Fig. S39b:** MS$^2$ spectrum (ESI$^-$, 20.0 eV, soil *S1*, 0.5 – 1 m, combined extract, targeted MS$^2$) of 6:2 FTSAm-Pr-MeAm (m/z 497.0574, 7.516 min).

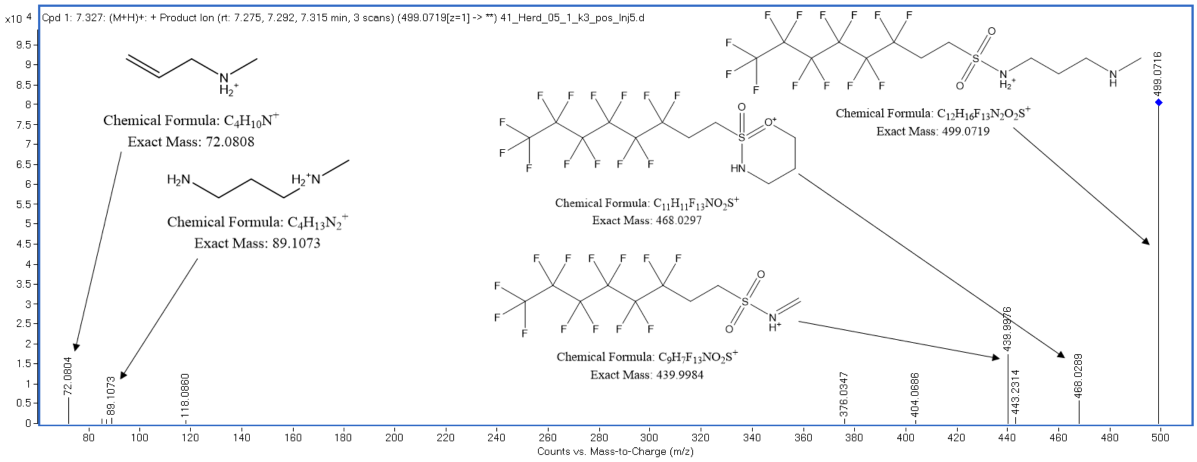


**Fig. S39c:** MS$^2$ spectrum (ESI$^+$, 30.0 eV, soil *S1*, 0.5 – 1 m, combined extract) of 6:2 FTSAm-Pr-MeAm (m/z 499.0719, 7.275, 7.292, and 7.315 min).

**n:2 FTSAm-Pr-DiMeAms (n = 6, 8)**

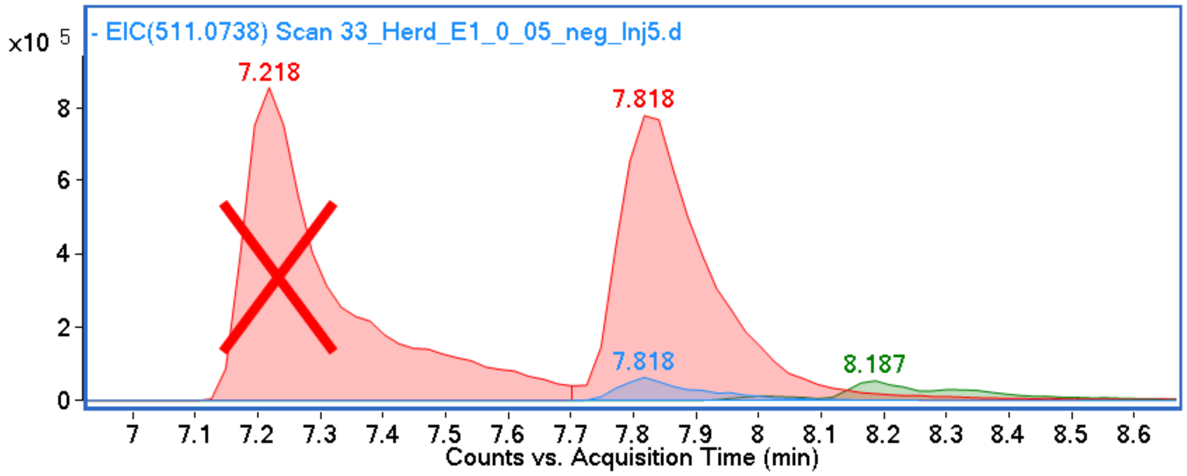


**Fig. S40a:** Chromatogram (ESI-/ESI+, soil *S1*, 0 – 0.5 m, first extract) of 6:2 FTSAm-Pr-DiMeAm in negative (blue, m/z 511.0730, 7.818 min) and positive (red, m/z 513.0876, 7.818 min) ionization mode and 8:2 FTSAm-Pr-DiMeAm in positive (green, m/z 613.0812, 8.187 min) ionization mode. Note: First red peak is crossed out as it is the M+1 peak of 6:2 FTSO-(2')OHPr-TriMeAm.

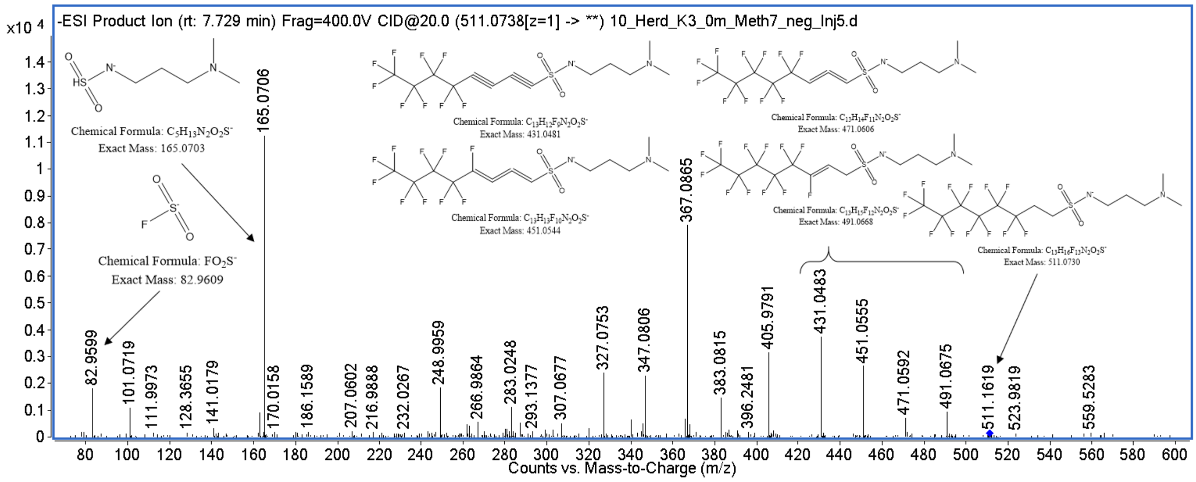


**Fig. S40b:** MS$^2$ spectrum (ESI$^-$, 20.0 eV soil *S1*, 0 – 0.5 m, combined extract, targeted MS$^2$) of 6:2 FTSAm-Pr-DiMeAm (m/z 511.0730, 7.729 min).

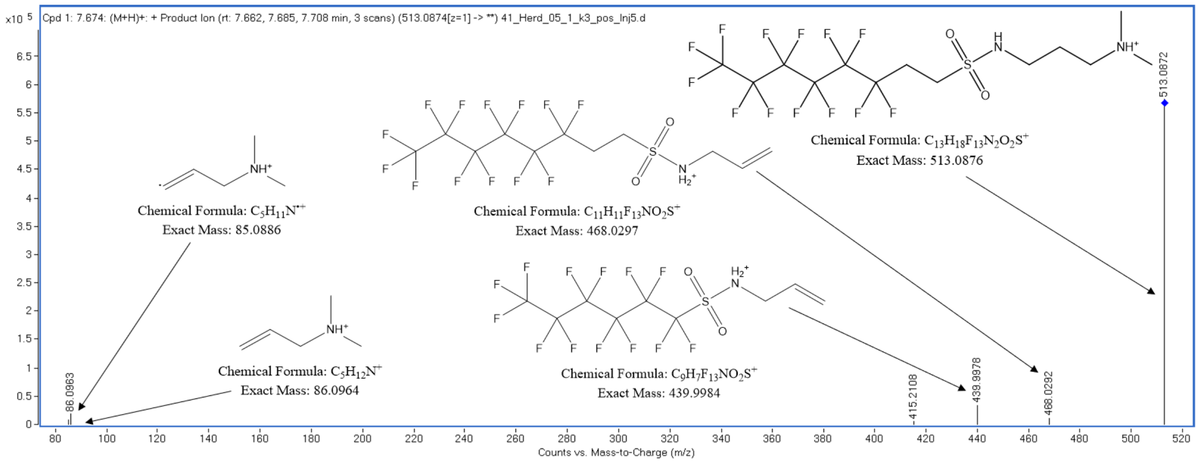


**Fig. S40c:** MS$^2$ spectrum (ESI$^+$, 30.4 eV, soil *S1*, 0.5 – 1 m, combined extract) of 6:2 FTSAm-Pr-DiMeAm (m/z 513.0876, 7.662, 7.685, and 7.708 min).

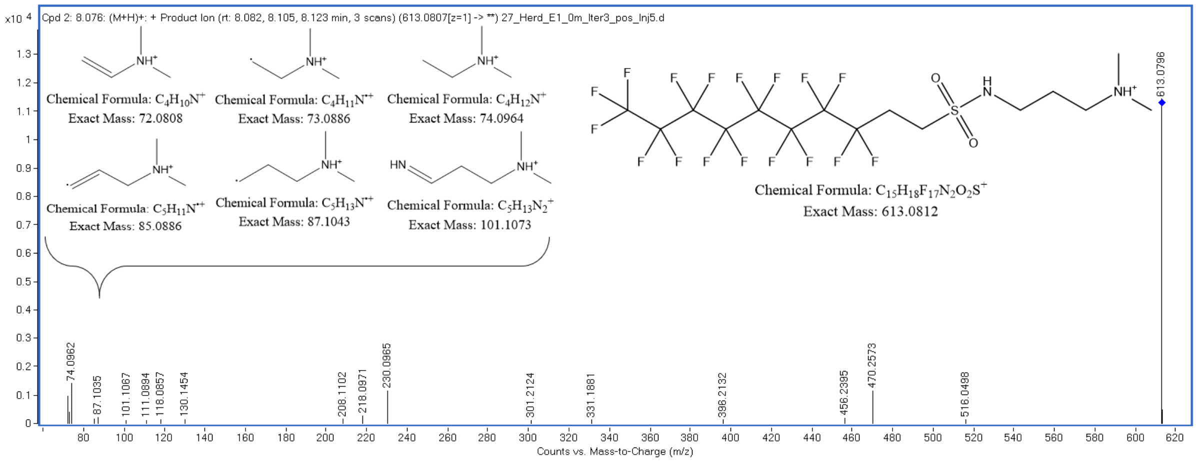

**Fig. S40d:** MS$^2$ spectrum (ESI$^+$, 33.4 eV, soil *S1*, 0 – 0.5 m, first extract, iterative MS$^2$) of 8:2 FTSAm-Pr-DiMeAm (m/z 613.0812, 8.082, 8.105, and 8.123 min).

# 6. FTSy-derivatives

## n:2 FTSy-PrAs (n = 4, 6, 8)

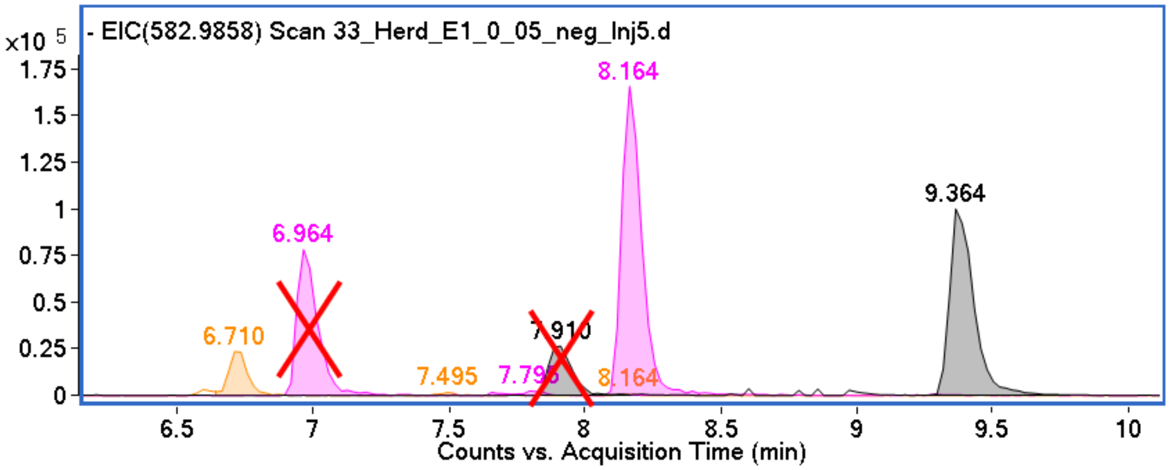


**Fig. S41a:** Chromatogram (ESI$^-$, soil *S1*, 0 – 0.5 m, first extract) of n:2 FTSy-PrAs (n = 4 (orange, m/z 383.0005, 6.710 min), 6 (pink, m/z 482.9941, 8.164 min), and 8 (grey, m/z 582.9877, 9.364 min)). Note: Two peaks are crossed out as they are in-source adducts of n:2 FTSAs (n = 6 and 8).

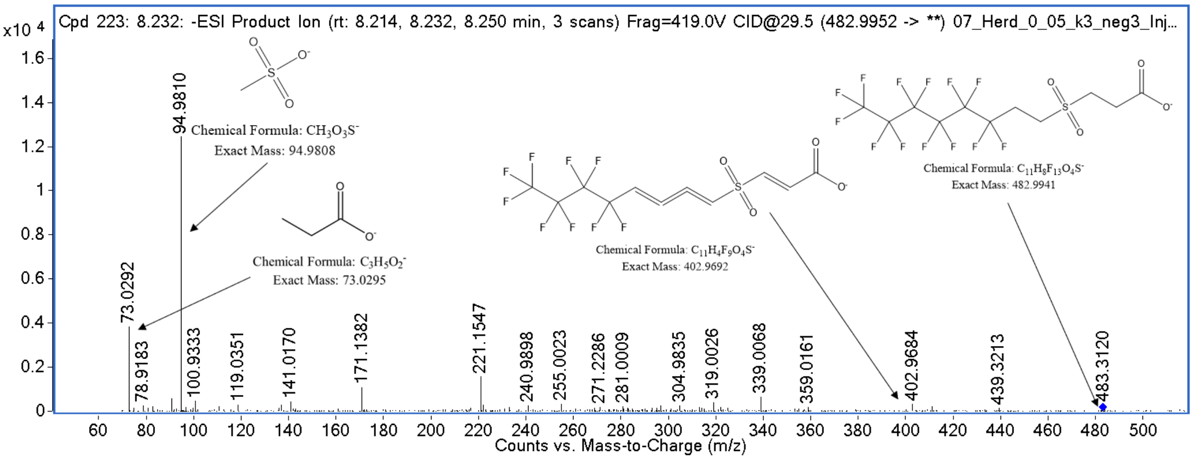


**Fig. S41b:** MS$^2$ spectrum (ESI$^-$, soil *S1*, 0 – 0.5 m, combined extract, iterative MS$^2$) of 6:2 FTSy-PrA (m/z 482.9941, 8.214, 8.232, and 8.250 min).

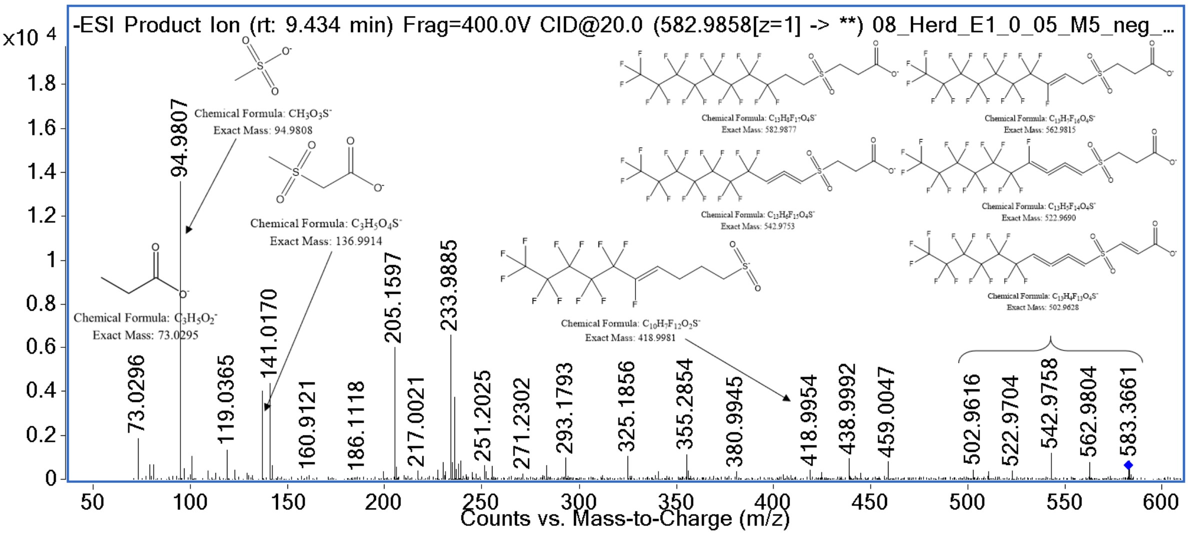


**Fig. S41c:** MS$^2$ spectrum (ESI$^-$, 20.0 eV, soil *S1*, 0 – 0.5 m, first extract, targeted MS$^2$) of 8:2 FTSy-PrA (m/z 582.9877, 9.434 min).

### n:2 FTSy-Pr-Ad-(5',5')DiMeEtSAs (n = 6, 8, 10,12)

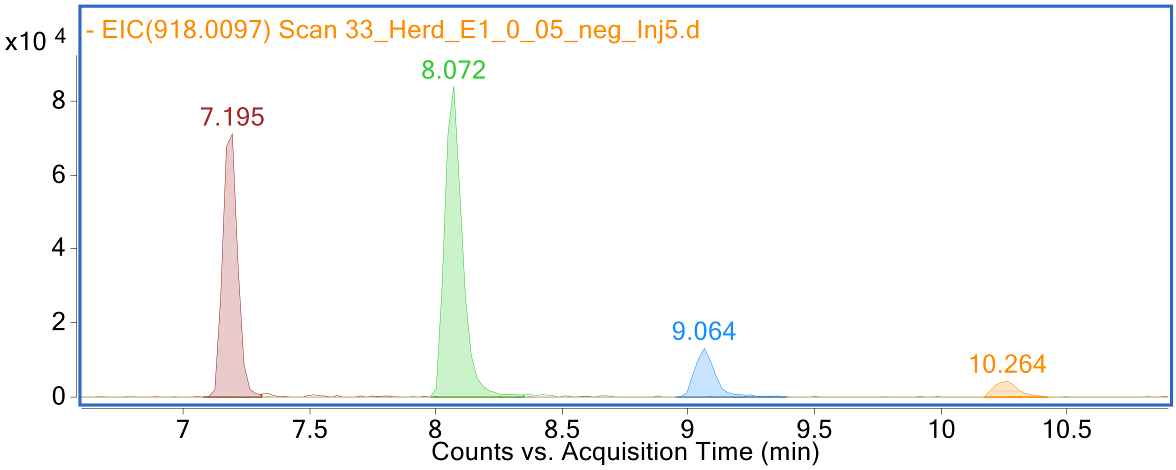


**Fig. S42a:** Chromatogram (ESI$^-$, soil *S1*, 0 – 0.5 m, first extract) of n:2 FTSy-Pr-Ad-(5',5')DiMeEtSAs (n = 6 (red, m/z 618.0295, 7.195 min), 8 (green, m/z 718.0231, 8.072 min), 10 (blue, m/z 818.0167, 9.064 min), and 12 (orange, m/z 918.0104, 10.264 min)).

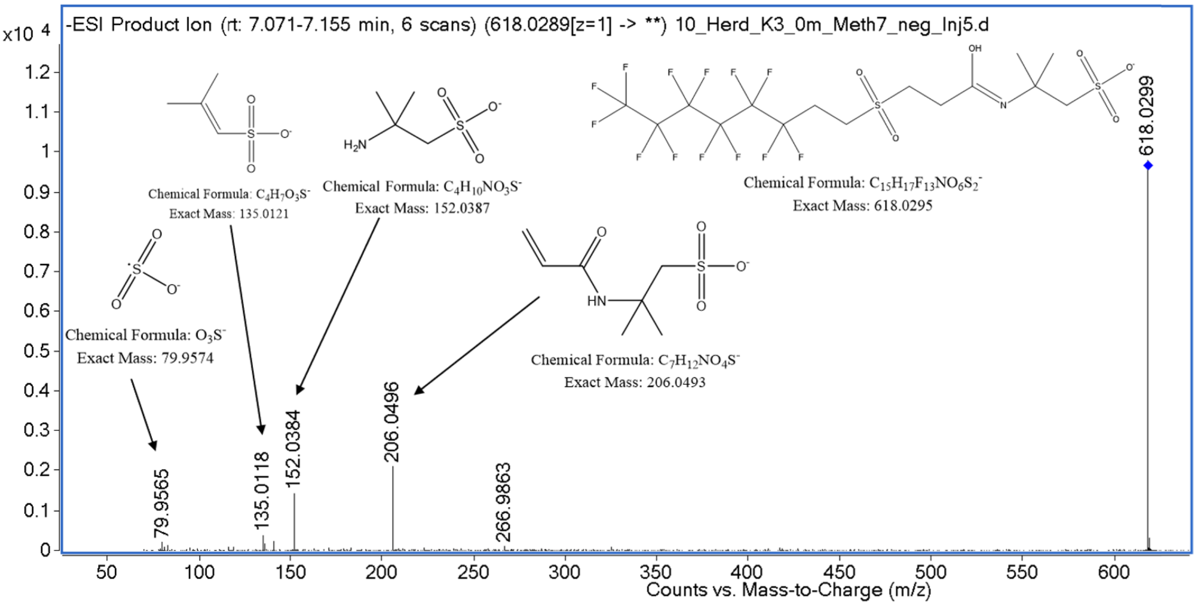


**Fig. S42b:** $MS^2$ spectrum (ESI$^-$, different CEs (10 – 40 eV), soil *S1*, 0 – 0.5 m, combined extract, targeted $MS^2$) of 6:2 FTSy-Pr-Ad-(5',5')DiMeEtSA (m/z 618.0295, 7.071 – 7.155 min).

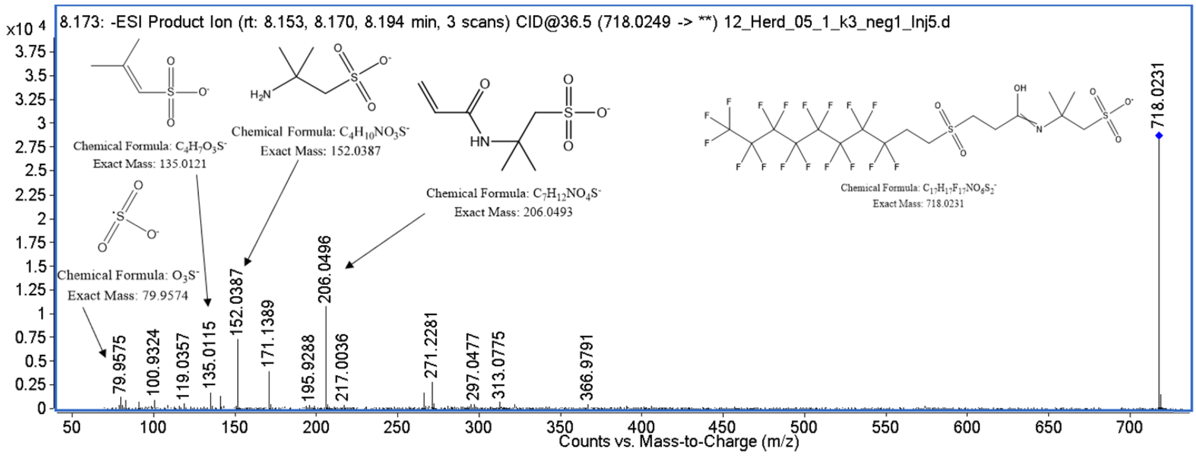


**Fig. S42c:** $MS^2$ spectrum (ESI$^-$, 36.5 eV, soil *S1*, 0.5 – 1 m, combined extract, iterative $MS^2$) of 8:2 FTSy-Pr-Ad-(5',5')DiMeEtSA (m/z 718.0231, 8.153, 8.170, and 8.194 min).

# 7. FTSO-derivatives

## n:2 FTSO-(2')OHPr-TriMeAms (n = 4, 6, 8)

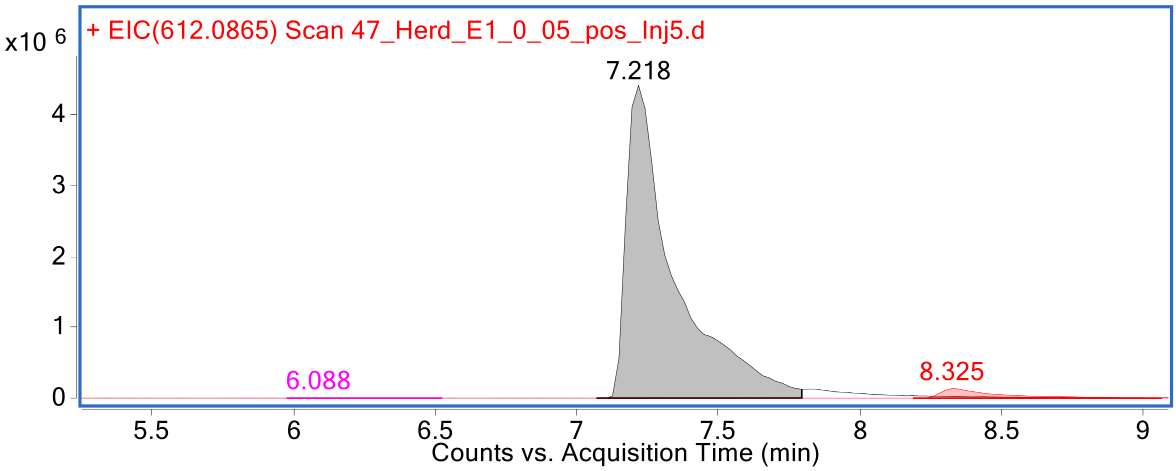


**Fig. S43a:** Chromatogram (ESI$^+$, soil *S1*, 0 – 0.5 m, first extract) of n:2 FTSO-(2')OHPr-TriMeAms (n = 4 (pink, m/z 412.0987, 6.088 min), 6 (grey, m/z 512.0923, 7.218 min), and 8 (red, m/z 612.0860, 8.325 min)).

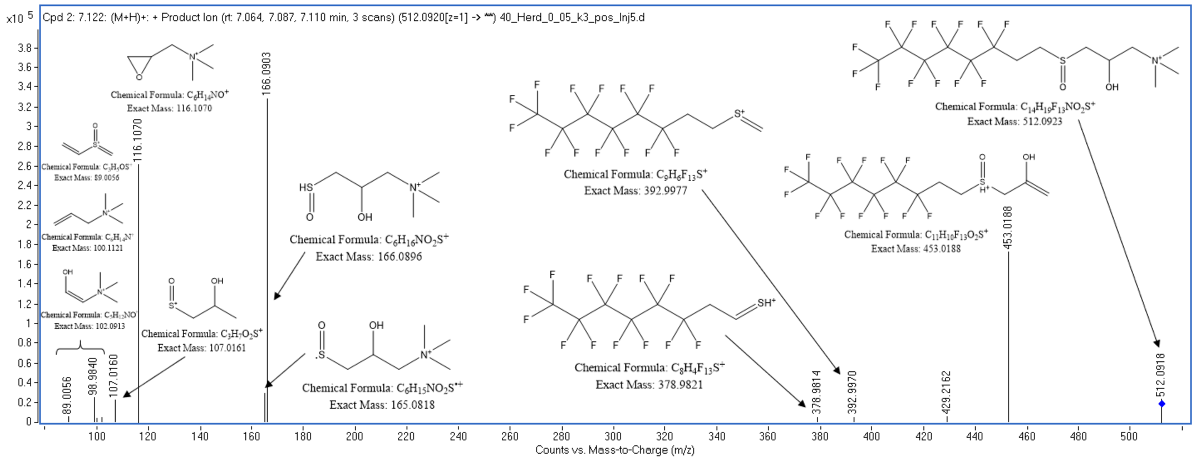


**Fig. S43b:** MS$^2$ spectrum (ESI$^+$, 30.4 eV, soil *S1*, 0 – 0.5 m, combined extract) of 6:2 FTSO-(2')OHPr-TriMeAm (m/z 512.0923, 7.064, 7.087, and 7.110 min).

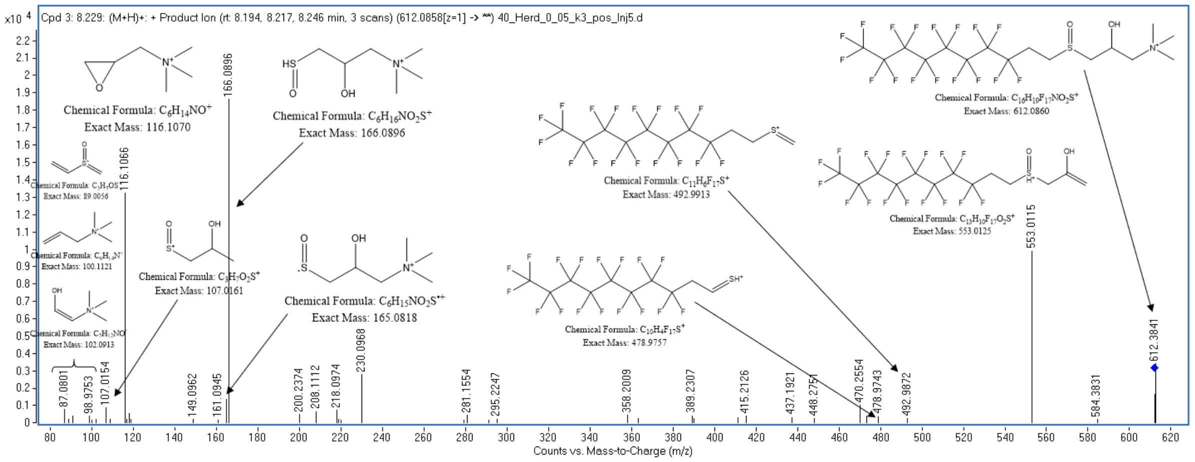


**Fig. S43c:** MS$^2$ spectrum (ESI$^+$, 33.4 eV, soil *S1*, 0 – 0.5 m, combined extract) of 8:2 FTSO-(2')OHPr-TriMeAm (m/z 612.0860, 8.194, 8.217, and 8.246 min).

## n:2 FTSO-Pr-Ad-(5’,5’)DiMeEtSAs (n = 6)

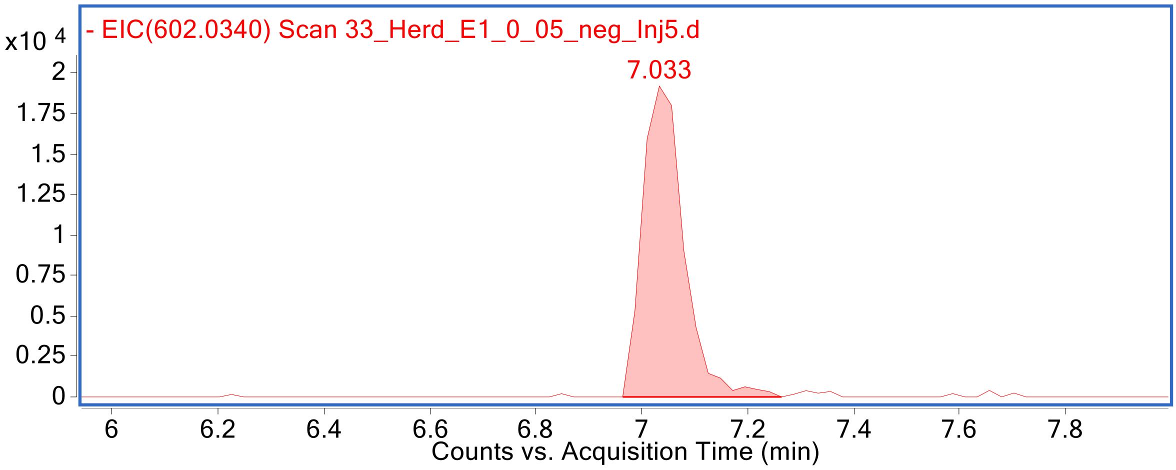


**Fig. S44a:** Chromatogram (ESI$^-$, soil *S1*, 0 – 0.5 m, first extract) of 6:2 FTSO-Pr-Ad-(5’,5’)DiMeEtSA (m/z 602.0346, 7.033 min).

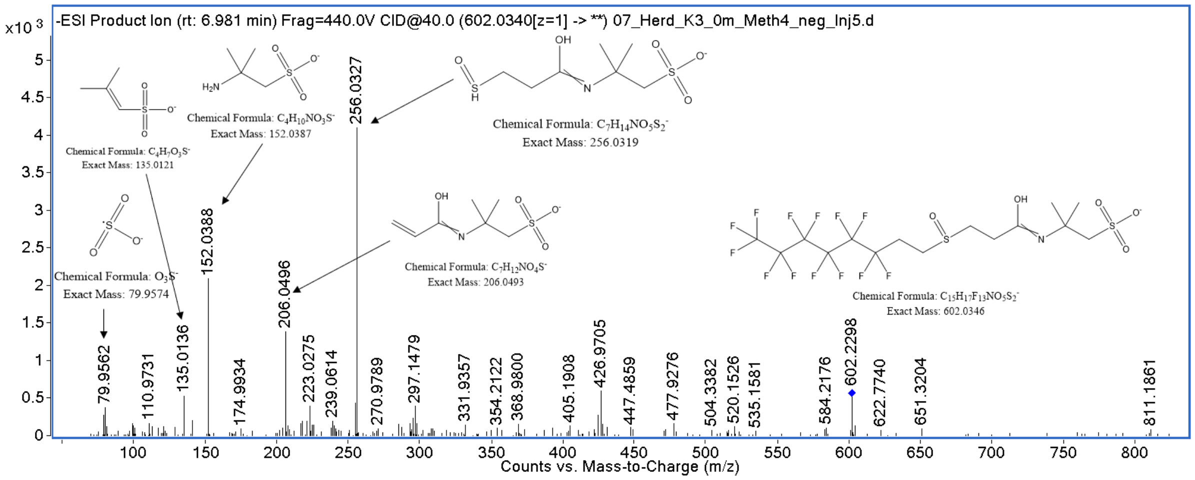


**Fig. S44b:** MS$^2$ spectrum (ESI$^-$, 40.0 eV, soil *S1*, 0 – 0.5 m, combined extract, targeted MS$^2$) of 6:2 FTSO-Pr-Ad-(5’,5’)DiMeEtSA (m/z 602.0346, 6.981 min).

# 8. FTTh-derivatives

### n:2 FTTh-(2')OHPr-TriMeAms (n = 4, 6, 8, 10)

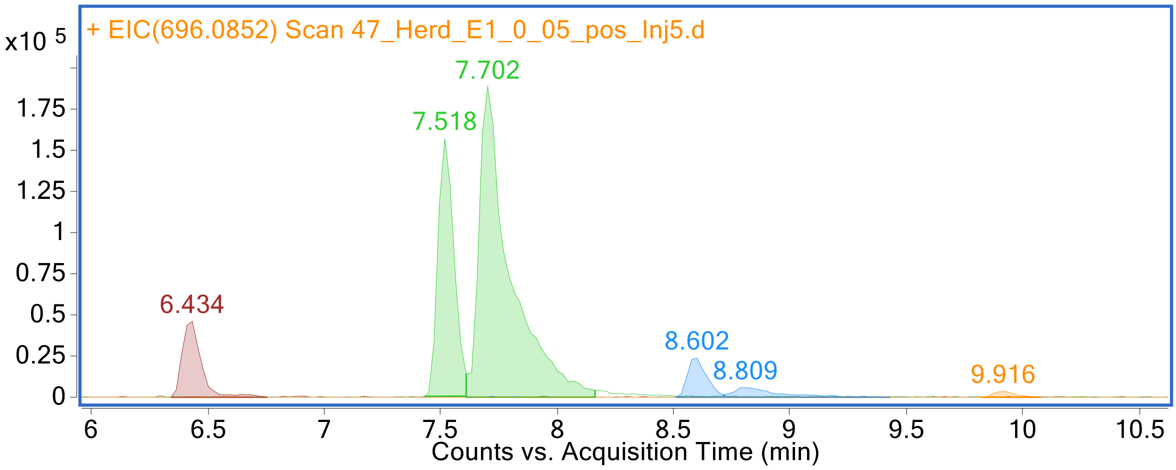


**Fig. S45a:** Chromatogram (ESI$^+$, soil *S1*, 0 – 0.5 m, first extract) of n:2 FTTh-(2')OHPr-TriMeAms (n = 4 (red, m/z 396.1038, 6.434 min), 6 (green, m/z 496.0974, 7.518 and 7.702 min), 8 (blue, m/z 596.0910, 8.602 and 8.809 min), and 10 (orange, m/z 696.0847, 9.916 min)).

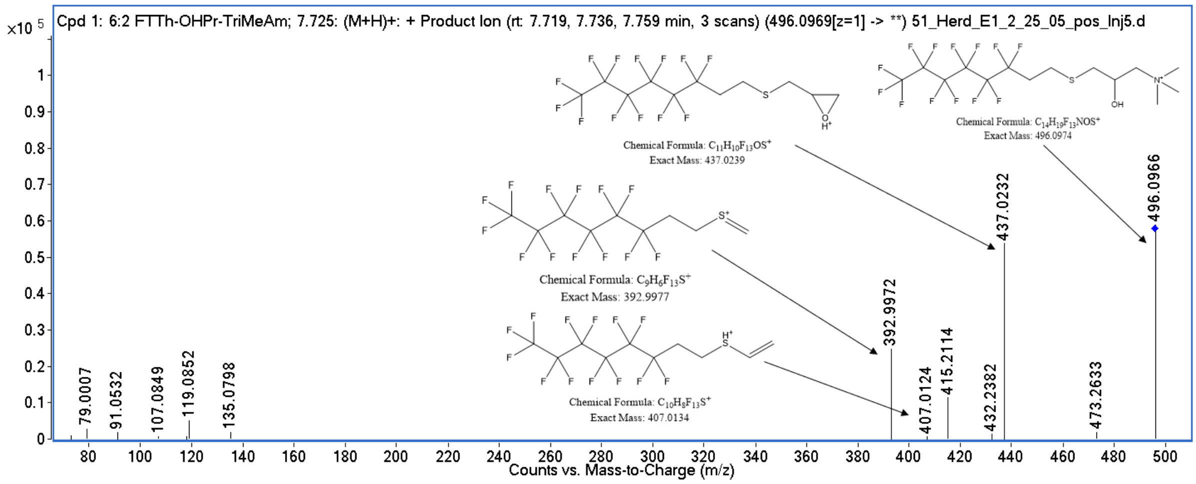


**Fig. S45b:** MS$^2$ spectrum (ESI$^+$, 29.9 eV, soil *S1*, 2 – 2.5 m, first extract) of 6:2 FTTh-(2')OHPr-TriMeAm (m/z 496.0974, 7.719, 7.736, and 7.759 min).

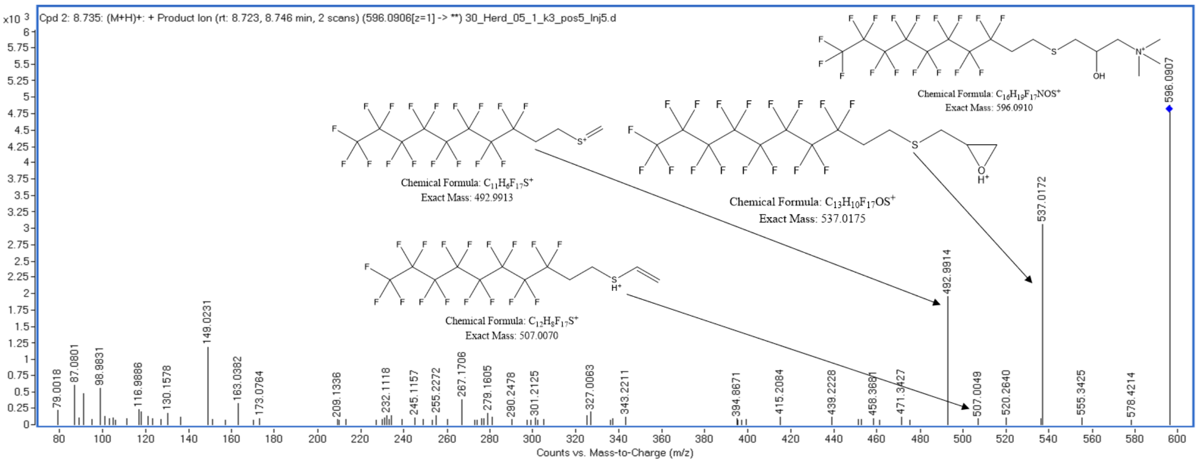


**Fig. S45c:** MS$^2$ spectrum (ESI$^+$, 32.9 eV, soil *S1*, 0.5 – 1 m, combined extract, iterative MS$^2$) of 8:2 FTTh-(2')OHPr-TriMeAm (m/z 596.0910, 8.723 and 8.746 min).

# 9. FTSA-derivatives

## OH-n:2 FTSAs (n = 6, 8)

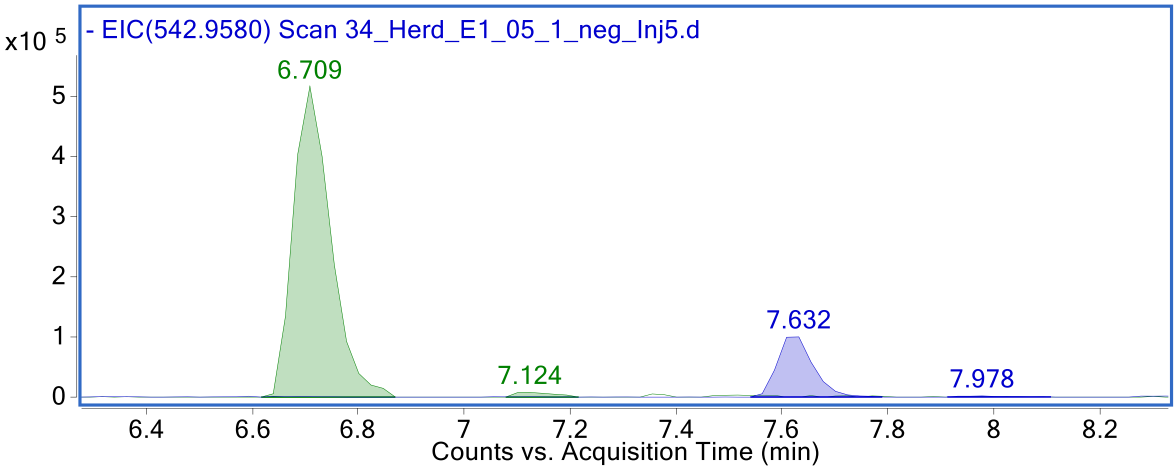


**Fig. S46a:** Chromatogram (ESI$^-$, soil *S1*, 0.5 – 1 m, first extract) of OH-n:2 FTSAs (n = 6 (green, m/z 442.9628, 6.709 min) and 8 (blue, m/z 542.9564, 7.632 min)).

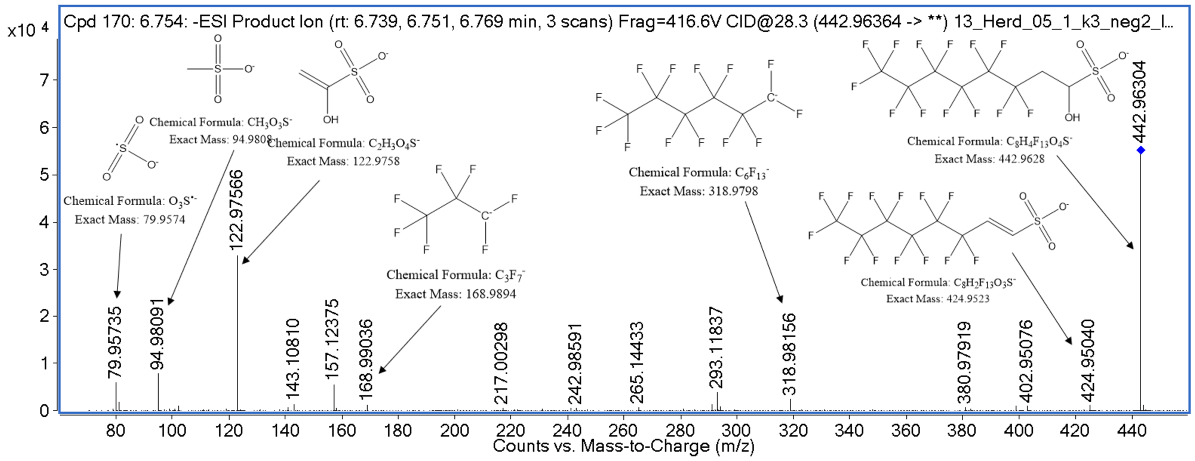


**Fig. S46b:** MS$^2$ spectrum mode (ESI$^-$, 28.3 eV, soil *S1*, 0.5 – 1 m, combined extract, iterative MS$^2$) of OH-6:2 FTSA (m/z 442.9628, 6.739, 6.751, and 6.796 min).

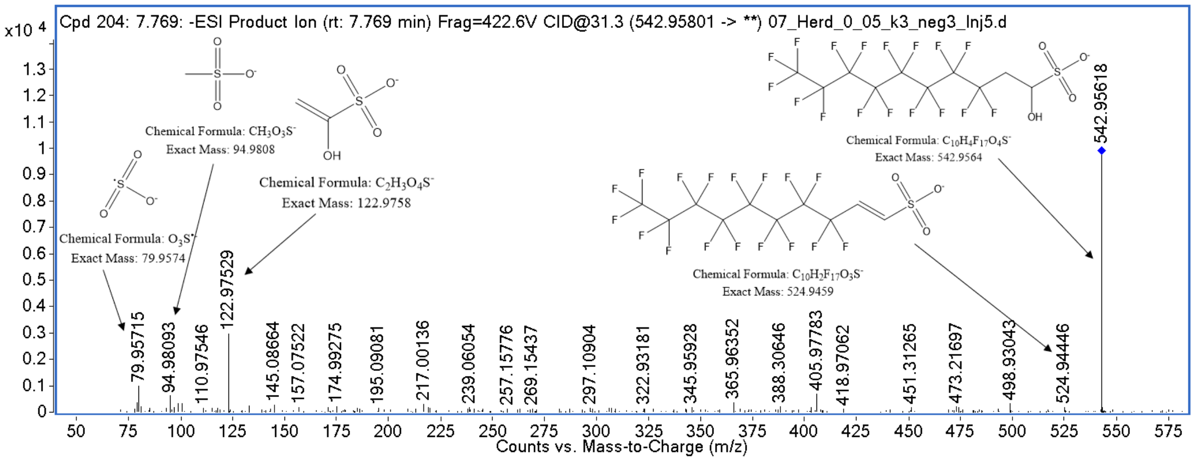


**Fig. S46c:** MS$^2$ spectrum (ESI$^-$, 31.3 eV, soil *S1*, 0 – 0.5 m, combined extract, iterative MS$^2$) of OH-8:2 FTSA (m/z 542.9564, 7.769 min).

# Confidence level 4

### E-PFSAs (n = 10-17)

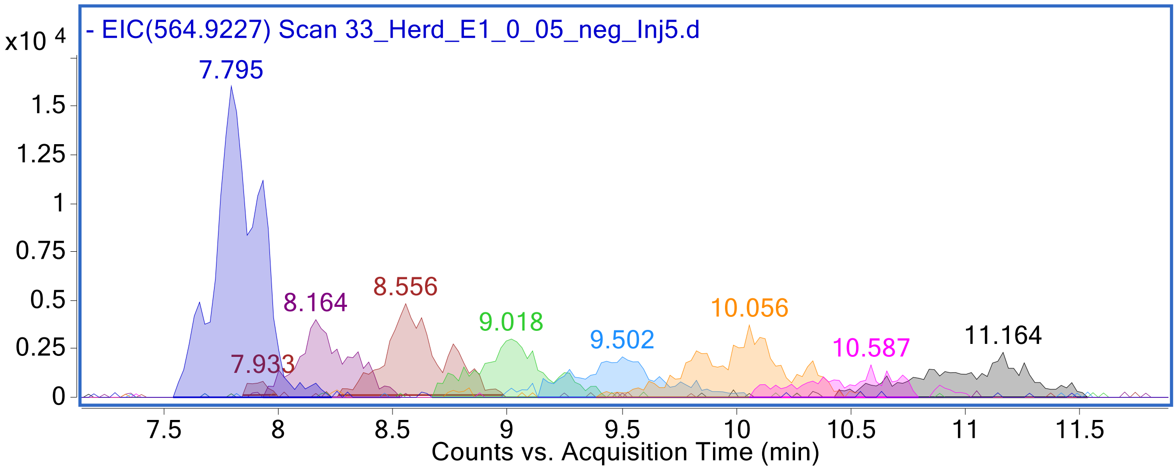


**Fig. S47:** Chromatogram (ESI$^-$, soil *S1*, 0 – 0.5 m, first extract) of E-PFSAs (n = 10 (dark blue, m/z 564.9219, 7.795 min), 11 (violet, m/z 614.9187, 8.164 min), 12 (dark red, m/z 664.9156, 8.556 min), 13 (green, m/z 714.9124, 9.018 min), 14 (blue, m/z 764.9092, 9.502 min), 15 (orange, m/z 814.9060, 10.056 min), 16 (pink, m/z 864.9028, 10.587 min), and 17 (grey, m/z 914.8996, 11.164 min)).

### PFASAm-*N*-Me-*N*-EtAs (or isomers) (n = 8, 13, 15)

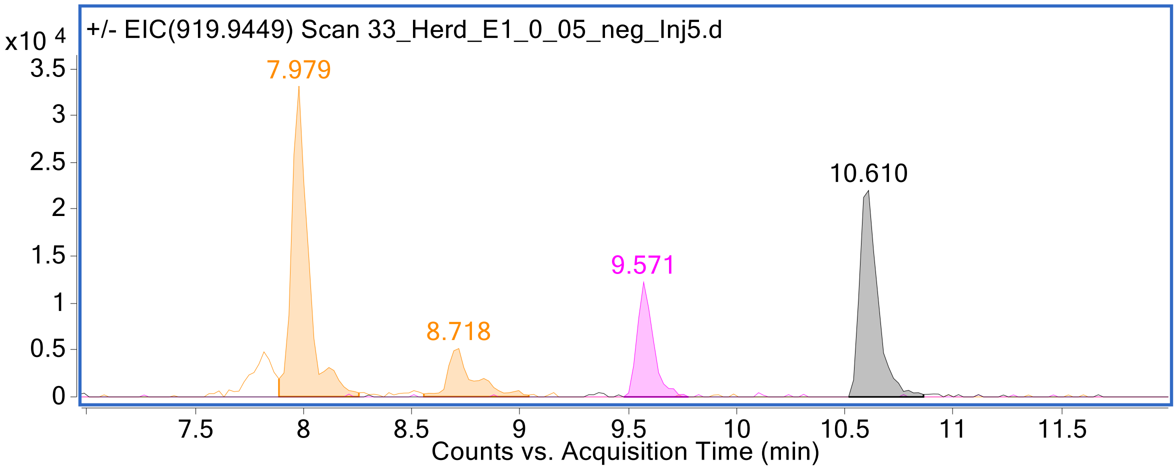


**Fig. S48:** Chromatogram (ESI$^-$, soil *S1*, 0 – 0.5 m, first extract) of PFASAm-*N*-Me-*N*-EtAs (or isomers) (n = 8 (orange, m/z 569.9673, 7.979 min and/or 8.718 min), 13 (pink, m/z 819.9514, 9.571 min), and 15 (grey, m/z 919.9450, 10.610 min)).

**PFASAm-*N*-Et-*N*-EtA and PFASAm-*N*-Me-*N*-PrA (n = 6, 8)**

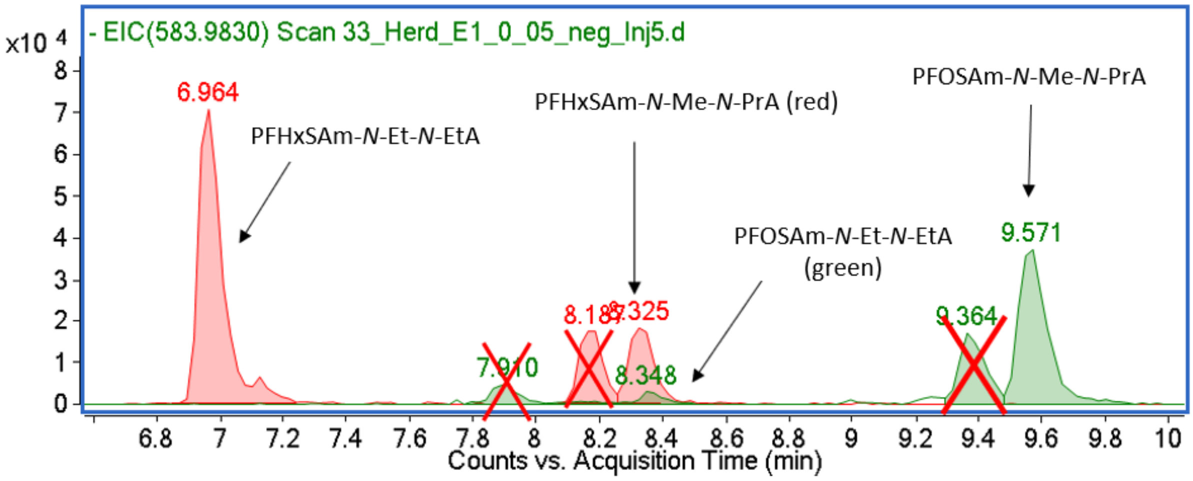


**Fig. S49:** Chromatogram (ESI-, soil *S1*, 0 – 0.5 m, first extract) of PFASAm-*N*-Et-*N*-EtA (n = 6 (red, m/z 483.9894, 6.964 min) and 8 (green, m/z 583.9830, 8.348 min)) and PFASAm-*N*-Me-*N*-PrA (n = 6 (red, m/z 483.9894, 8.325 min) and 8 (green, m/z 583.9830, 9.571 min)). Note: Three peaks are crossed out as they are the M+1 peaks of n:2 FTSy-PrAs (n = 6 and 8).

**n:2 FTSAm-EtOHs (n = 6)**

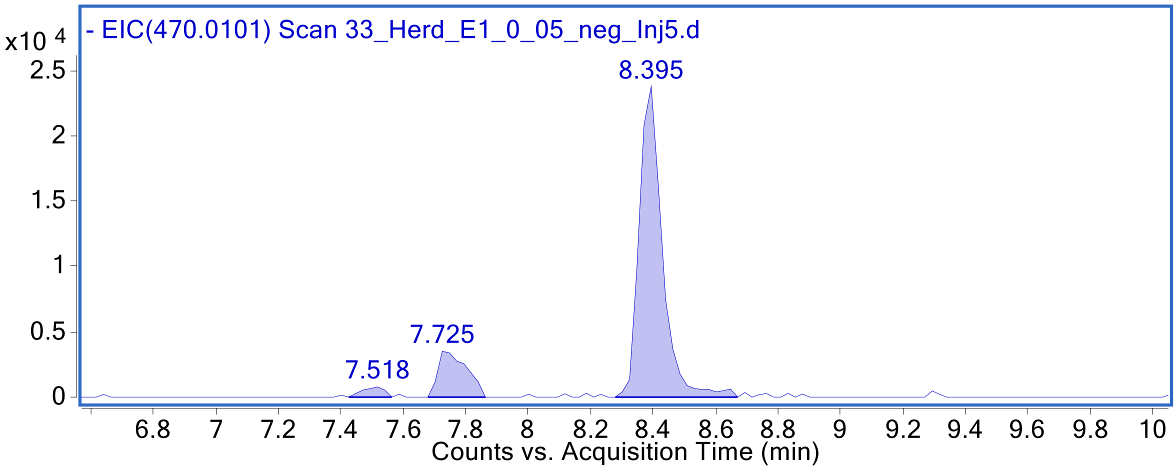


**Fig. S50:** Chromatogram (ESI-, soil *S1*, 0 – 0.5 m, first extract) of 6:2 FTSAm-EtOH (m/z 470.0096, 8.395 min).

**n:2 FTSAm-KAmPes (n = 6)**

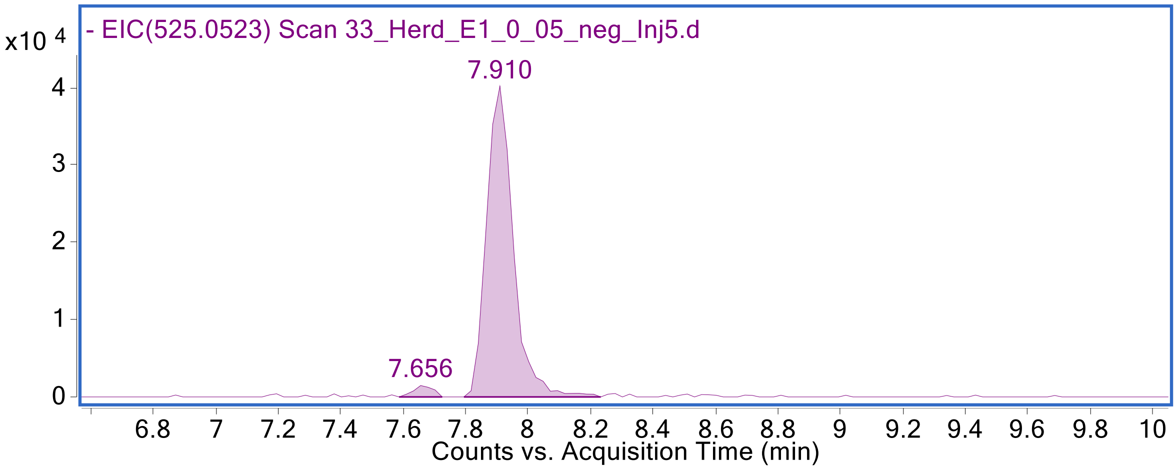


**Fig. S51:** Chromatogram (ESI-, soil *S1*, 0 – 0.5 m, first extract) of 6:2 FTSAm-KAmPes (m/z 525.0518, 7.910 min).

**n:2 FTTh-Pr-Ad-(5',5')DiMeEtSAs (n = 6)**

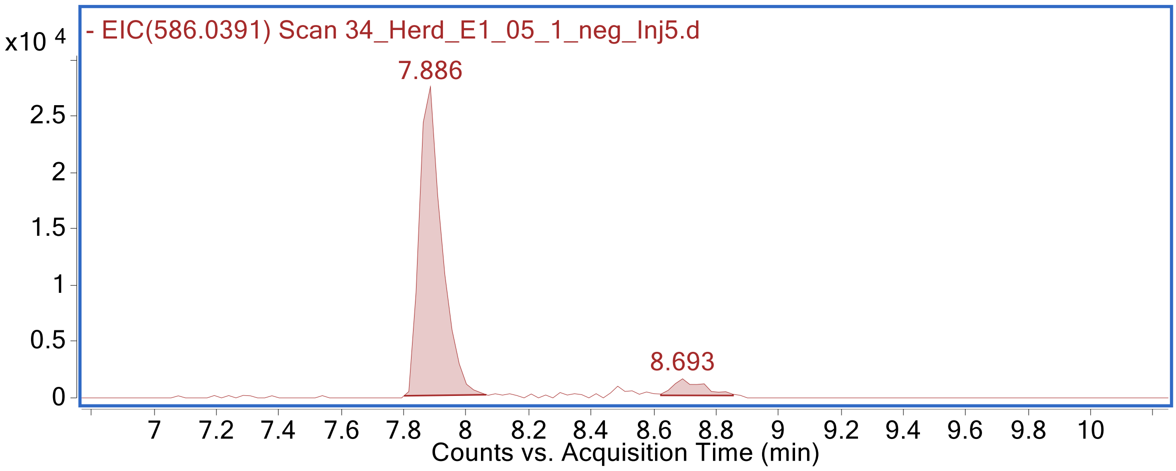


**Fig. S52:** Chromatogram (ESI-, soil *S1*, 0.5 – 1 m, first extract) of 6:2 FTTh-Pr-Ad-(5',5')DiMeEtSAs (m/z 586.0397, 7.886 min)

**U-n:3 FTAds (n = 5, 7)**

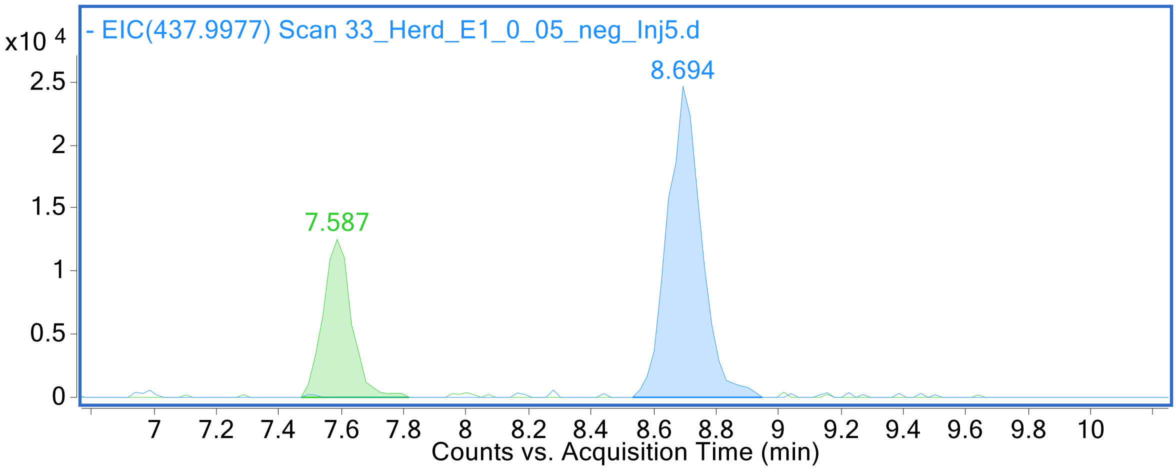


**Fig. S53:** Chromatogram (ESI-, soil *S1*, 0 – 0.5 m, first extract) of U-n:3 FTAds (n = 5 (green, m/z 338.0040, 7.587 min) and 7 (blue, m/z 437.9981, 8.694 min)).

# Confidence level 5

### H-E-PFSAs (n = 9, 11)

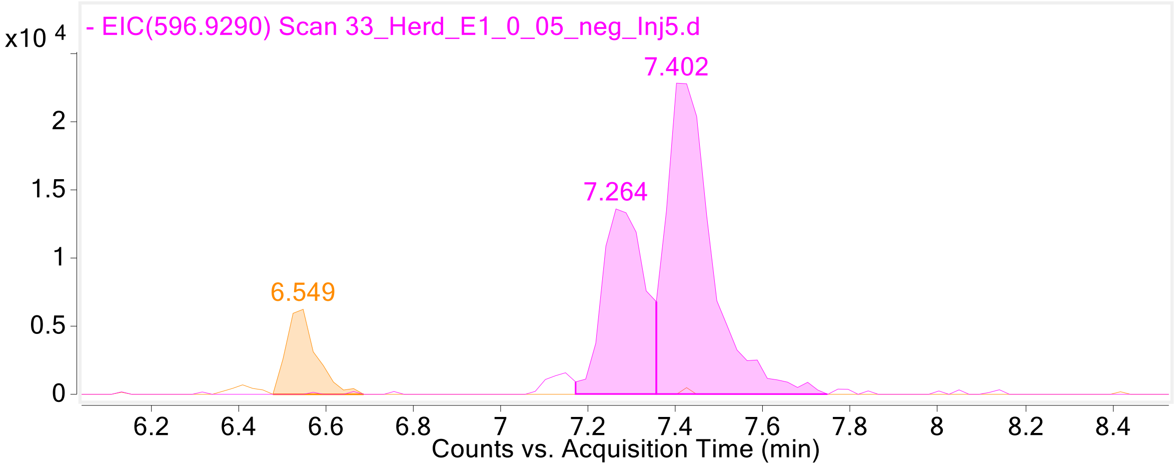


**Fig. S54:** Chromatogram (ESI$^-$, soil *S1*, 0 – 0.5 m, first extract) of H-E-PFSAs (n = 9 (orange, m/z 496.9346, 6.549 min) and 11 (pink, m/z 596.9282, 7.264 and 7.402 min)).

### n/m PFASAm dimers (n/m = 3/3, 3/4, 4/4, 4/5, 5/5, 5/6, 6/6)

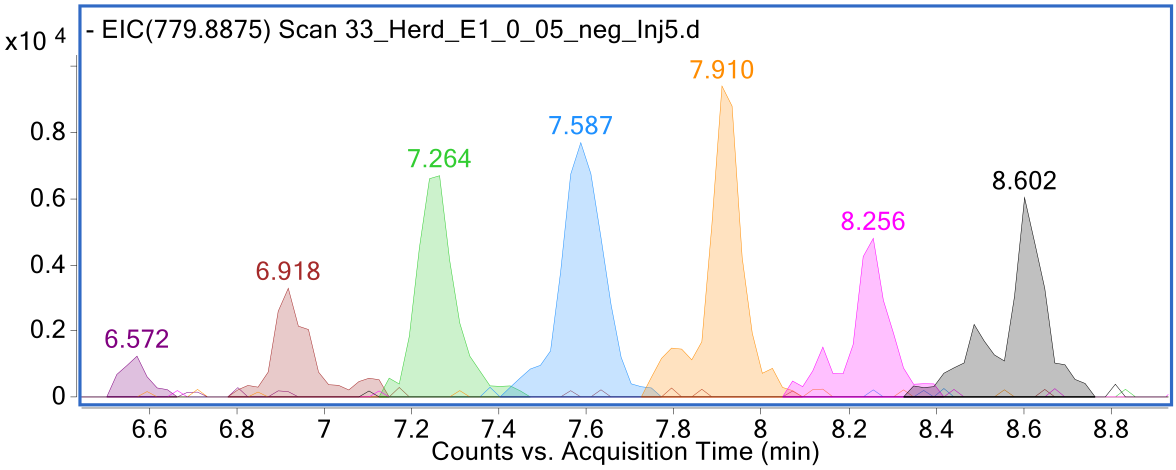


**Fig. S55:** Chromatogram (ESI$^-$, soil *S1*, 0 – 0.5 m, first extract) of n/m PFASAm dimers (n/m = 3/3 (violet, m/z 479.9051, 6.572 min), 3/4 (red, m/z 529.9019, 6.918 min), 4/4 (green, m/z 579.8987, 7.264 min), 4/5 (blue, m/z 629.8955, 7.587 min), 5/5 (orange, m/z 679.8923, 7.910 min), 5/6 (pink, m/z 729.8891, 8.256 min), and 6/6 (grey, m/z 779.8859, 8.602 min)).

**n:2 FTSAm-Ets (n = 6)**

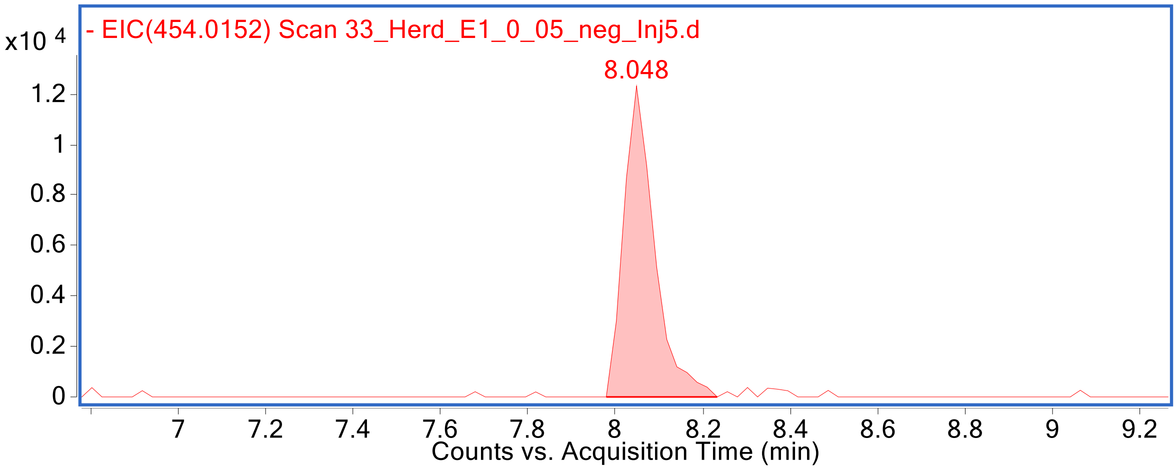


**Fig. S56:** Chromatogram (ESI$^-$, soil *S1*, 0 – 0.5 m, first extract) of 6:2 FTSAm-Et (m/z 454.0147, 8.048 min).

**n:2 FTSAm-EtAls (n = 6)**

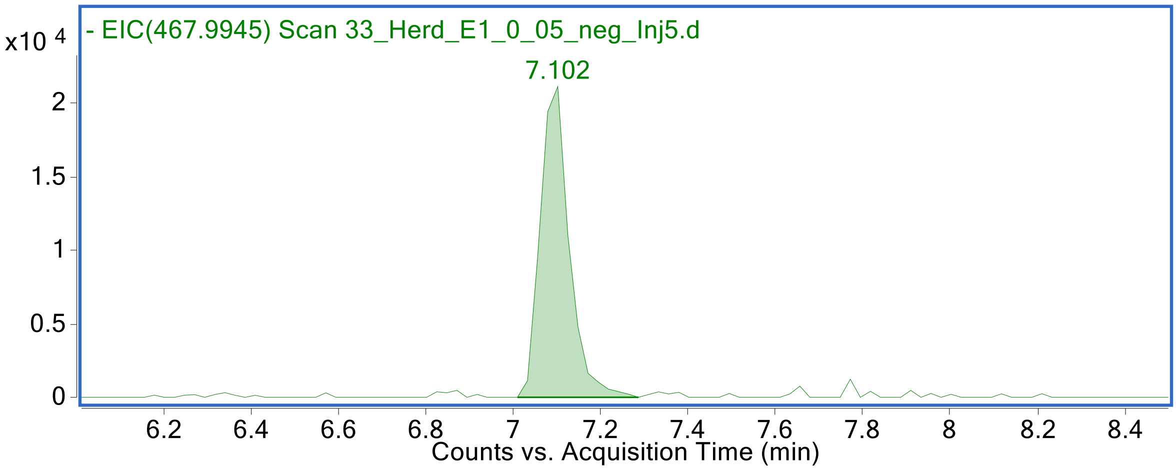


**Fig. S57:** Chromatogram (ESI$^-$, soil *S1*, 0 – 0.5 m, first extract) of 6:2 FTSAm-EtAl (m/z 467.9940, 7.102 min).

**n:2 FTSAm-KAmMePes (n = 6)**

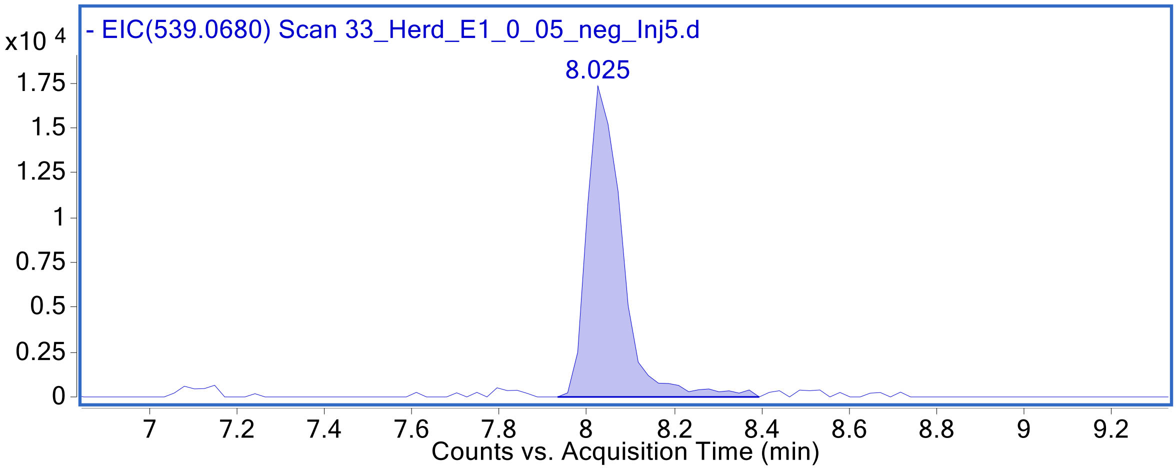


**Fig. S58:** Chromatogram (ESI$^-$, soil *S1*, 0 – 0.5 m, first extract) of 6:2 FTSAm-KAmMePe (m/z 539.0675, 8.025 min).

# References


**1.** Place B (2021) Suspect List of Possible Per- and Polyfluoroalkyl Substances (PFAS). National Institute of Standards and Technology. https://data.nist.gov/od/id/mds2-2387. Accessed 2023-12-15

**2.** Charbonnet JA, McDonough CA, Xiao F, Schwichtenberg T, Cao D, Kaserzon S, Thomas KV, Dewapriya P, Place BJ, Schymanski EL, Field JA, Helbling DE, Higgins CP (2022) Communicating Confidence of Per- and Polyfluoroalkyl Substance Identification via High-Resolution Mass Spectrometry. Environ Sci Technol Lett 9 (6):473-481. doi:10.1021/acs.estlett.2c00206

**3.** Nickerson A, Maizel AC, Kulkarni PR, Adamson DT, Kornuc JJ, Higgins CP (2020) Enhanced Extraction of AFFF-Associated PFASs from Source Zone Soils. Environ Sci Technol 54 (8):4952-4962. doi:10.1021/acs.est.0c00792

**4.** Rotander A, Karrman A, Toms LM, Kay M, Mueller JF, Gomez Ramos MJ (2015) Novel fluorinated surfactants tentatively identified in firefighters using liquid chromatography quadrupole time-of-flight tandem mass spectrometry and a case-control approach. Environ Sci Technol 49 (4):2434-2442. doi:10.1021/es503653n
